# “Interchangeability” of PD-L1 immunohistochemistry assays: a meta-analysis of diagnostic accuracy

**DOI:** 10.1038/s41379-019-0327-4

**Published:** 2019-08-05

**Authors:** Emina Torlakovic, Hyun J. Lim, Julien Adam, Penny Barnes, Gilbert Bigras, Anthony W. H. Chan, Carol C. Cheung, Jin-Haeng Chung, Christian Couture, Pierre O. Fiset, Daichi Fujimoto, Gang Han, Fred R. Hirsch, Marius Ilie, Diana Ionescu, Chao Li, Enrico Munari, Katsuhiro Okuda, Marianne J. Ratcliffe, David L. Rimm, Catherine Ross, Rasmus Røge, Andreas H. Scheel, Ross A. Soo, Paul E. Swanson, Maria Tretiakova, Ka F. To, Gilad W. Vainer, Hangjun Wang, Zhaolin Xu, Dirk Zielinski, Ming-Sound Tsao

**Affiliations:** 1 Saskatchewan Health Authority (SHA), Saskatoon, SK Canada; 2 College of Medicine, University of Saskatchewan, Saskatoon, SK Canada; 3 Gustave-Roussy Cancer Campus, Villejuif, France; 4 Dalhousie University, Halifax, NS Canada; 5 Cross Cancer Institute, Edmonton, AB Canada; 6 The Chinese University of Hong Kong, New Territories, Hong Kong; 7 University Health Network, Toronto, ON Canada; 8 University of Toronto, Toronto, ON Canada; 9 Seoul National University Bundang Hospital, Seongnam, Gyeonggi-do Republic of Korea; 10 Institut universitaire de cardiologie et de pneumologie de Québec - Université Laval (IUCPQ-UL), Quebec City, QC Canada; 11 McGill University Health Science Centre, Montreal, QC Canada; 12 Kobe City Medical Center General Hospital, Kobe, Japan; 13 School of Public Health, Texas A&M University, College Station, TX USA; 14 Center for Thoracic Oncology, Mount Sinai Cancer, Mount Sinai Health System, New York, NY USA; 15 Hôpital Pasteur, FHU OncoAge, Biobanque BB-0033-00025, Université Côte d’Azur, CHU de Nice, Nice, France; 16 British Columbia Cancer Agency, Vancouver, BC Canada; 17 Fujian Cancer Hospital and Fujian Medical University Cancer Hospital, Fuzhou, Fujian China; 18 IRCCS Sacro Cuore Don Calabria Hospital, Negrar, Verona Italy; 19 Nagoya City University Graduate School of Medical Science, Nagoya, Japan; 20 Precision Medicine and Genomics, AstraZeneca, Cambridge, UK; 21 Yale University School of Medicine, New Haven, CT USA; 22 Hamilton Health Sciences, McMaster University, Hamilton, ON Canada; 23 Aalborg University Hospital, Aalborg, Denmark; 24 University Hospital Cologne, Institute of Pathology, Cologne, Germany; 25 National University Hospital, Singapore, Singapore; 26 University of Washington, Seattle, WA USA; 27 Cumming School of Medicine, University of Calgary, Calgary, AB Canada; 28 Tel Aviv Sourasky Medical Center, Tel Aviv, Israel; 29 McGill University Health Center and McGill University, Montreal, QC Canada; 30 TARGOS Molecular Pathology GmbH, Kassel, Germany

**Keywords:** Non-small-cell lung cancer, Predictive markers, Immunohistochemistry

## Abstract

Different clones, protocol conditions, instruments, and scoring/readout methods may pose challenges in introducing different PD-L1 assays for immunotherapy. The diagnostic accuracy of using different PD-L1 assays interchangeably for various purposes is unknown. The primary objective of this meta-analysis was to address PD-L1 assay interchangeability based on assay diagnostic accuracy for established clinical uses/purposes. A systematic search of the MEDLINE database using PubMed platform was conducted using “PD-L1” as a search term for 01/01/2015 to 31/08/2018, with limitations “English” and “human”. 2,515 abstracts were reviewed to select for original contributions only. 57 studies on comparison of two or more PD-L1 assays were fully reviewed. 22 publications were selected for meta-analysis. Additional data were requested from authors of 20/22 studies in order to enable the meta-analysis. Modified GRADE and QUADAS-2 criteria were used for grading published evidence and designing data abstraction templates for extraction by reviewers. PRISMA was used to guide reporting of systematic review and meta-analysis and STARD 2015 for reporting diagnostic accuracy study. CLSI EP12-A2 was used to guide test comparisons. Data were pooled using random-effects model. The main outcome measure was diagnostic accuracy of various PD-L1 assays. The 22 included studies provided 376 2×2 contingency tables for analyses. Results of our study suggest that, when the testing laboratory is not able to use an Food and Drug Administration-approved companion diagnostic(s) for PD-L1 assessment for its specific clinical purpose(s), it is better to develop a properly validated laboratory developed test for the same purpose(s) as the original PD-L1 Food and Drug Administration-approved immunohistochemistry companion diagnostic, than to replace the original PD-L1 Food and Drug Administration-approved immunohistochemistry companion diagnostic with a another PD-L1 Food and Drug Administration-approved companion diagnostic that was developed for a different purpose.

## Introduction

Clinical trials have shown that it is possible to successfully restore host immunity against various malignant neoplasms even in advanced stage disease by deploying drugs that target the PD-1/PD-L1 axis [1–5]. In most of these studies, higher expression of PD-L1 was associated with a more robust clinical response, suggesting that detection of PD-L1 expression could be used as predictive biomarker. However, anti-PD1/PD-L1 therapy companies developed distinct immunohistochemistry protocols for assessing a single biomarker (PD-L1 expression), as well as different scoring schemes for the readouts. The latter include differences in the cell type assessed for the expression and different cut-off points as thresholds [2–4]. “Intended use” in this context is a part of the so-called “3D” concept, where a “fit-for-purpose” approach to test development and validation establishes explicit links between Disease, Drug, and Diagnostic assay [6].

Several such fit-for-purpose immunohistochemistry kits are commercially available, but in clinical practice, and especially in publicly funded health care, it is challenging to make all such testing available to patients [7–9]. Because of the great need to simplify testing, either by reducing the number of immunohistochemistry assays being used or the number of interpretative schemes employed or both, many studies have been conducted that compared the analytical performance of the various immunohistochemistry PD-L1 assays to determine if they might be deemed “interchangeable”. The concordance in the analytical performance of the immunohistochemistry assays and scoring algorithms derived from these studies have been reviewed by Büttner et al. and Udall et al. respectively [7, 10]. Although most of these studies have compared different PD-L1 immunohistochemistry assays to one another, there is little guidance on how the results of these studies may be applied clinically.

The goal of this study is to assess the performance of PD-L1 immunohistochemistry assays based on their diagnostic accuracy at specific cut-points, as defined for specific immunotherapies according to the clinical efficacy demonstrated in their respective pivotal clinical trials.

In other words, given an Food add Drug Administration-cleared assay, which other assays can be considered substantially equivalent for that specific purpose? Although comparison of immunohistochemistry assays for their analytical similarities is warranted and useful for clinical immunohistochemistry laboratories, it is an insufficient foundation on which to make an informed decision whether an Food and Drug Administration-approved companion diagnostic with a specific clinical purpose can be replaced by another assay, whether the substitute assay is an Food and Drug Administration-approved companion diagnostic for a different purpose or a laboratory developed test. The more appropriate approach for these qualitative assays would be comparing the results of the candidate assay for its diagnostic accuracy against a comparative method/assay or designated reference standard [11]. We report here the results of our meta-analyses of 376 assay comparisons from 22 studies for different cut-off points, focusing on the sensitivity and specificity of these tests, based on their intended clinical utility.

## Methods

Methodology including data sources, study selection, data abstraction, and grading evidence, are detailed in Supplementary Files Methodology. Modified GRADE and QUADAS-2 criteria were used for grading published evidence and designing data abstraction templates to guide independent extraction by multiple reviewers [12–15]. PRISMA was used to guide reporting of the systematic review and meta-analysis and STARD 2015 for reporting the diagnostic accuracy study [16–18]. CLSI EP12-A2 was used to guide test comparisons [11] Data were pooled using a random-effects model.

### Framework

A systematic review of literature was conducted as a part of a national project for developing Canadian guidelines for PD-L1 testing. The Canadian Association of Pathologists – Association canadienne des pathologistes (CAP-ACP) National Standards Committee for High Complexity Testing initiated development of CAP-ACP Guidelines for PD-L1 testing to facilitate introduction of PD-L1 testing for various purposes to Canadian clinical immunohistochemistry laboratories. This review was also used to guide the selection of publications to be used in this meta-analysis.

### Purpose-based approach

The purposes identified in the systematic review of published literature were based on either the clinical purpose that was specifically identified in the published study or the intended purpose for which the included specific companion diagnostic assay was clinically validated. Although a large number of potential purposes were identified, only few could be included in this meta-analysis. The selection was based on the type of data available, including which immunohistochemistry protocols and which readout was performed by the authors. The greatest limitation in the accrual of data from these published studies was based on the selection of the readout employed to assess the results; in most studies the readout was limited to tumor proportion score with 1% and 50% cut-offs, which is essentially based on the clinically meaningful cut-offs for pembrolizumab and nivolumab therapy. Hence, these two readouts were selected for our analysis and form the basis for outlining different purposes that are derived from the combination of the readouts and immunohistochemistry kits/protocols that use these readouts and are approved by regulatory agencies (e.g., Food and Drug Administration) for different clinical uses.

Most published studies on PD-L1 test comparison did not include 2 × 2 tables that would allow calculations of either diagnostic sensitivity and specificity or positive percent agreement and negative percent agreement. The CAP-ACP National Standards Committee for High Complexity Testing requested this information from the authors of studies where it was evident that the authors generated such results, but did not include them in their published manuscript. Most studies required generation of multiple 2 × 2 tables, as each one was designed for a specific purpose and set of candidate’ and ‘comparator’ assays. For primary studies that provided sufficient detail, information on study setting, comparative method/reference standard and 2 x 2 tables for different tumor proportion score cut-offs were extracted, from which accuracy results were reported. Studies of PD-L1 immunohistochemistry assay comparisons that did not compare the performance of the assay to any designated or potential reference standard (e.g., where analytical comparison of PD-L1 assays were all laboratory developed tests and/or no specific purpose was identified or where positive percent agreement and negative percent agreement could not be generated from study data) [19–22] were not included in this meta-analysis, because in such studies, diagnostic accuracy for a specific clinical purpose could not be determined. The acquisition of data resulted in cumulative evidence of 376 assay comparisons from 22 published studies [6, 23–43].

### Study tissue model(s)

Most studies evaluated PD-L1 immunohistochemistry in non-small cell lung cancer, resulting in 337 test comparisons. Comparisons of test performance in other tumors were much less common. These included analysis of urothelial carcinoma (20 test comparisons), mesothelioma (9 test comparisons) and thymic carcinoma (9 test comparisons).

### Meta-analysis

Reported or calculated diagnostic accuracy (sensitivity and specificity) from the individual studies were summarized. Random-effects models were fitted [44, 45]. For the qualitative review, a forest plot was used to obtain an overview of sensitivity and specificity for each study. Cochran’s heterogeneity statistics Q and I^2^ were used to examine heterogeneity among studies. Funnel plots and Egger’s test were applied to detect possible publication bias (see Supplementary Files for images of funnel plots) [46, 47]. The significance level of 0.05 was set for all analyses. Meta-analysis was performed using software *Strata 15 SE*.

### Interpretation of results

#### Clinically acceptable diagnostic accuracy

For the purpose of this study, the immunohistochemistry candidate assays were considered to be acceptable for clinical applications if both sensitivity and specificity for the stated clinical purpose/application were ≥90% [48].

#### Applicability of meta-analysis results: Food and Drug Administrtion-approved immunohistochemistry kits vs. laboratory developed tests

Assuming that laboratories follow the instructions for use provided with Food and Drug Administration-approved or European CE-marked immunohistochemistry kits, the overall results of this meta-analysis could be considered highly representative and generalizable of Food and Drug Administration-approved assay performance and the diagnostic accuracy of that assay against a designated reference standard for the stated specific purpose in any laboratory. However, this assumption cannot be applied to the results of laboratory developed tests, because laboratory developed test immunohistochemistry protocol conditions were often different in different laboratories even when the same primary antibody was used, and the protocol was performed on the same automated instrument with the same detection system (e.g., different type and duration of antigen retrieval, primary antibody dilution or incubation time, number of steps of amplification, etc.). When the results of the meta-analysis for laboratory developed tests were suboptimal, but one or more laboratories achieved ≥90% sensitivity and specificity; we cannot exclude the possibility that with appropriate immunohistochemistry protocol modification and assay validation, other laboratories could also achieve optimal results. This contrasts with the use of Food and Drug Administration-approved assays, where no protocol modifications are allowed. Therefore, where the results of laboratory developed tests are excellent, they are representative of what could be achieved by laboratory developed tests rather than that they are generalizable and that they will automatically be achieved in all laboratories.

## Results

### Meta-analysis (all tissue models)

**Fig. 1:**
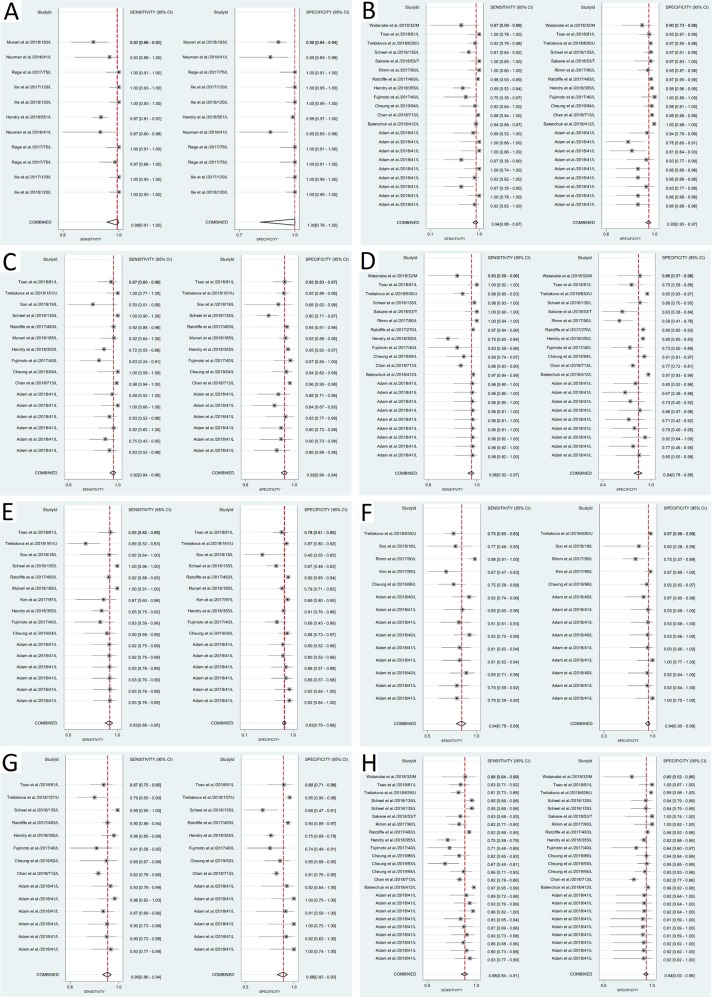
**a** 22C3 laboratory developed tests (candidate) vs. PD-L1 IHC pharmDx 22C3 (reference standard) for 1% tumor proportion score cut-off; **b** PD-L1 IHC pharmDx 28-8 (candidate) vs. PD-L1 IHC pharmDx 22C3 (reference standard) for 50% tumor proportion score cut-off; **c** Ventana PD-L1 (SP263) (candidate) vs. PD-L1 IHC pharmDx 22C3 (reference standard) for 50% tumor proportion score cut-off; **d** PD-L1 IHC pharmDx 28-8 (candidate) vs. PD-L1 IHC pharmDx 22C3 (reference standard) for 1% tumor proportion score cut-off; **e** Ventana PD-L1 (SP263) (candidate) vs. PD-L1 IHC pharmDx 22C3 (reference standard) for 1% tumor proportion score cut-off; **f** E1L3N laboratory developed tests (candidate) vs. PD-L1 IHC pharmDx 22C3 (reference standard) for 1% tumor proportion score cut-of; **g** Ventana PD-L1 (SP263) (candidate) vs. PD-L1 IHC pharmDx 28-8 (reference standard) for 1% tumor proportion score cut-off, and **h** PD-L1 IHC pharmDx 22C3 (candidate) vs. PD-L1 IHC pharmDx 28-8 (reference standard) for 1% tumor proportion score cut-off

The number of studies comparing different assays in this meta-analysis was larger than the number of published manuscripts, due to the frequent inclusion of multiple test comparisons in single publication as well as use of different cut-off points for “positive” vs. “negative” test result. Table 1A (non-small cell lung cancer), 1B (all tissue types), 2, and 3 summarize the number of studies that included both candidate and comparator test for a specific, clinically relevant purpose/cut-off point for a specific tissue model. Figures 1–13 illustrate forest plots with all studies using non-small cell lung cancer as tissue model (see Supplementary files for Figures 2–13). There was no significant difference in the results when non-small cell lung cancer studies were analyzed separately vs. meta-analysis of all tissue models (compare Table 1A to Table 1B).

**Table 1A:** Summary of NSCLC results from all studies (combined estimate of sensitivity and specificity)

TPS Cut-off	Gold Standard	Candidate Assay	No. of Comparisons	Sensitivity (95% CI)	Specificity (95% CI)
1%	PD-L1 IHC 22C3 pharmDx	22C3 LDT	11	0.99 (0.91–1.00)	1.00 (0.78–1.00)
		PD-L1 IHC 28-8 pharmDx	18	0.96 (0.93–0.98)	0.84 (0.77–0.88)
		Ventana PD-L1 (SP263)	15	0.93 (0.90–0.96)	0.82 (0.78–0.86)
		E1L3N LDT	13	0.84 (0.78–0.89)	0.92 (0.87–0.95)
		Ventana PD-L1 (SP142)	17	0.60 (0.53–0.66)	0.96 (0.93–0.98)
		28-8 LDT	6	See Table B	
		SP142 LDT	3	See Table C	
		SP263 LDT	2	See Table C	
		73-10 Assay	1	See Table C	
	PD-L1 IHC 28-8 pharmDx	Ventana PD-L1 (SP263)	13	0.91 (0.87–0.94)	0.87 (0.80–0.92)
		PD-L1 IHC 22C3 pharmDx	21	0.88 (0.84–0.92)	0.93 (0.91–0.95)
		28-8 LDT	4	0.82 (0.67–0.91)	0.91 (0.82- 0.96)
		E1L3N LDT	11	0.81 (0.77–0.85)	0.96 (0.89–0.99)
		Ventana PD-L1 (SP142)	12	0.57 (0.50–0.64)	0.99 (0.89–1.00)
		SP142 LDT	2	See Table C	
		SP263 LDT	2	See Table C	
		73-10 Assay	1	See Table C	
	Ventana PD-L1 (SP263)	PD-L1 IHC 28–8 pharmDx	13	0.93 (0.86–0.97)	0.84 (0.79–0.88)
		PD-L1 IHC 22C3 pharmDx	16	0.84 (0.77–0.89)	0.91 (0.85–0.94)
		E1L3N LDT	8	0.81 (0.75–0.86)	0.93 (0.85–0.96)
		Ventana PD-L1 (SP142)	12	0.57 (0.49–0.64)	0.98 (0.97–0.99)
		28–8 LDT	4	See Table B	
		SP142 LDT	1	See Table C	
		73-10 Assay	1	See Table C	
		22C3 LDT	1	See Table C	
50%	PD-L1 IHC 22C3 pharmDx	22C3 LDT	10	See Table B	
		PD-L1 IHC 28-8 pharmDx	18	0.94 (0.88–0.97)	0.95 (0.92–0.97)
		28-8 LDT	6	0.95 (0.81–0.99)	0.76 (0.67–0.83)
		Ventana PD-L1 (SP263)	15	0.91 (0.83–0.95)	0.92 (0.89–0.95)
		E1L3N LDT	13	0.76 (0.62–0.86)	0.97 (0.95–0.99)
		Ventana PD-L1 (SP142)	16	0.41 (0.29–0.53)	1.00 (0.99–1.00)
		SP142 LDT	3	See Table C	
		SP263 LDT	1	See Table C	
		73-10 Assay	1	See Table C	
	Ventana PD-L1 (SP263)	28-8 LDT	4	See Table B	
		PD-L1 IHC 28-8 pharmDx	12	0.68 (0.54–0.79)	0.98 (0.97–0.99)
		PD-L1 IHC 22C3 pharmDx	16	0.57 (0.45–0.69)	0.99 (0.97–1.00)
		Ventana PD-L1 (SP142)	10	See Table B	
		SP142 LDT	1	See Table C	
		22C3 LDT	1	See Table C	
		73-10 Assay	1	See Table C

**Table 1B:** Summary of results from all studies (combined estimate of sensitivity and specificity)

TPS Cut-off	Gold Standard	Candidate Assay	No. of Comparisons	Sensitivity (95% CI)	Specificity (95% CI)
1%	PD-L1 IHC 22C3 pharmDx	22C3 LDT	11	0.99 (0.91 -1.00)	1.00 (0.78–1.00)
		PD-L1 IHC 28-8 pharmDx	21	0.96 (0.92–0.97)	0.84 (0.78–0.88)
		Ventana PD-L1 (SP263)	16	0.93 (0.88–0.95)	0.83 (0.77–0.86)
		E1L3N LDT	14	0.84 (0.78–0.88)	0.94 (0.90–0.96)
		Ventana PD-L1 (SP142)	18	0.61 (0.55–0.67)	0.97 (0.94–0.98)
		28-8 LDT	6	See Table B	
		SP142 LDT	3	See Table C	
		SP263 LDT	2	See Table C	
		73-10 Assay	1	See Table C	
	PD-L1 IHC 28-8 pharmDx	Ventana PD-L1 (SP263)	14	0.90 (0.86–0.94)	0.88 (0.82–0.93)
		PD-L1 IHC 22C3 pharmDx	24	0.88 (0.84–0.91)	0.94 (0.92–0.96)
		28-8 LDT	4	0.82(0.67–0.91)	0.91 (0.82–0.96)
		E1L3N LDT	12	0.80 (0.76–0.84)	0.99 (0.94–0.99)
		Ventana PD-L1 (SP142)	13	0.59 (0.52–0.66)	0.99 (0.96–1.00)
		SP142 LDT	2	See Table C	
		SP263 LDT	2	See Table C	
		73-10 Assay	1	See Table C	
	Ventana PD-L1 (SP263)	PD-L1 IHC 28-8 pharmDx	14	0.93 (0.86–0.96)	0.85 (0.80–0.88)
		PD-L1 IHC 22C3 pharmDx	17	0.83 (0.76–0.89)	0.91 (0.85–0.94)
		E1L3N LDT	9	0.78 (0.71–0.84)	0.93 (0.88–0.96)
		Ventana PD-L1 (SP142)	13	0.58 (0.51–0.66)	0.98 (0.96–0.99)
		28-8 LDT	4	See Table B	
		SP142 LDT	1	See Table C	
		73-10 Assay	1	See Table C	
		22C3 LDT	1	See Table C	
50%	PD-L1 IHC 22C3 pharmDx	22C3 LDT	10	See Table B	
		PD-L1 IHC 28-8 pharmDx	21	0.94 (0.88–0.97)	0.95 (0.93–0.97)
		28-8 LDT	6	0.95 (0.81–0.99)	0.76 (0.67–0.83)
		Ventana PD-L1 (SP263)	16	0.92 (0.84–0.96)	0.92 (0.90–0.94)
		E1L3N LDT	13	0.76 (0.62–0.86)	0.97 (0.95–0.99)
		Ventana PD-L1 (SP142)	17	0.42 (0.31–0.54)	1.00 (0.99–1.00)
		SP142 LDT	3	See Table C	
		SP263 LDT	1	See Table C	
		73-10 Assay	1	See Table C	
	Ventana PD-L1 (SP263)	28-8 LDT	4	See Table B	
		PD-L1 IHC 28-8 pharmDx	13	0.69 (0.56–0.79)	0.98 (0.96–0.99)
		PD-L1 IHC 22C3 pharmDx	17	0.57 (0.46–0.68)	0.99 (0.98–1.00)
		E1L3N LDT	9	0.36 (0.28–0.44)	0.99 (0.93–1.00)
		Ventana PD-L1 (SP142)	10	See Table B	
		SP142 LDT	1	See Table C	
		22C3 LDT	1	See Table C	
		73-10 Assay	1	See Table C	

Cochran’s heterogeneity statistic Q and I^2^ for sensitivity and specificity across all studies are shown in Supplementary Files Table 1.

### Non-converging data

Where the number of studies was less than four or when the data were sparse due to the presence of a zero result in contingency tables (e.g., where sensitivity or specificity was 100%), the models did not converge and did not allow for meta-analysis calculations. As summarized in Tables 2 and 3, the latter occurred in a number of studies that had excellent results for both sensitivity and specificity (e.g., 22C3 laboratory developed test compared to PD-L1 IHC 22C3 pharmDx) or specificity only (e.g., Ventana PD-L1 (SP142) compared to Ventana PD-L1 (SP263) and other assays).

**Table 2:** Sensitivity and specificity of individual studies for which meta-analysis was not performed because of non-converging data

TPS Cut-off	Gold Standard Assay	Candidate Assay	Author	Year	Tumor Type*	Sample Size	Sensitivity	Specificity
50%	PD-L1 IHC 22C3 pharmDx	22C3 LDT	Ilie et al.	2018	L	120	1.00	1.00
			Ilie et al	2017	L	120	1.00	1.00
			Røge et al.	2017	L	75	0.93	1.00
			Røge et al.	2017	L	75	1.00	1.00
			Neuman et al.	2016	L	41	1.00	1.00
			Ilie et al.	2018	L	120	1.00	1.00
			Røge et al.	2017	L	75	1.00	1.00
			Ilie et al	2017	L	120	1.00	1.00
			Neuman et al.	2016	L	41	1.00	1.00
			Munari et al.	2018	L	183	0.85	0.97
			Hendry et al.	2018	L	551	0.84	1.00
50%	Ventana PD-L1 (SP263)	Ventana PD-L1 (SP142)	Adam et al.	2018	L	41	0.19	1.00
			Adam et al.	2018	L	41	0.11	1.00
			Adam et al.	2018	L	41	0.19	1.00
			Adam et al.	2018	L	41	0.11	1.00
			Chan et al.	2018	L	713	0.67	1.00
			Fujimoto et al.	2017	L	40	0.33	1.00
			Scheel et al.	2016	L	135	0.54	1.00
			Soo et al.	2018	L	18	0.33	1.00
			Tretiakova et al.	2018	U	161	0.46	0.96
			Kim et al.	2017	L	97	0.00	1.00
			Cheung et al.	2019	L	54	0.60	1.00
			Hendry et al.	2018	L	355	0.33	1.00
			Tsao et al.	2018	L	81	0.28	1.00
1%	Ventana PD-L1 (SP263)	28-8 LDT	Adam et al.	2018	L	41	0.70	1.00
			Adam et al.	2018	L	41	0.73	1.00
			Adam et al.	2018	L	41	0.76	1.00
			Adam et al.	2018	L	41	0.67	0.93
50%	Ventana PD-L1 (SP263)	28-8 LDT	Adam et al.	2018	L	41	1.00	0.81
			Adam et al.	2018	L	41	1.00	0.76
			Adam et al.	2018	L	41	0.82	0.67
			Adam et al.	2018	L	41	1.00	0.70
1%	PD-L1 IHC 22C3 pharmDx	28-8 LDT	Adam et al.	2018	L	41	0.66	1.00
			Adam et al.	2018	L	41	0.63	1.00
			Adam et al.	2018	L	41	0.68	1.00
			Adam et al.	2018	L	32	0.70	1.00
			Adam et al.	2018	L	32	0.67	1.00
			Adam et al.	2018	L	32	0.73	1.00

* *L* NSCLC, *U* UC

**Table 3:** Sensitivity and specificity of individual studies for which meta-analysis was not performed because of insufficient number of studies

TPS Cut-off	Gold Standard Assay	Candidate Assay	Author	Year	Tumour Type	Sample Size	Sensitivity	Specificity
1%	PD-L1 IHC 22C3 pharmDx	SP142 LDT	Sakane et al.	2018	TC	53	1.00 (0.90–1.00)	0.53 (0.32–0.73)
			Soo et al.	2018	NSCLC	18	1.00 (0.77–1.00)	0.00 (0.00–0.43)
			Watanabe et al.	2018	M	32	0.83 (0.61–0.94)	0.86 (0.60–0.96)
		SP263 LDT	Sakane et al.	2018	TC	53	0.97 (0.85–1.0)	0.63 (0.41–0.81)
			Watanabe et al.	2018	M	32	0.78 (0.55-0.91)	0.93 (0.69–0.99)
		73-10 Assay	Tsao et al.	2018	NSCLC	81	1.00 (0.92–1.00)	0.53 (0.37–0.68)
	PD-L1 IHC 28-8 pharmDx	SP142 LDT	Sakane et al.	2018	TC	53	0.95 (0.84–0.99)	0.67 (0.39–0.86)
			Watanabe et al.	2018	M	32	0.82 (0.59–0.94)	0.80 (0.55–0.93)
		SP263 LDT	Sakane et al.	2018	TC	53	0.90 (0.78–0.96)	0.75 (0.47–0.91)
			Watanabe et al.	2018	M	32	0.88 (0.66–0.97)	1.00 (0.80–1.00)
		73-10 Assay	Tsao et al.	2018	NSCLC	81	0.96 (0.88–0.99)	0.63 (0.44–0.79)
	Ventana PD-L1 (SP263)	SP142 LDT	Soo et al.	2018	NSCLC	18	1.00 (0.80–1.00)	0.00 (0.00–0.56)
		73-10 Assay	Tsao et al.	2018	NSCLC	81	0.96 (0.87-0.99)	0.55 (0.38-0.71)
		22C3 LDT	Munari et al.	2018	NSCLC	184	0.66 (0.55–0.76)	0.99 (0.95–1.00)
50%	PD-L1 IHC 22C3 pharmDx	SP142 LDT	Sakane et al.	2018	TC	53	1.00 (0.85–1.00)	0.74 (0.56–0.86)
			Soo et al.	2018	NSCLC	18	1.00 (0.34–1.00)	0.75 (0.51–0.90)
			Watanabe et al.	2018	M	32	0.67 (0.21–0.94)	0.66 (0.47–0.45)
		SP263 LDT	Sakane et al.	2018	TC	53	0.82 (0.62–0.93)	0.97 (0.84–0.99)
			Watanabe et al.	2018	M	32	0.67 (0.21–0.94)	0.86 (0.69–0.70)
		73-10 Assay	Tsao et al.	2018	NSCLC	81	1.00 (0.80-1.00)	0.82 (0.71–0.89)
	Ventana PD-L1 (SP263)	SP142 LDT	Soo et al.	2018	NSCLC	18	1.00 (0.44–1.00)	0.80 (0.55–0.93)
		22C3 LDT	Munari et al.	2018	NSCLC	184	0.64 (0.45–0.80)	0.99 (0.97–1.00)
		73-10 Assay	Tsao et al.	2018	NSCLC	81	0.94 (0.74–0.99)	0.84 (0.73–0.91)

*L* NSCLC, *M* mesothelioma, *U* UC, *TC* Thymic Carcinoma

### PD-L1 IHC 22C3 pharmDx as reference standard

The highest diagnostic accuracy was shown for well-designed 22C3 laboratory developed tests compared to PD-L1 IHC pharmDx 22C3. The sensitivity and specificity were both 100% in 8/9 assays for the 50% tumor proportion score cut-off point (Table 2). The results were almost identical, and only slightly less robust for the 1% cut-off (Fig. 1a, Table 2). Both PD-L1 IHC 28-8 pharmDx and Ventana PD-L1 (SP263) showed acceptable diagnostic accuracy for the 50% cut-off, but both had <90% specificity against the 1% tumor proportion score cut-off (Fig. 1b–e, Table 1).

No other candidate assays reached 90% sensitivity and specificity in the meta-analysis for either the 50% or the 1% tumor proportion score cut-off for PD-L1 IHC 22C3 pharmDx (Table 1 and Table 3). Although the overall performance of E1L3N laboratory developed tests in the meta-analysis was not good, E1L3N laboratory developed tests achieved very high sensitivity and specificity in 3 of 12 comparisons (Fig. 1f–h, Tables 1A and 1B) [18, 27].

### PD-L1 IHC 28-8 pharmDx as reference standard

The highest results were achieved by the Ventana PD-L1 (SP263) assay; it had acceptable accuracy in the meta-analysis compared to PD-L1 IHC pharm Dx 28-8 at the 1% cut-off (6/12 tests were clinically acceptable) (Fig. 1h, Table 1). PD-L1 IHC 22C3 pharmDx did not reach ≥ 90% for both sensitivity and specificity in the meta-analysis when compared to PD-L1 IHC pharmDx 28-8 at the 1% cut-off, although 9/19 individual assay comparisons showed sensitivity and specificity of ≥ 90% (Fig. 1i, Table 1A and 1B).

### Ventana PD-L1 (SP263) as reference standard

No candidate assays achieved required diagnostic accuracy for either the 1% or the 50% cut-off. Most candidate assays achieved acceptable specificity, but the sensitivity was too low for both cut-off points (Tables 1–3).

## Discussion

The most dominant result of this meta-analysis is that properly designed laboratory developed tests that are performed in an individual immunohistochemistry laboratory (usually a reference laboratory or expert-led laboratory) and are developed for the same purpose as the relevant comparative reference method standard may perform essentially equally to the original Food and Drug Administration-approved assay, but also generally better than the Food and Drug Administration-approved companion diagnostics that were originally developed for different purposes. For example, to identify patients with non-small cell lung cancer for second line therapy with pembrolizumab where PD-L1 IHC pharmDx 22C3 is not available, the results of our study indicate that it is more likely that 22C3 or E1L3N well-developed, fit-for-purpose laboratory developed tests would identify the same patients as positive and/or negative as PD-L1 IHC pharmDx 22C3, rather than Ventana PD-L1 (SP263), Ventana PD-L1 (SP142), or PD-L1 IHC pharmDx 28-8, which were developed for different purposes [49–53].

The accuracy of laboratory developed tests varied in our meta-analysis. 22C3 laboratory developed tests achieved the best results, with both sensitivity and specificity of 100% in 8/9 studies. E1L3N also showed excellent results, but in only 3/12 comparisons. Its success in 3 separate comparisons illustrates that it is possible to develop an acceptable laboratory developed test with this clone and that this antibody can be optimized for clinical applications for which the PD-L1 IHC 22C3 pharmDx was developed. The successful applications of some of the laboratory developed tests reinforce the importance of considering the original purpose of the immunohistochemistry assay, a point emphasized in the ISIMM and IQN Path series of papers entitled “Evolution of Quality Assurance for Immunohistochemistry in the Era of Personalized Medicine” [54–57]. It should be pointed out that our meta-analysis indicates that excellent diagnostic accuracy by laboratory developed tests can be achieved in some laboratories where the laboratory developed tests that were included in this study were originally developed; it remains to be determined whether the same laboratory developed tests would perform the same if more widely tested in different laboratories with different operators using different equipment. External quality assurance including inter-laboratory comparisons, as well as proficiency testing demonstrated that as high as 20–30% or more of the participating laboratories may produce poor results with immunohistochemistry laboratory developed test protocols [58–62]. The success of laboratory developed tests depends on multiple parameters, including which test performance characteristics and which tissue tools may have been used for test development and validation [56, 57]. In the case of predictive PD-L1 immunohistochemistry assays, recognition and careful definition of the assay purpose according to the 3D approach (Disease, Drug, Diagnostic assay) must also be considered, along with proper selection of the comparative method for determination of diagnostic accuracy of the newly developed candidate test. Several studies have demonstrated that when laboratories follow this approach, they are able to produce excellent results [24, 32, 36–38]. Our study and previously published results do not imply generalizable analytical robustness of laboratory developed tests, whether de novo laboratory developed tests or “kit-derived laboratory developed tests” [6, 32]. When protocols for laboratory developed tests are shared between laboratories, it is essential that the adopting laboratory conducts initial technical validation, which would increase the likelihood of similar diagnostic accuracy [48, 56]. However, the purpose of predictive PD-L1 immunohistochemistry assays is not to demonstrate the best signal-to-noise ratio (“nice” and highly sensitive results), but to identify patients that are more likely to benefit from specific drug(s) as demonstrated in clinical trials. Therefore, consideration of this purpose and direct or indirect link with the clinical trial results is always required and it should be considered in test development, test validation, test maintenance, as well as in test performance comparison.

As so far there are no tools to measure analytical sensitivity and specificity of immunohistochemistry assays; this presents a significant problem in assay development, methodology transfer, and daily monitoring of assay performance, as well as direct comparison of assay calibration. The lack of tools that could assess analytical sensitivity and specificity also hinders attempts of immunohistochemistry protocol standardization/harmonization for the PD-L1 assays; without such tools it is not possible to determine the desirable range of analytical sensitivity and specificity of relevance for diagnostic accuracy for any of the PD-L1 assays. This is one cause that we can identify as a potential source of the discrepancy between previously published works that suggested analytical interchangeability of the several Food and Drug Administration-approved PD-L1 assays, but did not necessarily lead to interchangeability based on calculated diagnostic accuracy as shown in our study.

The Ventana PD-L1 (SP263) assay had very high diagnostic sensitivity against all other Food and Drug Administration-approved PD-L1 assays, but its diagnostic specificity was consequently lower. Although several of the studies included in this meta-analysis demonstrated substantial analytical similarity between PD-L1 IHC 22C3 pharmDx, PD-L1 IHC 28-8 pharmDx, and Ventana PD-L1 (SP263), our cumulative results suggest that the diagnostic sensitivity of these various assays (and indirectly their analytical sensitivity) is ordered as follows: PD-L1 IHC 22C3 pharmDx < PD-L1 IHC 28-8 pharmDx < Ventana PD-L1 (SP263).

The results of this meta-analysis confirm previous observations that the Ventana PD-L1 (SP142) assay’s analytical sensitivity is significantly lower than that of the three other Food and Drug Administration-approved PD-L1 assays and that the diagnostic sensitivity of Ventana PD-L1 (SP142) against PD-L1 IHC 22C3 pharmDx, PD-L1 IHC 28-8 pharmDx, and Ventana PD-L1 (SP263) assays is prohibitively low for both the 1% and the 50% tumor proportion score in non-small cell lung cancer and other tumor models.

Several investigators have evaluated the so-called “interchangeability” of PD-L1 immunohistochemistry assays. The term “interchangeability” has also been used widely by the pharmacological industry to designate drugs that have demonstrated the following characteristics: same amount of the same active ingredients, comparable pharmacokinetics, same clinically significant formulation characteristics, and to be administered in the same way as the drug prescribed [63]. Basically, interchangeable drugs have the same safety profile and therapeutic effectiveness, as demonstrated in clinical trials [64, 65]. To apply this term to an immunohistochemistry predictive assay, the manufacturer of the assay, be it industry for a companion/complementary diagnostic or a clinical immunohistochemistry laboratory for an laboratory developed test, would need to prove that the alternative assay will produce the same clinical outcomes. Since none of the assay comparisons were performed in the setting of a prospective clinical trial, this type of evidence is not available for PD-L1 immunohistochemistry assays and therefore, none can be deemed “interchangeable” with another in this same sense of the word. In addition, candidate assays and comparative assays cannot interchange their positions for the purpose of calculations without consequences [11]. If “interchangeability” would be defined as achieving ≥90% sensitivity and specificity for both the 1% and the 50% tumor proportion score cut-off points, none of the studies in this meta-analysis demonstrated “interchangeability” of the Food and Drug Administration-approved assays PD-L1 IHC 22C3 pharmDx, PD-L1 IHC 28-8 pharmDx, Ventana PD-L1 (SP142), or Ventana PD-L1 (SP263) for each other.

Although they cannot be designated as “interchangeable”, the diagnostic accuracy of assays for a specific clinical purpose may be compared. In this manner, the comparison indirectly generates results that can be used to justify clinical usage of assays other than those included in the clinical trials. We employed ≥90% diagnostic sensitivity and ≥90% diagnostic specificity because these values are often used in other settings, including performance of immunohistochemistry assays [66–68]. While it is reasonable that a candidate assay should have at least 90% diagnostic sensitivity, it is unclear whether the required diagnostic specificity should be at the same level, or whether lower specificity could also be clinically acceptable. From the perspective of patient safety, lower diagnostic specificity could potentially be acceptable for those indications/purposes where clinical trials demonstrated that progression free survival, overall survival, and adverse effects in patients with PD-L1-negative tumors treated by immunotherapy are at least comparable if not better to that of conventional chemotherapy.

The strengths of this meta-analysis are the focus on diagnostic accuracy, fit-for-purpose approach, and the access to previously unpublished data from a large number of studies, which all resulted in pooled PD-L1 assay comparison in a way that has not been done before.

The most significant limitation is that this is a meta-analysis of test comparisons where designated reference standards are other tests rather than clinical outcomes. However, to complete a meta-analysis with clinical outcomes may not be possible for many years, if ever. Other limitations of this meta-analysis are that only two cut-off points were assessed (1% and 50%), no assessment for readout that includes inflammatory cells was included, the impact of pathologists’ readout as potential source of variation between the studies was not assessed, and it is somewhat uncertain how the results apply to tumors other than non-small cell lung cancer due to the smaller number of such studies.

## Conclusions

The complexity of the PD-L1 immunohistochemistry testing cannot be safely simplified without consideration of the original test purpose. Determination of the diagnostic accuracy and indirect clinical validation of a candidate assay can be achieved by comparing the results of that assay to a previously designated reference standard assay, when direct access to clinical trial data or clinical outcomes is not possible.

### Our meta-analysis indicates that

1) Well-designed, fit-for-purpose PD-L1 laboratory developed test candidate assays may achieve higher accuracy than PD-L1 Food and Drug Administration-approved kits that were designed and approved for a different purpose, when both are compared to an appropriate designated reference standard;

2) More candidate assays achieved ≥ 90% sensitivity and specificity for 50% tumor proportion score cut-off than for 1% tumor proportion score cut-off;

3) The overall diagnostic sensitivity and specificity analyses indicates that the relative analytical sensitivities of the Food and Drug Administration-approved kits for tumor cell scoring, most specifically in non-small cell lung cancer, are as follows: Ventana PD-L1 (SP142) << PD-L1 IHC 22C3 pharmDx < PD-L1 IHC 28-8 pharmDx < Ventana PD-L1 (SP263).

## Supplementary information

Conflict of Interest (Appendix A)

Meta-Analysis Methodology

Supplementary Files Table 1

Supplemetary Figures 2 - 13

Funnel Plots
