# Supplementary material for: “Interchangeability” of PD-L1 immunohistochemistry assays: a meta-analysis of diagnostic accuracy
Source: Mod Pathol. 2019 Aug 5;33(1):4–17. doi: 10.1038/s41379-019-0327-4 (PMC6927905; doi:10.1038/s41379-019-0327-4)
Supplement: Supplementary file 1 — Conflict of Interest (Appendix A) [file 41379_2019_327_MOESM1_ESM.pdf]

I declare potential conflict of interest (enter "none" for negative reply or company name(s) for positive reply) for the period of 2013 - Present:

Last Name, First Name	Board membership or consultancy	Employment	Expert testimony	Grants/grants pending	Payments for lectures with educational/scientific content	Payments for speakers bureau	Payment for manuscript preparation	Patents (planned, pending, issued)	Royalties	Stock/stock options	Other (travel/ accommodations/ meeting expenses not related to any of the above)	Other (err on the side of full disclosure)
Adam, Julien	AstraZeneca, Bayer,BMS, MSD, Roche	none	none	none	none	none	none	none	none	none	none	none
Barnes, Penny	none	none	none	none	none	none	none	none	none	none	none	none
Bigras, Gilbert	Merck, Pfizer, Astrazeneca, BMS, Roche	none	none	Merck and Roche	none	none	none	none	none	none	none	none
Chan, Anthony W.H.	none	none	none	none	none	none	none	none	none	none	none	none
Chao Li	none	none	none	none	none	none	none	none	none	none	none	none
Cheung, Carol	Agilent, Astra-Zeneca, Bristol-Myers-Squibb, Cell Marque/Sigma, Merck, Roche	none	none	Roche	Agilent, Astra-Zeneca, Bristol-Myers-Squibb.	none	none	none	none	none	none	none
Chung, Jin-Haeng	none	none	none	none	none	none	none	none	none	none	none	none
Couture, Christian	Merck, Pfizer, Astra Zeneca, Boehringer-Ingelheim, Bristol-Meyer-Squibb, AbbVie, Roche, Qiagen	none	none	Merck	Merck, Pfizer, Novartis	none	none	none	none	none	none	none
Fiset, Pierre Olivier	Consultancy for AstraZeneca Canada, Pfizer Canada and Merck Canada	none	none	none	none	none	none	none	none	none	none	none
Fujimoto, Daichi	none	none	none	Astrazeneca KK	AstraZeneca KK, Ono Pharmaceutical Co Ltd, Bristol-Myers Squibb Co Ltd, Taiho Pharmaceutical Co Ltd, Chugai Pharmaceutical Co Ltd, MSD KK, Boehringer Ingelheim Japan Inc, Eli Lilly Japan KK,	none	none	none	none	none	none	none
Gilbert, Bigras	Merck, Pfizer, Roche, Bayer, Bristol-Myers Squibb	none	none	Merck, Roche	none	Merck, Roche	none	none	none	Merck, Roche	none	none
Han, Gang	none	none	none	none	none	none	none	none	none	none	none	none
Hirsch, Fred R.	Scientific advisory boards: BMS, AstraZeneca, Merck, Lilly, Novartis, Ventana, Genentech/Roche,Helsinn, Loxo, Bayer, HTG molecular, Biocept	none	none	BMS, Genenetech, Ventana, Bayer, Amgen, Clovis, Biodesix (all through University of Colorado to my laboratory).	AstraZeneca	none	none	"EGFR FISH and IHC as predictive biomarkers for EGFR therapy"	none	none	none	none
Ilie, Marius	none	none	none	Roche, Boehringer-Ingelheim	Roche, Boehringer-Ingelheim, BMS, AstraZeneca	none	none	none	none	none	none	none
Ionescu, Diana N	ASTRA-ZENECA, PFIZER, ROCHE, ELI-LILLY, NOVARTIS, BMS, MERCK	none	none	ASTRA-ZENECA	ASTRA-ZENECA, PFIZER, ROCHE, ELI-LILLY, NOVARTIS, BMS, MERCK	none	none	none	none	none	PFIZER	none
Katsuhiro Okuda	none	none	none	none	none	none	none	none	none	none	none	none
Lim, Hyun J.	none	none	none	none	none	none	none	none	none	none	none	none
Munari, Enrico	none	none	none	none	none	none	none	none	none	none	none	none
Ratcliffe, Marianne	none	AstraZeneca	none	none	none	none	none	none	none	none	none	none
Rimm, David	Amgen, Astra Zeneca, Agendia, Biocept, BMS, Cell Signaling Technology, Cepheid, Daiichi Sankyo, GSK, InVicro/Konica Minolta, Merck, NanoString, Perkin Elmer, PAIGE.AI, and Ultivue	none	none	Astra Zeneca, Cepheid, Navigate/ Novartis, NextCure, Lilly, Ultivue, Ventana and Perkin Elmer/Akoya	Ventana	none	none	AQUA, owned by Yale, CTC licensed to Rarecyte	Rarecyte	Pixelgear	none	none
Røge, Rasmus	none	none	none	none	none	none	none	none	none	none	none	none
Ross,Catherine	none	none	none	none	Alexion, Celgene, Roche, Janssen, Novartis	none	none	none	none	none	none	none
Scheel, Andreas H.	NordiQC; QuIP	none	BMS, MSD, Roche Pharma, AstraZenec a	none	BMS, MSD	none	none	none	none	none	none	none
Soo, Ross	none	none	none	none	Astra Zeneca, BMS, Boehringer Ingelheim, Celgene, Ignyta, Lilly, Merck, Novartis, Pfizer, Roche, Taiho, Takeda, Yuhan	none	none	none	none	none	none	none
Swanson, Paul	Astra-Zeneca Canada	none	none	none	Pacific Northwest Society of Surgical Pathologists spring 2018 meeting	none	none	none	none	none	none	none
To, Ka Fai	none	none	none	none	none	none	none	none	none	none	none	none
Torlakovic, Emina	Roche, Merck, Pfizer, BMS, Janssen, AstraZeneca	none	none	Roche, Merck, Pfizer, BMS, Janssen, AstraZeneca	Roche, Merck, Pfizer, BMS, Janssen, AstraZeneca	none	none	none	none	none	none	none
Tretiakova, Maria	BMS consultant	none	none	none	none	none	none	none	none	none	none	none
Tsao, Ming	Merck, AstraZeneca, BMS, Ventana/Roche	none	none	Merck	Merck, AstraZeneca	none	none	none	none	none	none	none
Vainer, Gilad	none	none	none	MSD, Roche, Pfizer, Astra-zeneca, Abbvie	MSD, Roche, Pfizer, Astra-zeneca, Abbvie	none	none	none	none	none	MSD, Roche, Abbvie	none
Wang, Hangjun	Pfizer, BMS, Merck, AstraZeneca	none	none	none	none	none	none	none	none	none	none	none
Xu, Zhaolin	Merck Canada National Lung Cancer Medical Advisory Board	none	none	none	none	none	none	none	none	none	none	none
Zielinski, Dirk	none	none	none	none	none	none	none	none	none	none	none	none
