# Supplementary material for: “Interchangeability” of PD-L1 immunohistochemistry assays: a meta-analysis of diagnostic accuracy
Source: Mod Pathol. 2019 Aug 5;33(1):4–17. doi: 10.1038/s41379-019-0327-4 (PMC6927905; doi:10.1038/s41379-019-0327-4)
Supplement: Supplementary file 2 — Meta-Analysis Methodology [file 41379_2019_327_MOESM2_ESM.pdf]

Meta-Analysis Methodology

Panel Composition

The Panel included all current members of the Canadian Association of Pathologists – Association canadienne des pathologistes (CAP-ACP) National Standards Committee for High Complexity Testing (CAP-ACP NSCHCT). Additionally, the Committee invited national and international experts in the field as external consultants. The Steering Committee was formed in order to develop the scope of the Guidelines as well as the key questions.

Meta-analysis of PD-L1 assay interchangeability is a part of Key Question 1 of the CAP-ACP guidelines for PD-L1 assay selection, reporting, and quality assurance (1). Corresponding authors of the publications included in this meta-analysis were contacted to contribute additional data (as per below) and/or contribute to manuscript drafting and conclusions. This resulted in one or two co-authors per included publication depending on the scope of contribution to the meta-analysis.

1. Cheung et al. Fit-For-Purpose PD-L1 Biomarker Testing for Patient Selection in Immuno-Oncology: Guidelines for Clinical Laboratories from The Canadian Association of Pathologists - Association canadienne des pathologistes (CAP-ACP). *In Submission*

Conflict of Interest (COI) Policy

All co-authors declared potential COI for the period 01/2013 - Present including following categories:

- board membership or consultancy
- employment
- expert testimony

- grants/grants pending
- payments for lectures with educational/scientific content
- payment of speakers bureau
- payment for manuscript preparation
- patents (planned, pending, issued)
- royalties
- stock/stock options
- other (travel/accommodations/meeting expenses not related to any of the above)
- other (err on the side of full disclosure)

They also needed to reply separately whether there are other relationships of activities that readers could perceive to have influenced, or that give the appearance of potentially influencing, work on the CAP-ACP PD-L1 meta-analysis of assay interchangeability. Declared potential COI is presented in Supplementary Files Appendix A.

Key Question

Are PD-L1 IHC assays designed/developed as predictive biomarkers for immunotherapy "interchangeable" based on fit-for-purpose approach?

Literature Review

Systematic review of literature was conducted as a part of a national project for developing Canadian guidelines for PD-L1 testing. The Canadian Association of Pathologists – Association canadienne des pathologistes (CAP-ACP) National Standards Committee for High Complexity Testing (CAP-ACP NSCHCT) initiated development of

CAP-ACP Guidelines for PD-L1 testing in order to facilitate introduction of PD-L1 testing for various purposes to Canadian clinical IHC laboratories. The meta-analysis is partly the result of systematic review that was performed for the key questions of CAP-ACP Guidelines for PD-L1 testing.

Systematic Evidence Review (SER) for Meta-Analysis

The objective of the SER was to gather cumulative evidence on interchangeability of various available PD-L1 IHC predictive biomarkers in order to help develop PD-L1 testing guidelines for pathologists and clinical immunohistochemistry laboratories in Canada. The objective of the meta-analysis is to disclose current state of evidence and available strategies relevant to the selection of fit-for-purpose predictive PD-L1 assay/biomarker when Health Canada-approved immunotherapy includes PD-L1 IHC assay as a biomarker in the label of the approved drug.

Search and Selection

A search for literature was performed in MEDLINE using the PubMed interface. Last search was performed on August 31st, 2018. Search strategy using keyword "PD-L1" only was performed for the period of 01/2015 to 08/2016 in order to exclude the possibility of unintentional exclusion of articles based on mismatch of any more specific search terms. Search limits included: "human", and "English". This revealed 2,515 articles, which were downloaded to Zotero reference manager, for which abstracts were reviewed to exclude review papers, case reports, editorials, letters to editor, and any other low level of evidence publication. Furthermore, various

additional targeted searching strategies within the remaining publications including searches for “assay”, “comparison”, “interchangeable”, “comparative”, “optimization”, “validation”, “platforms”, “correlation”, and “agreement”. Two eligibility criteria had to be fulfilled: 1. The study included at least one PD-L1 assay that could be considered as reference standard (companion diagnostic or complementary diagnostic assay), and 2. The study either published 2 x 2 contingency tables for 1% and 50% cut-off point for Tumor Proportion Score (TPS) or the study design produced data that could be sorted in such 2 x 2 contingency tables upon request. 57 publications were selected for full text review. Flow diagram according to Preferred Reporting Items for Systematic Reviews and Meta-Analyses (PRISMA) is provided in Supplementary Files Figure 1. Additional systematic or targeted searches of other databases (e.g. Google Scholar) did not reveal any additional publications.

Review Process

All reviewers received Instructions for review. The instructions detailed methodology and criteria for grading published evidence (See Appendix B for full text of Instructions for Reviewers). Each publication was reviewed by at least two expert reviewers, a statistician, and also during the audit by a methodologist. Any disagreement in the grading between the reviewers was reconciled by additional input from the statistician and/or methodologist, as applicable.

Search Data Management

A bibliographic database was established in Zotero in order to select and track all publications. Two expert panel members reviewed all titles and abstracts identified by the initial search strategy and selected articles for full review using eligibility criteria as defined above (See “Search and Selection”).

Data Abstraction

Data abstraction was performed by expert reviewers who submitted the reviews through specially designed questionnaire on Survey Monkey. Reviewers had to answer twenty-nine questions for each publication that related to Key Question. Also, 22 questions were separately set for abstraction of data relevant to the role of “fit-for-purpose” approach/3D evidence from clinical trials. Additional eleven questions for each publication related to statistical methods were also answered by a statistician and a methodologist. All data abstractions were audited by a methodologist. See Appendix C for list of questions used for data abstraction.

Assessment of Quality of Evidence

Expert reviewers extracted data and assessed quality of evidence by using specially designed Survey Monkey questionnaire that followed published guidelines for the assessment of quality of evidence. Detailed instructions were provided to reviewers in order to employ the same criteria between different reviewers and different publications (see Supplementary Files Appendix B).

Results from Assessment of Quality of Evidence

Quality of the evidence for assay selection/interchangeability was summarized in two Evidence Tables ("Interchangeability" and "Statistical"). Most published test comparisons focused on analytical sensitivity rather than on diagnostic accuracy of the PD-L1 IHC biomarkers for specific purpose/application.

Most published studies did not include 2 x 2 tables that would allow calculations of diagnostic sensitivity and specificity or positive percent agreement (PPA) and negative percent agreement (NPA). Some published studies calculated PPA and NPA, but did not include 2 x 2 contingency tables with data or included tables with various different cut-off points. Finally, only rare published manuscripts included 2 x 2 contingency tables with results that already specifically addressed diagnostic sensitivity and specificity (or percent positive agreement and percent negative agreement).

The CAP-ACP National Standards Committee for High Complexity Testing (CAP-ACP NSCHCT) contacted the authors of studies that were presumed to have generated data that could be presented in 2 x 2 contingency tables to enable calculations for different cut-off points, but this data was not included in their published manuscript. Most studies required multiple 2 x 2 tables as each one was designed for specific purpose and set of assays. This request resulted in cumulative evidence of 376 test comparisons for different purposes, which were included in meta-analysis that focused on the assay sensitivity and specificity for identified specific clinical application/purpose. Therefore, the number of studies comparing different assays (376) in this meta-analysis was much larger than the number of published manuscripts (22) due to frequent inclusion of multiple test

comparisons and this combined with the consideration of different readout cut-off points for “positive” vs. “negative” result.

Relevance of Purpose-Based Approach

Clinical trial publications were the source of evidence for selection of “designated reference standard” predictive PD-L1 assay. The various clinical applications/purposes were identified either as specifically identified/listed in the published study or based on the inclusion of a specific CDX assays for which the purpose has been established. Although large number of potential purposes were identified, only five were included in this meta-analysis; the selection was principally based on the type of data available including which IHC protocols were used and which readout was performed by the authors. The greatest limitation in the published studies was based on the selection of the readout employed to assess the results. Published literature most frequently compared performance of PD-L1 IHC assays for the following readouts: 1% and 50% tumour proportion score (TPS). Hence, these two readouts and three different FDA-approved kits (reference standards) were selected for comparing tests’ sensitivity and specificity.

Relevance of Tissue Model

Most studies used non-small cell lung carcinoma (NSCLC) as tissue model for test comparisons (337 test comparisons). Other tissue models also included urothelial carcinoma (20 test comparisons), mesothelioma (9 test comparisons), and thymoma (nine test comparisons). Tissue models included in the meta-analysis were limited to only those studies that included at least one PD-L1 assay that is recognized gold standard for at least

one purpose according to the 3D approach. Therefore, some tissue models could not be included (e.g. melanoma, breast cancer). The number of cases included in each study is shown in manuscript tables (Tables 1A, 1B, 2, and 3).
