# Supplementary material for: “Interchangeability” of PD-L1 immunohistochemistry assays: a meta-analysis of diagnostic accuracy
Source: Mod Pathol. 2019 Aug 5;33(1):4–17. doi: 10.1038/s41379-019-0327-4 (PMC6927905; doi:10.1038/s41379-019-0327-4)
Supplement: Supplementary file 3 — Supplementary Files Table 1 [file 41379_2019_327_MOESM3_ESM.pdf]

Supplementary Files Table 1. Cochran's heterogeneity statistic Q and I^2 for sensitivity and specificity across all studies

Group	Candidate Assay	Comparative Assay	TPS Cut-off	Sensitivity Q statistic, p-value	Sensitivity I^2	Specificity Q statistic, p-value	Specificity I^2
1	PD-L1 IHC pharmDx 28-8	PD-L1 IHC pharmDx 22C3	50%	Q=71.8, p <0.0001	732.2	Q=92.2, p <0.0001	78.3
2	PD-L1 IHC pharmDx 28-8	PD-L1 IHC pharmDx 22C3	1%	Q=109.1, p <0.0001	81.7	Q=132.5, p <0.0001	84.9
3	Ventana PD-L1 (SP263)	PD-L1 IHC pharmDx 22C3	50%	Q=45.8, p <0.0001	67.3	Q=48.3, p <0.0001	68.9
4	Ventana PD-L1 (SP263)	PD-L1 IHC pharmDx 22C3	1%	Q=43.4, p < 0.0001	65.4	Q=34.6, p <0.0001	56.7
5	Ventana PD-L1 (SP142)	PD-L1 IHC pharmDx 22C3	50%	Q=88.9, p <0.0001	82.0	**Q=24.9, p = 0.07**	**35.8**
6	Ventana PD-L1 (SP142)	PD-L1 IHC pharmDx 22C3	1%	Q=150.3, p <0.0001	88.7	Q=208.8, p <0.0001	91.9
7	73-10 Assay	PD-L1 IHC pharmDx 22C3	50%	NA	NA	NA	NA
8	73-10 Assay	PD-L1 IHC pharmDx 22C3	1%	NA	NA	NA	NA
9	22C3 LDT	PD-L1 IHC pharmDx 22C3	50%	NA	NA	NA	NA
10	22C3 LDT	PD-L1 IHC pharmDx 22C3	1%	Q=380.9, p <0.0001	97.4	Q=930.0, p <0.0001	98.9
11	E1L3N LDT	PD-L1 IHC pharmDx 22C3	50%	Q=25.0, p = 0.01	52.0	**Q=13.6, p = 0.32**	12.0
12	E1L3N LDT	PD-L1 IHC pharmDx 22C3	1%	Q=25.2, p = 0.02	48.5	Q=44.1, p < 0.0001	70.5
13	PD-L1 IHC pharmDx 22C3	PD-L1 IHC pharmDx 28-8	1%	Q=114.6, p <0.0001	79.9	Q=117.3, p <0.0001	80.4
14	Ventana PD-L1 (SP263)	PD-L1 IHC pharmDx 28-8	1%	Q=44.7, p <0.0001	70.9	Q=94.0, p <0.0001	80.3
15	Ventana PD-L1 (SP142)	PD-L1 IHC pharmDx 28-8	1%	Q=78.3, p <0.0001	84.7	**Q=19.3, p = 0.08**	**37.7**
16	73-10 Assay	PD-L1 IHC pharmDx 28-8	1%	NA	NA	NA	NA
17	E1L3N LDT	PD-L1 IHC pharmDx 28-8	1%	**Q=9.1, p = 0.62**	**0.0**	Q=47.6, p <0.0001	76.9
18	PD-L1 IHC pharmDx 22C3	Ventana PD-L1 (SP263)	50%	Q=127.4, p <0.0001	87.4	Q=29.2, p = 0.02	45.2
19	PD-L1 IHC pharmDx 22C3	Ventana PD-L1 (SP263)	1%	Q=143.0, p <0.0001	88.8	Q=109.5, p <0.0001	85.4
20	PD-L1 IHC pharmDx 28-8	Ventana PD-L1 (SP263)	50%	Q=115.6, p <0.0001	89.6	**Q=15.4, p = 0.22**	**22.1**
21	PD-L1 IHC pharmDx 28-8	Ventana PD-L1 (SP263)	1%	Q=169.9, p <0.0001	92.4	Q=48.4, p <0.0001	73.2
22	Ventana PD-L1 (SP142)	Ventana PD-L1 (SP263)	50%	NA	NA	NA	NA
23	Ventana PD-L1 (SP142)	Ventana PD-L1 (SP263)	1%	Q=63.0, p <0.0001	81.0	Q=29.1, p < 0.0001	58.8
24	73-10 Assay	Ventana PD-L1	50%	NA	NA	NA	NA

		(SP263)					
25	73-10 Assay	Ventana PD-L1 (SP263)	1%	NA	NA	NA	NA
26	22C3 LDT	Ventana PD-L1 (SP263)	50%	NA	NA	NA	NA
27	22C3 LDT	Ventana PD-L1 (SP263)	1%	NA	NA	NA	NA
28	E1L3N LDT	Ventana PD-L1 (SP263)	50%	**Q=8.2, p = 0.42**	**2.3**	**Q=5.9, p = 0.66**	**0.0**
29	E1L3N LDT	Ventana PD-L1 (SP263)	1%	**Q=13.5, p = 0.10**	**40.6**	**Q=10.2, p = 0.25**	**21.3**
32	28-8 LDT	Ventana PD-L1 (SP263)	1%	NA	NA	NA	NA
33	28-8 LDT	Ventana PD-L1 (SP263)	50%	NA	NA	NA	NA
34	SP142 LDT	PD-L1 IHC pharmDx 22C3	1%	NA	NA	NA	NA
35	SP142 LDT	PD-L1 IHC pharmDx 22C3	50%	NA	NA	NA	NA
36	SP142 LDT	Ventana PD-L1 (SP263)	1%	NA	NA	NA	NA
37	SP142 LDT	Ventana PD-L1 (SP263)	50%	NA	NA	NA	NA
38	SP263 LDT	PD-L1 IHC pharmDx 22C3	1%	NA	NA	NA	NA
39	SP263 LDT	PD-L1 IHC pharmDx 22C3	50%	NA	NA	NA	NA
40	SP142 LDT	PD-L1 IHC pharmDx 28-8	1%	NA	NA	NA	NA
41	SP263 LDT	PD-L1 IHC pharmDx 28-8	1%	NA	NA	NA	NA
42	28-8 LDT	PD-L1 IHC pharmDx 28-8	1%	**Q=1.73, p = 0.63**	**0.0**	**Q=1.6, p = 0.67**	**0.0**
43	28-8 LDT	PD-L1 IHC pharmDx 22C3	50%	**Q=6.8, p = 0.24**	**26.4**	**Q=5.4, p = 0.37**	**6.8**
44	28-8 LDT	PD-L1 IHC pharmDx 22C3	1%	NA	NA	NA	NA

NA: meta-analysis is not available because of small number of studies. Bold font indicates 'statistically not heterogeneous' among studies.
