# Supplementary material for: “Interchangeability” of PD-L1 immunohistochemistry assays: a meta-analysis of diagnostic accuracy
Source: Mod Pathol. 2019 Aug 5;33(1):4–17. doi: 10.1038/s41379-019-0327-4 (PMC6927905; doi:10.1038/s41379-019-0327-4)

Figure 2 Ventana PD-L1 (SP142) (candidate) vs. PD-L1 IHC pharmDx 22C3 (RS) for 50% TPS Cut-off

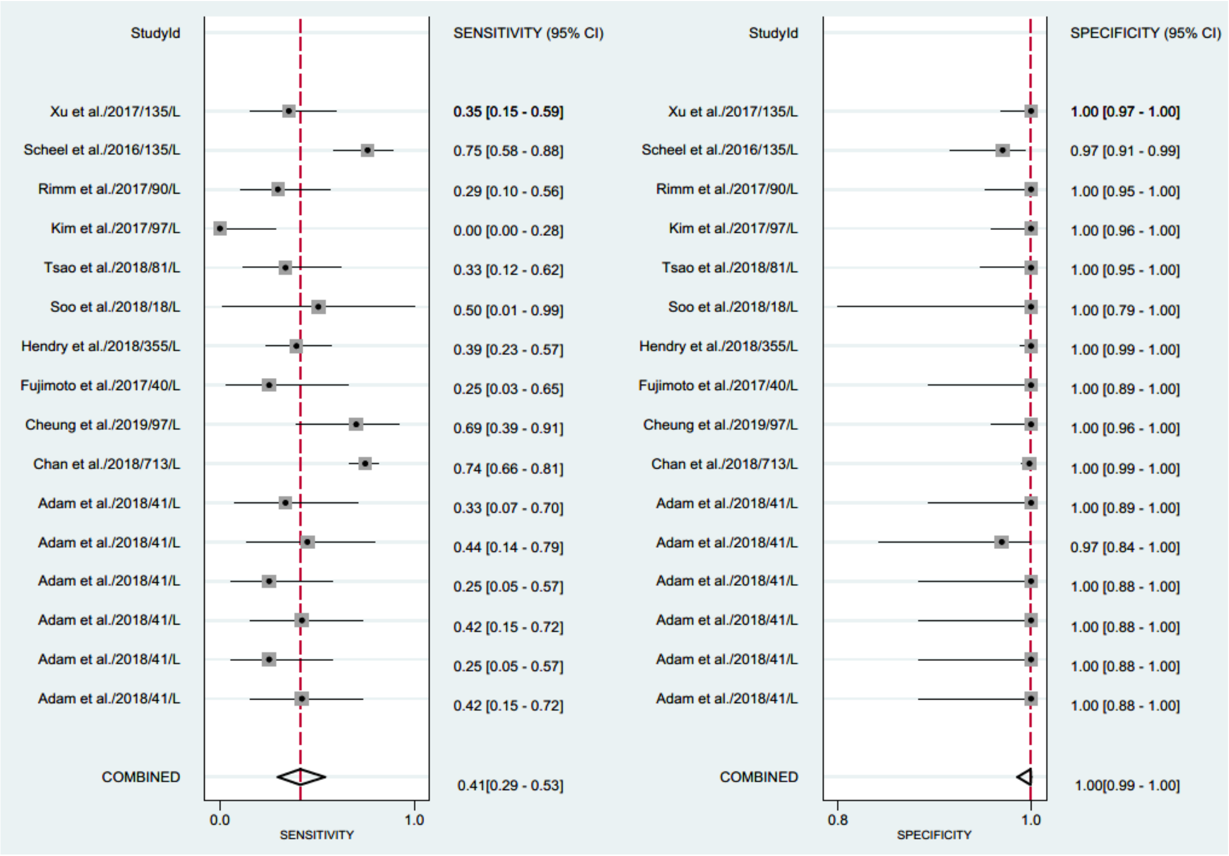

Figure 3 Ventana PD-L1 (SP142) (candidate) vs. PD-L1 IHC pharmDx 22C3 (RS) for 1% TPS Cut-off

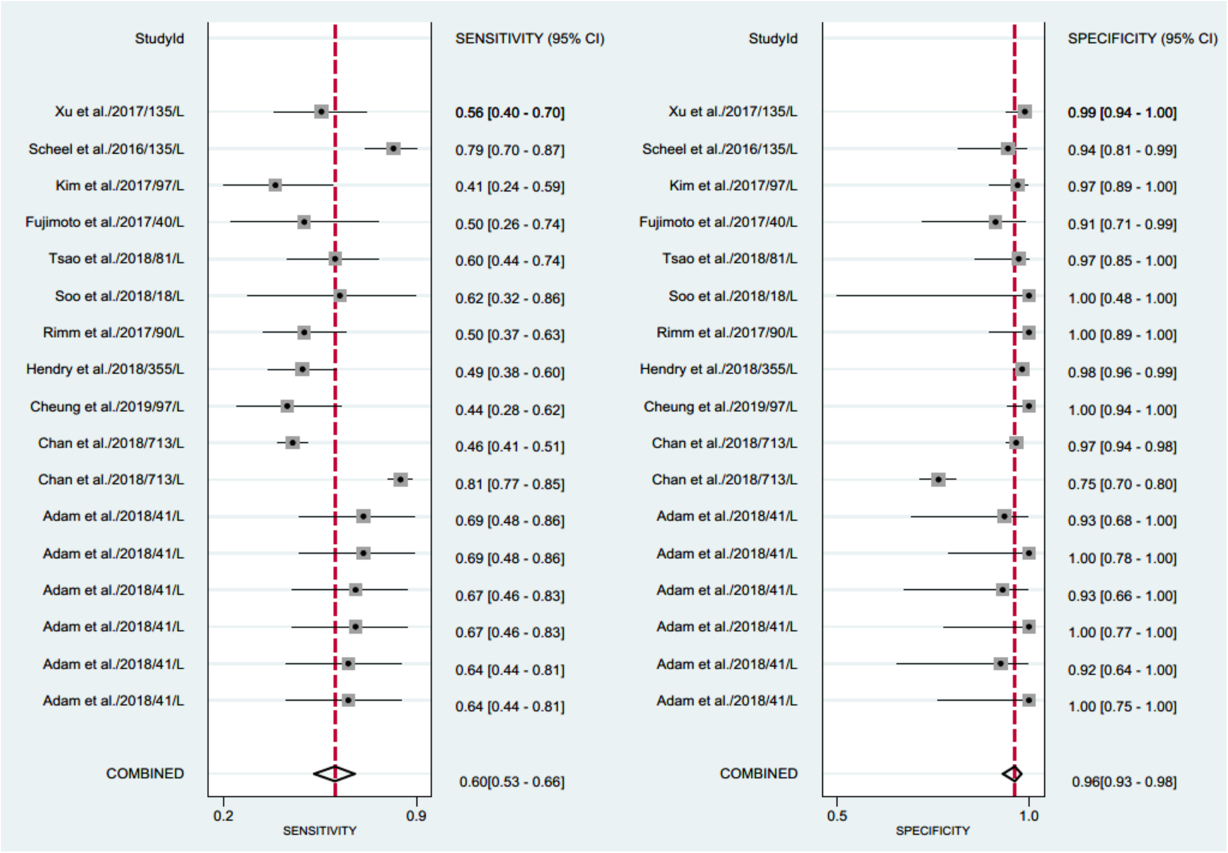

Figure 4 Ventana PD-L1 (SP142) (candidate) vs. PD-L1 IHC pharmDx 22C3 (RS) for 1% TPS Cut-off

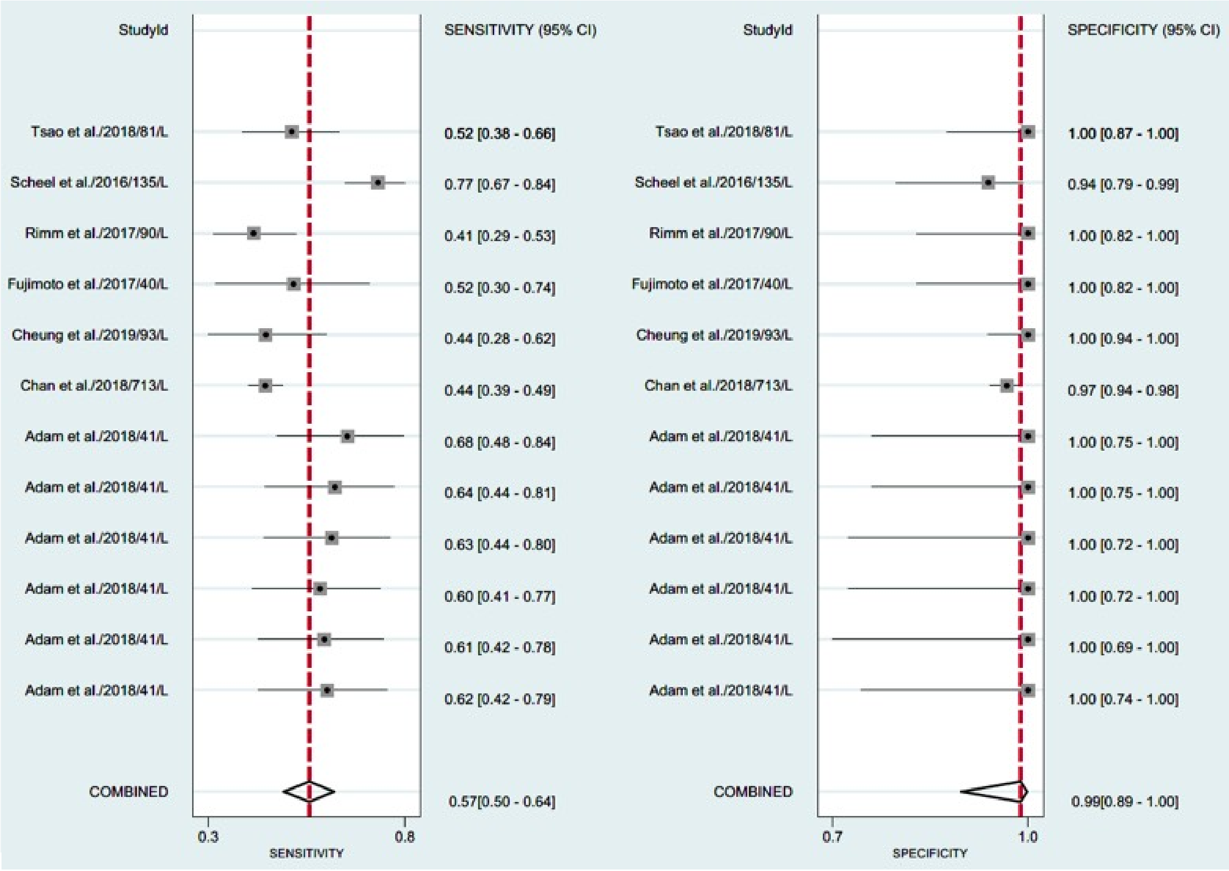

Figure 5 E1L3N LDT (candidate) vs. PD-L1 IHC pharmDx 28-8 (RS) for 1% TPS Cut-off

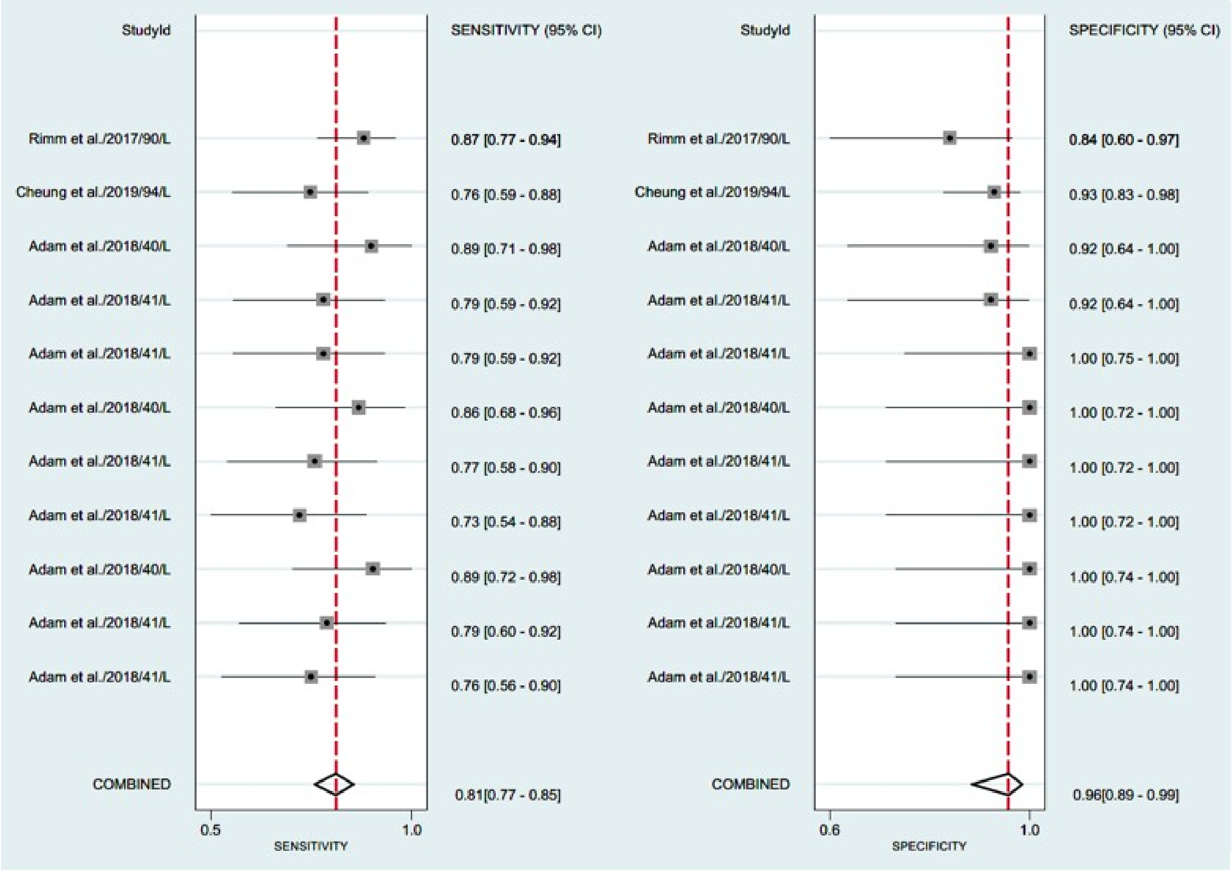

Figure 6 PD-L1 IHC pharmDx 22C3 (candidate) vs. Ventana PD-L1 (SP263) (RS) for 50% TPS Cut-off

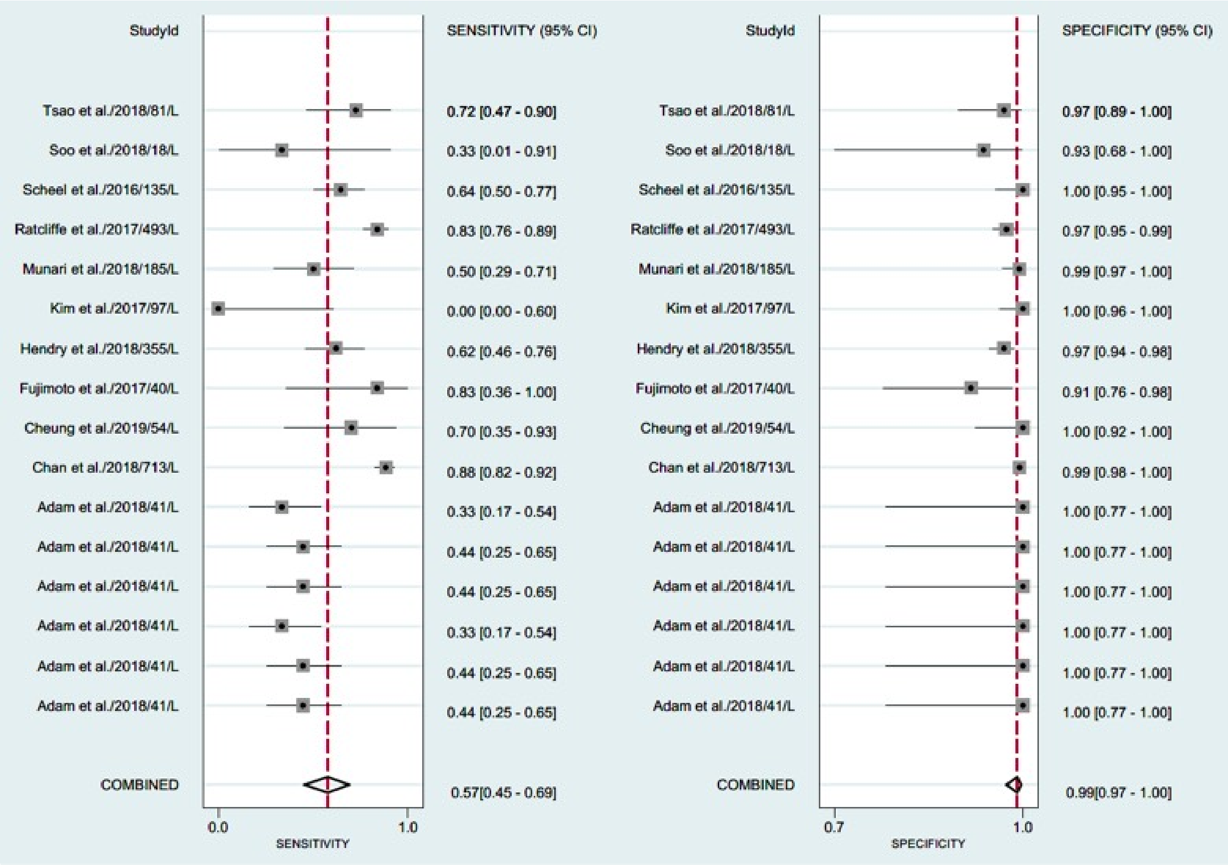

Figure 7 PD-L1 IHC pharmDx 22C3 (candidate) vs. Ventana PD-L1 (SP263) (RS) for 1% TPS Cut-off

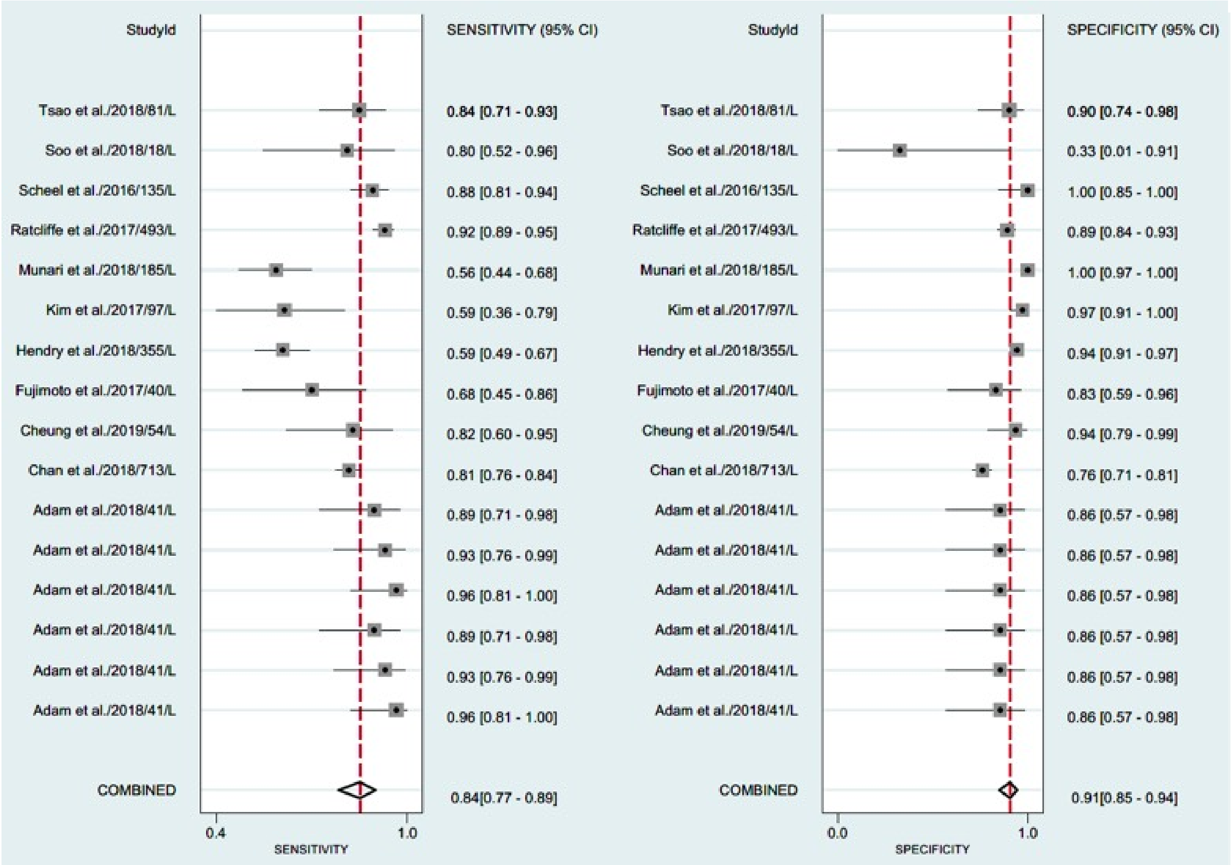

Figure 8 PD-L1 IHC pharmDx 28-8 (candidate) vs. Ventana PD-L1 (SP263) (RS) for 50% TPS Cut-off

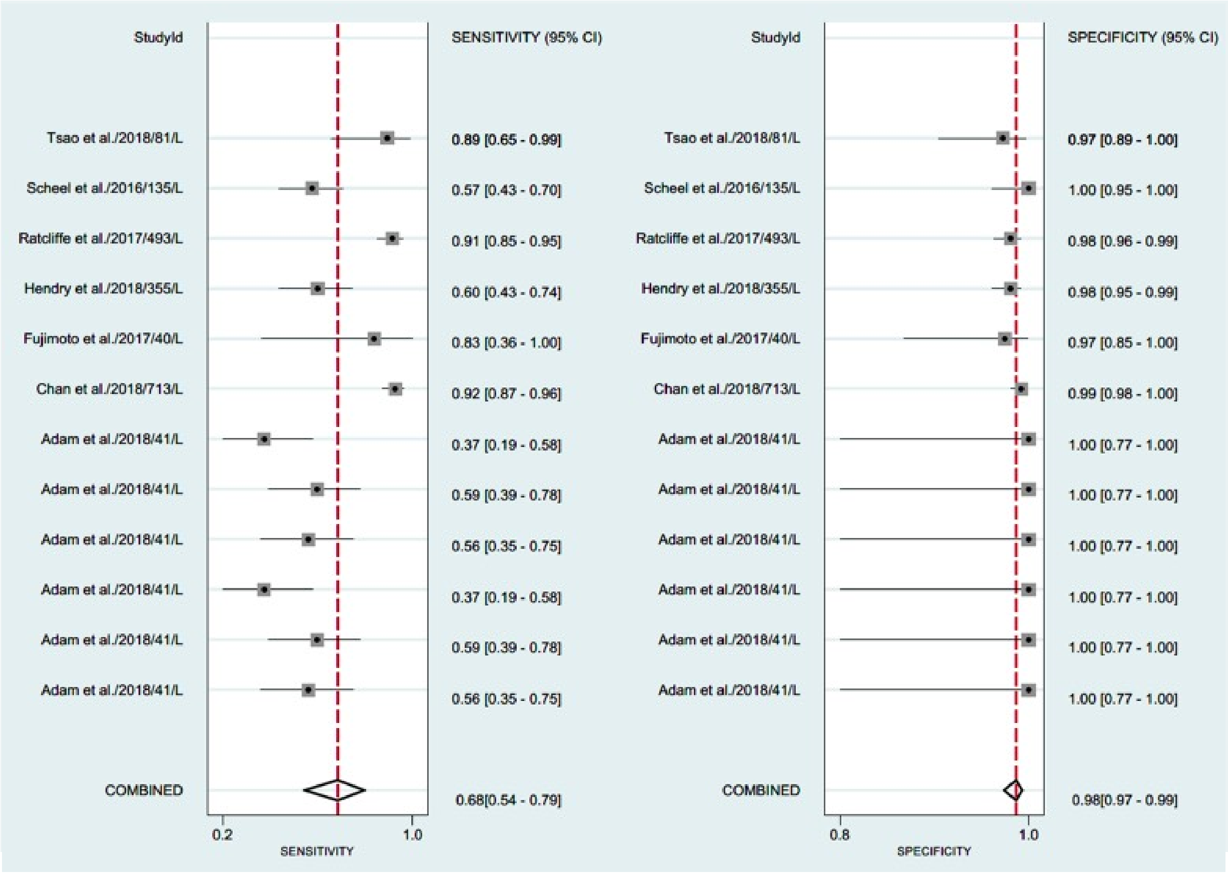

Figure 9 PD-L1 IHC pharmDx 28-8 (candidate) vs. Ventana PD-L1 (SP263) (RS) for 1% TPS Cut-off

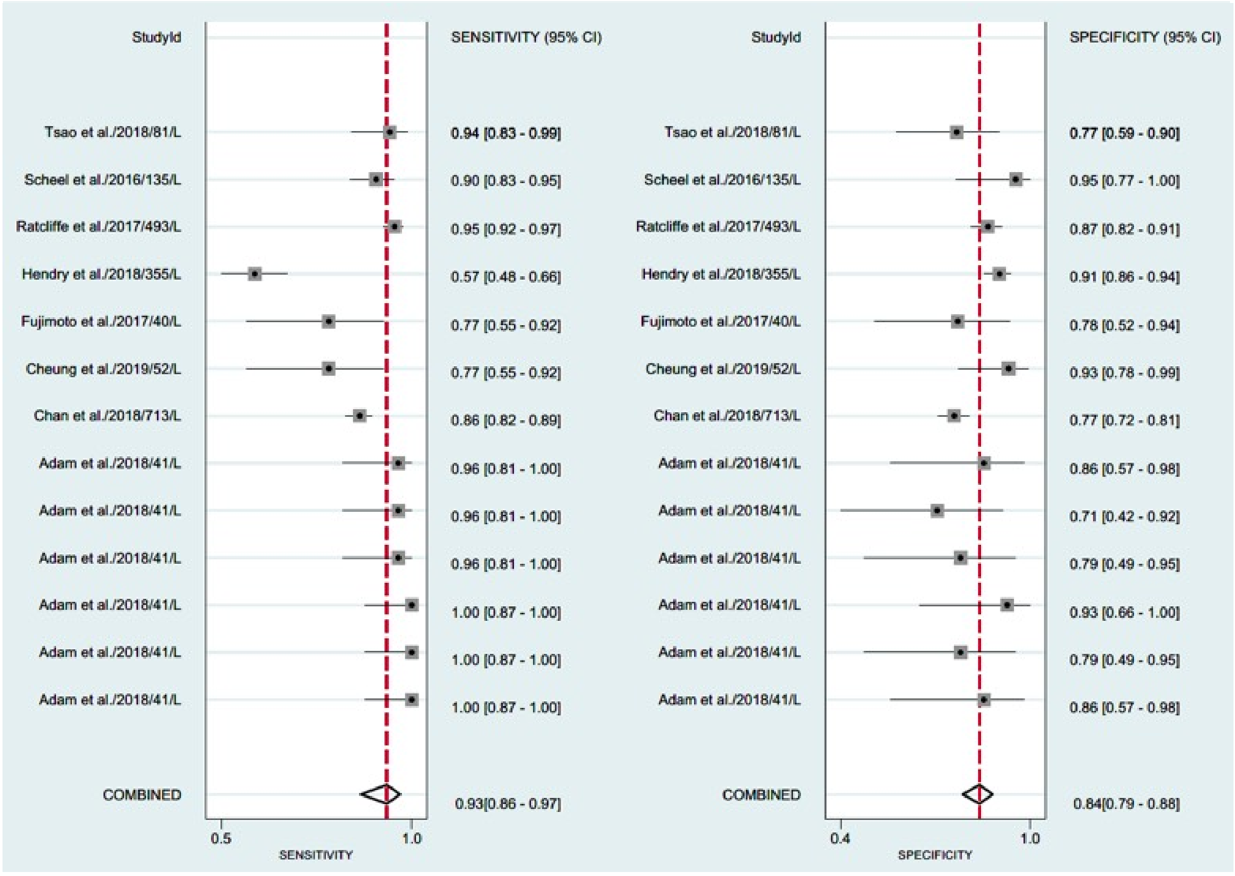

Figure 10

Ventana PD-L1 (SP142) (candidate) vs. Ventana PD-L1 (SP263) (RS) for 1% TPS Cut-off

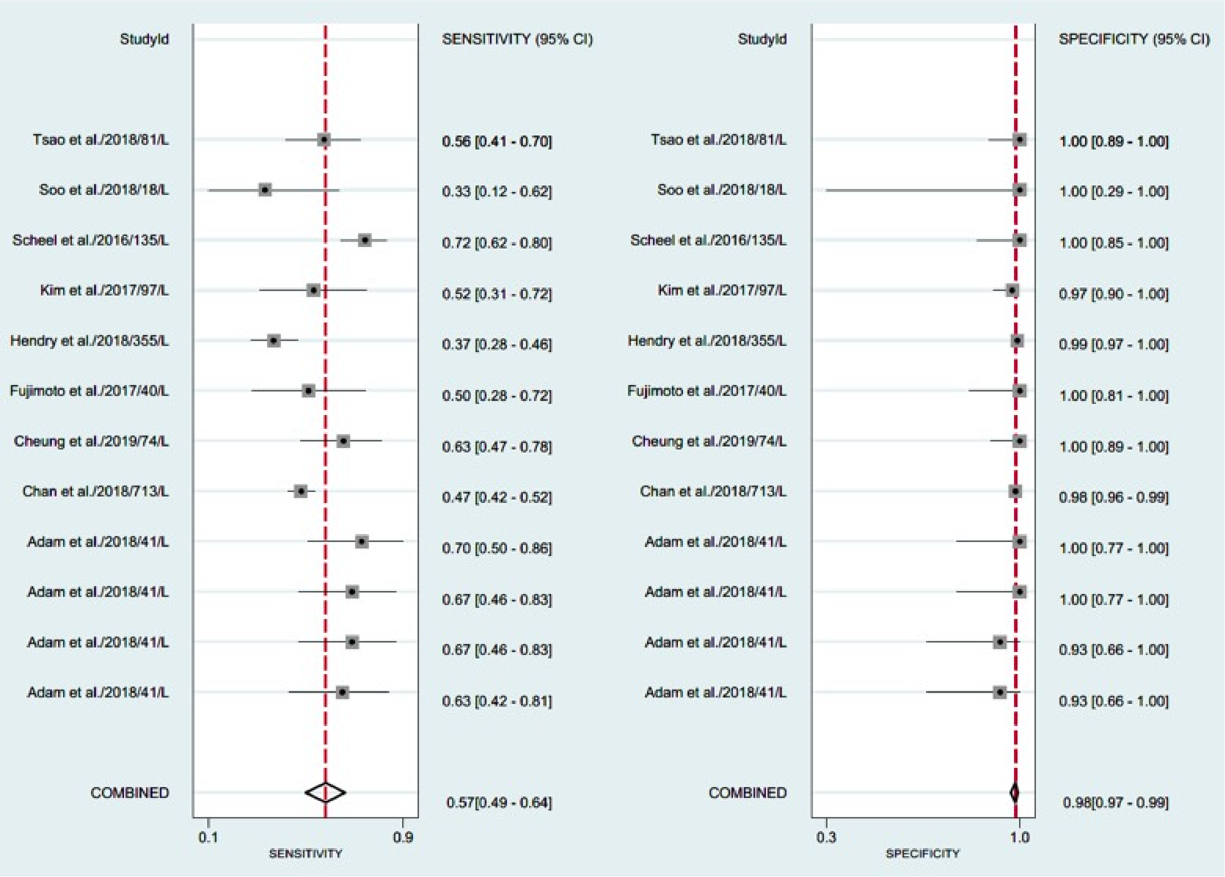

Figure 11

E1L3N LDT (candidate) vs. Ventana PD-L1 (SP263) (RS) for 1% TPS Cut-off

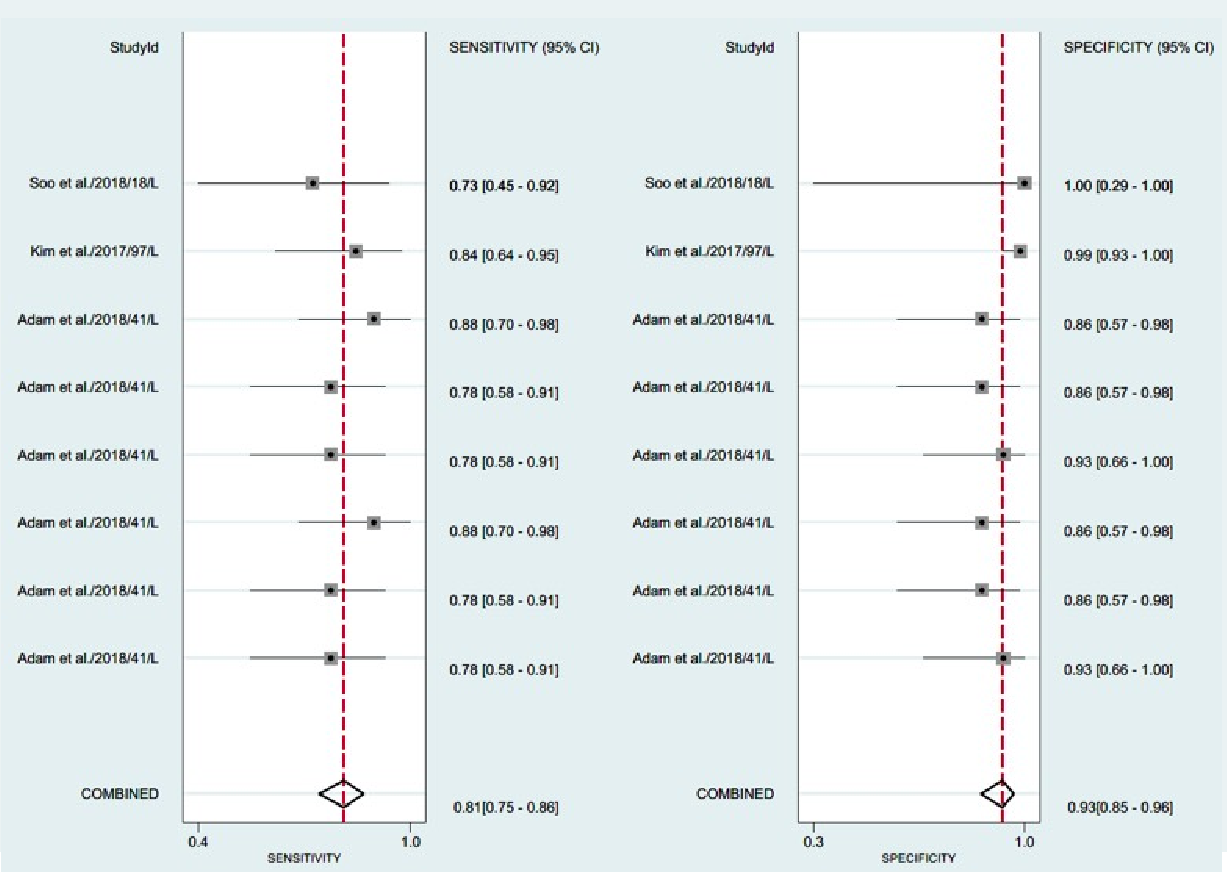

Figure 12

28-8 LDT (candidate) vs. PD-L1 IHC pharmDx 28-8 (RS) at 1% TPS Cut-off

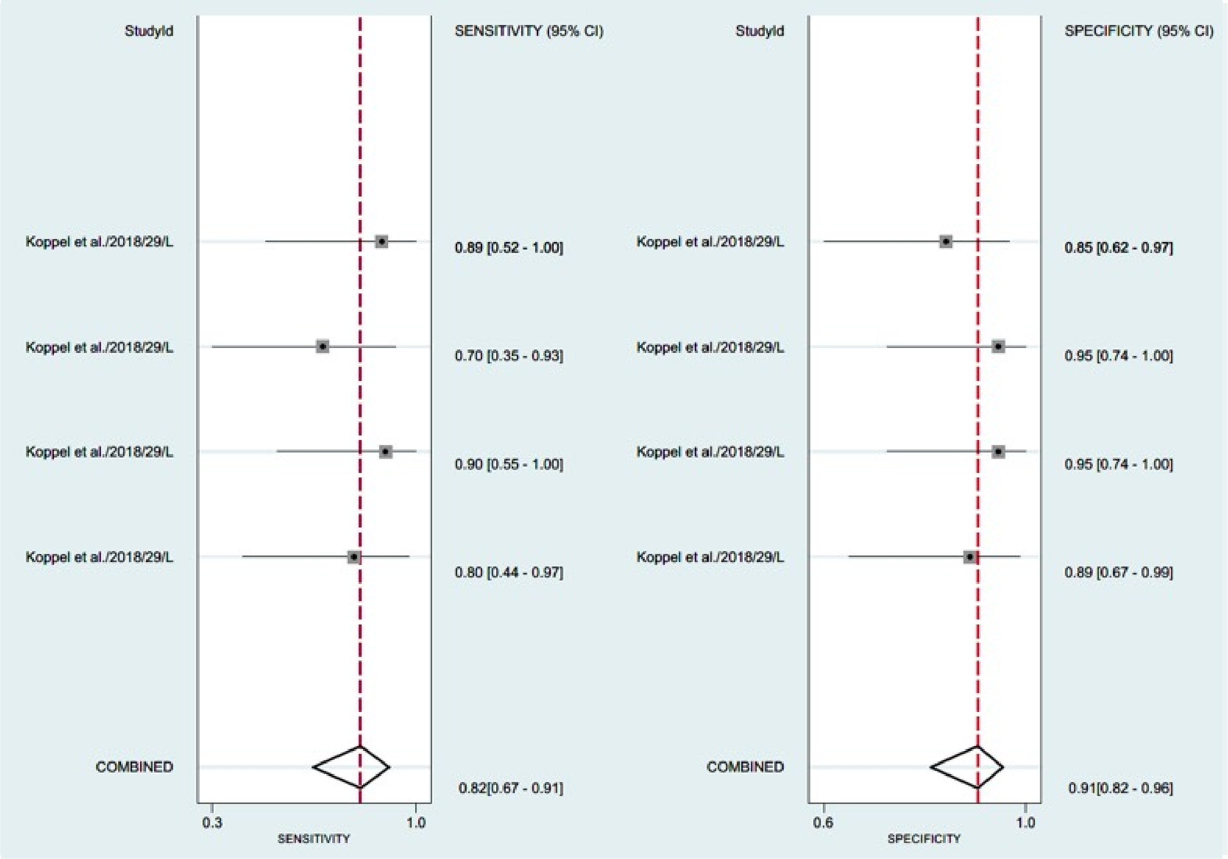

Figure 13

28-8 LDT (candidate) vs. PD-L1 IHC pharmDx 22C3 (RS) at 50% TPS Cut-off

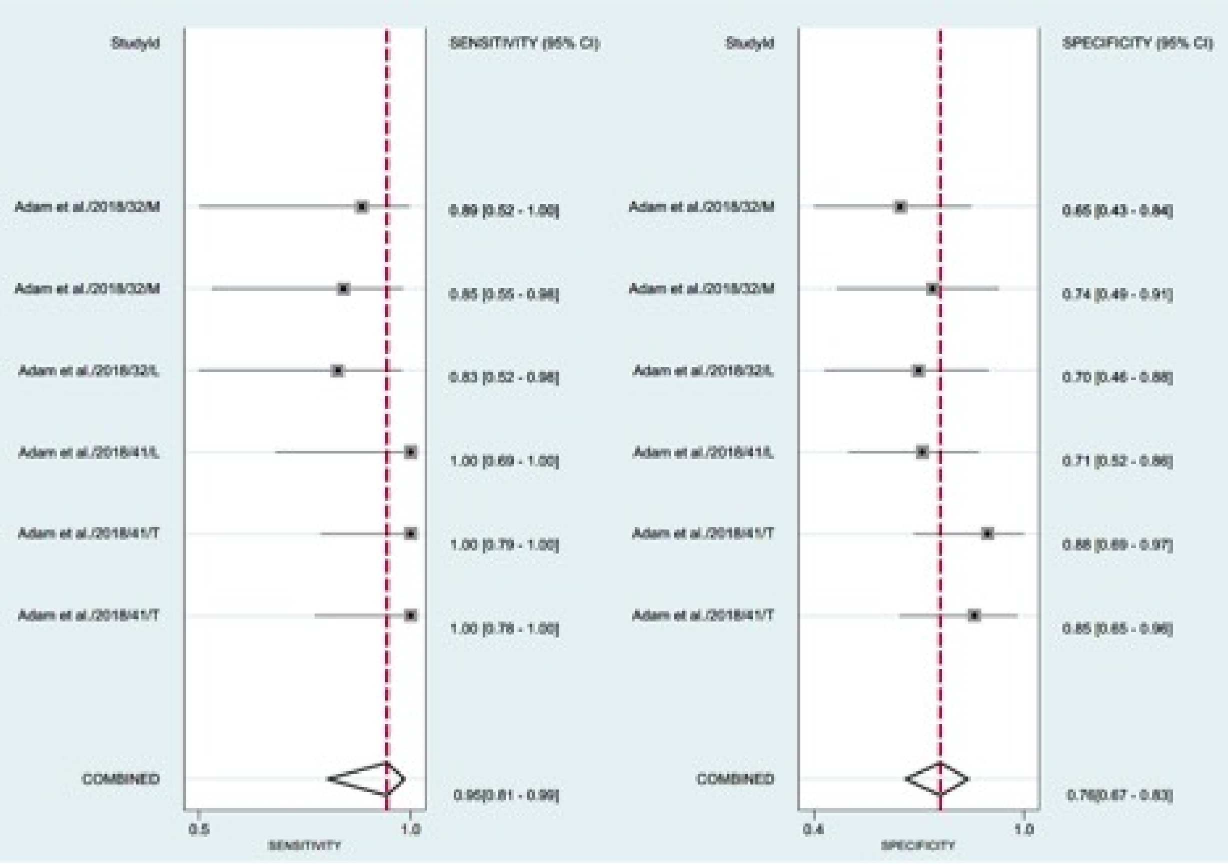

Supplement: Supplementary file 4 — Supplemetary Figures 2 - 13 [file 41379_2019_327_MOESM4_ESM.pdf]
