# Supplementary material for: “Interchangeability” of PD-L1 immunohistochemistry assays: a meta-analysis of diagnostic accuracy
Source: Mod Pathol. 2019 Aug 5;33(1):4–17. doi: 10.1038/s41379-019-0327-4 (PMC6927905; doi:10.1038/s41379-019-0327-4)

Figure 1: Funnel plots with pseudo 95% confidence limits of PD-L1 IHC pharmDx 28-8 (candidate) vs. PD-L1 IHC pharmDx 22C3 (GS) for 50% TPS Cut-off

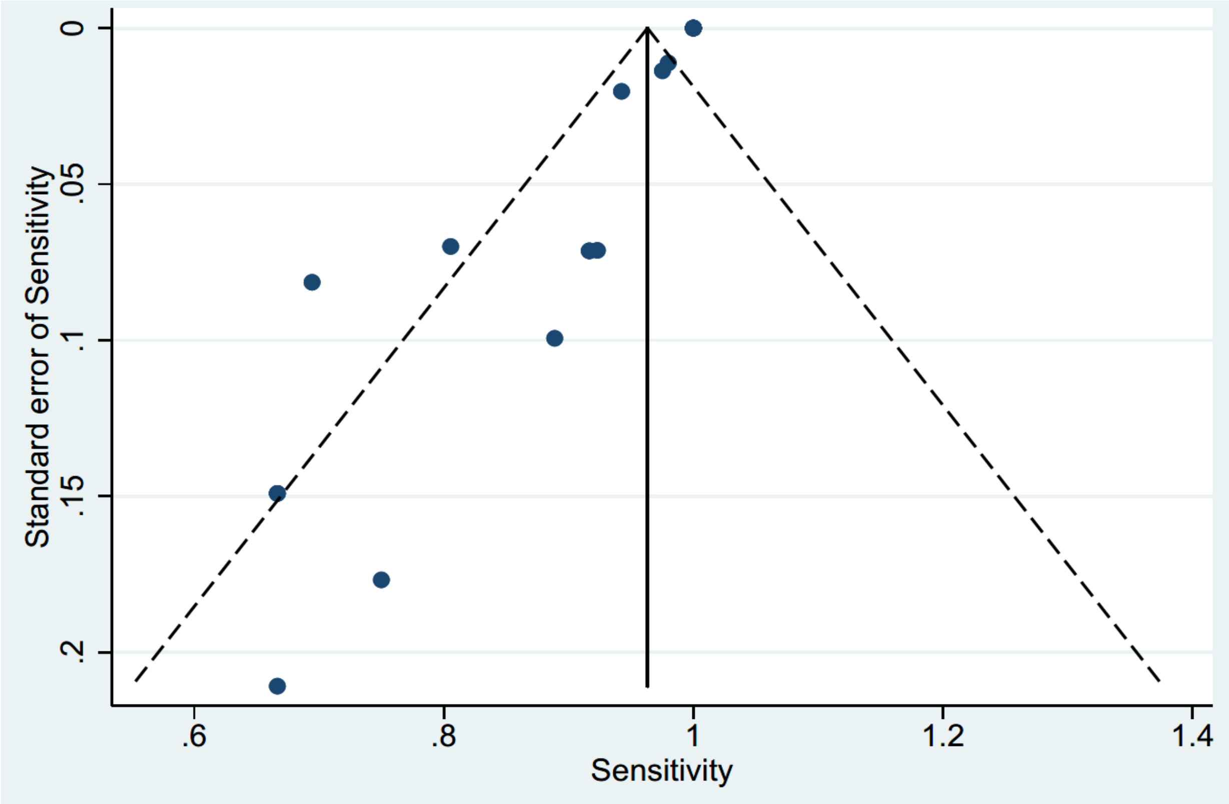

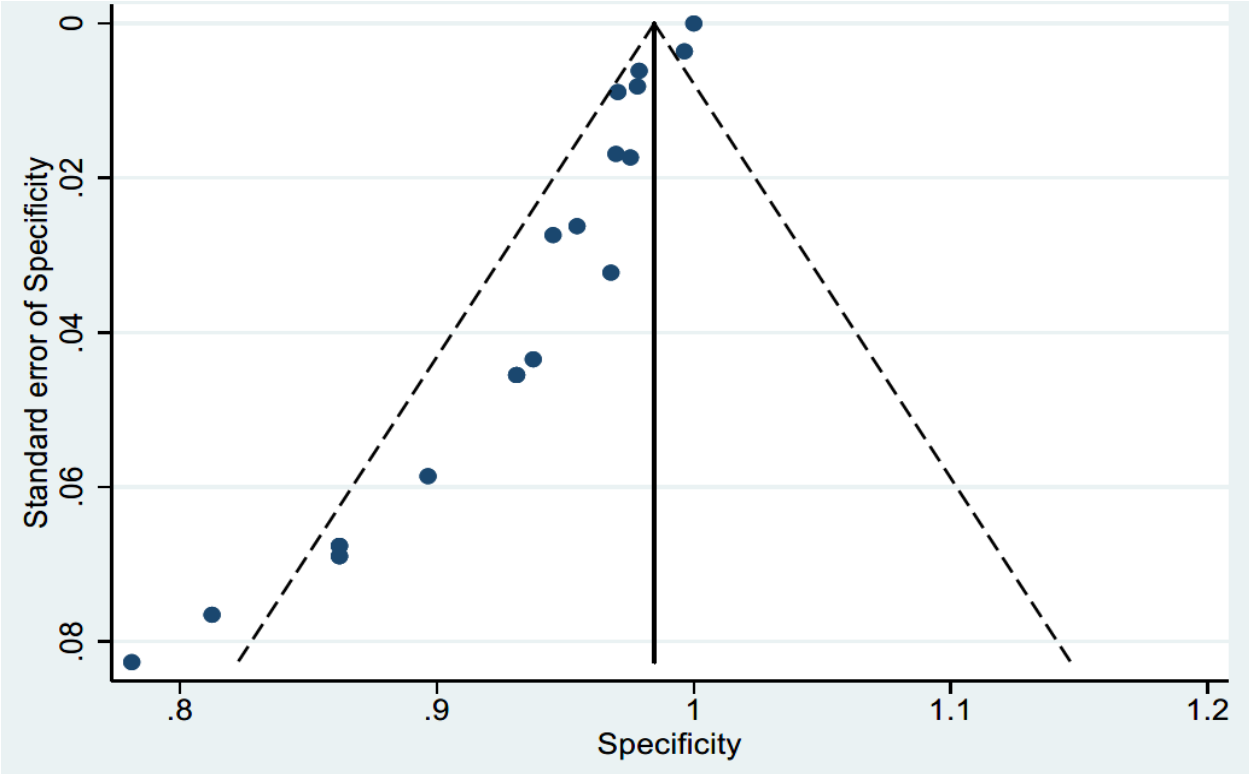

Figure 2: Funnel plots with pseudo 95% confidence limits of PD-L1 IHC pharmDx 28-8 (candidate) vs. PD-L1 IHC pharmDx 22C3 (GS) for 1% TPS Cut-off

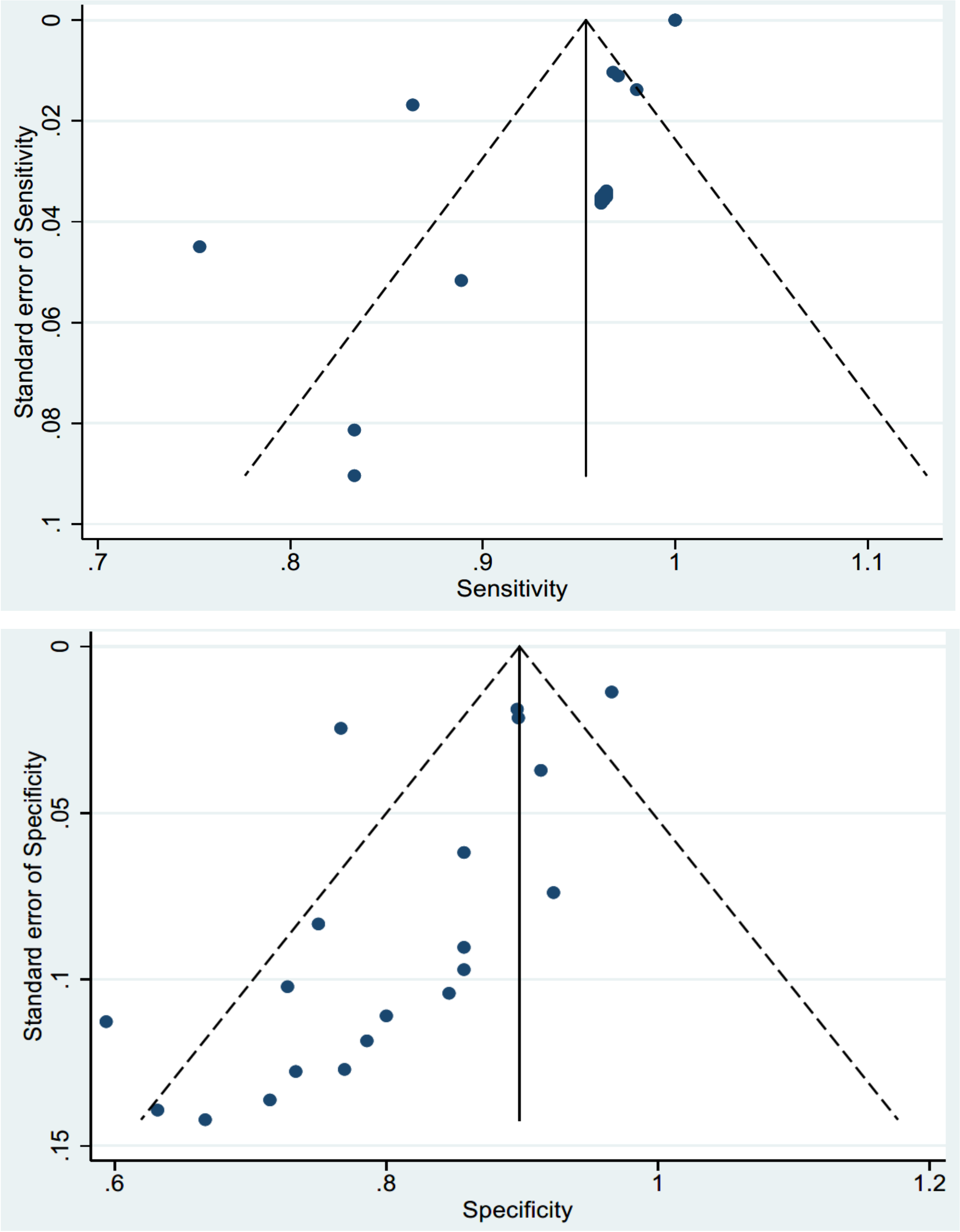

Figure 3: Funnel plots with pseudo 95% confidence limits of Ventana PD-L1 (SP263) (candidate) vs. PD-L1 IHC pharmDx 22C3 (GS) for 50% TPS Cut-off

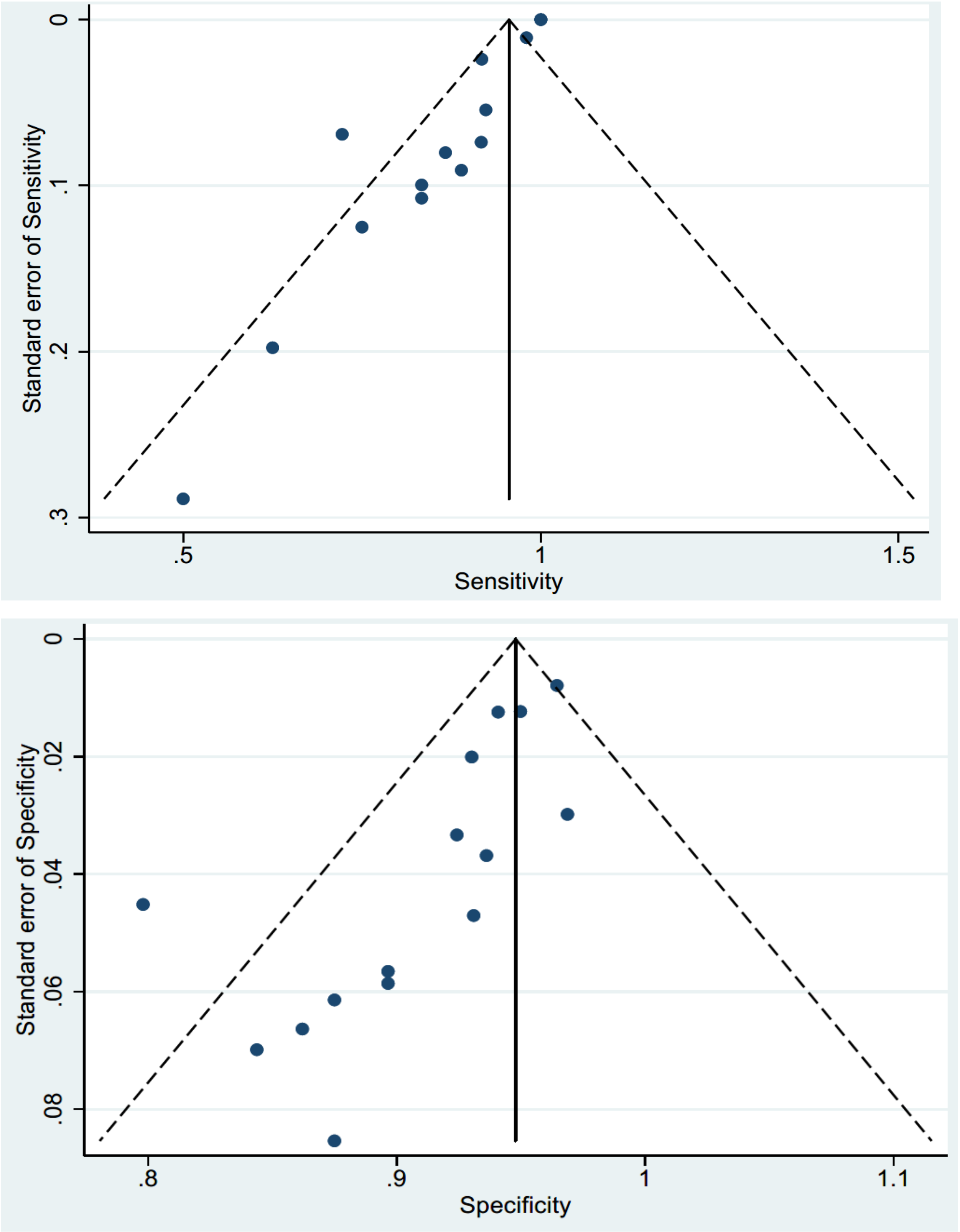

Figure 4: Funnel plots with pseudo 95% confidence limits of Ventana PD-L1 (SP263) (candidate) vs. PD-L1 IHC pharmDx 22C3 (GS) for 1% TPS Cut-off

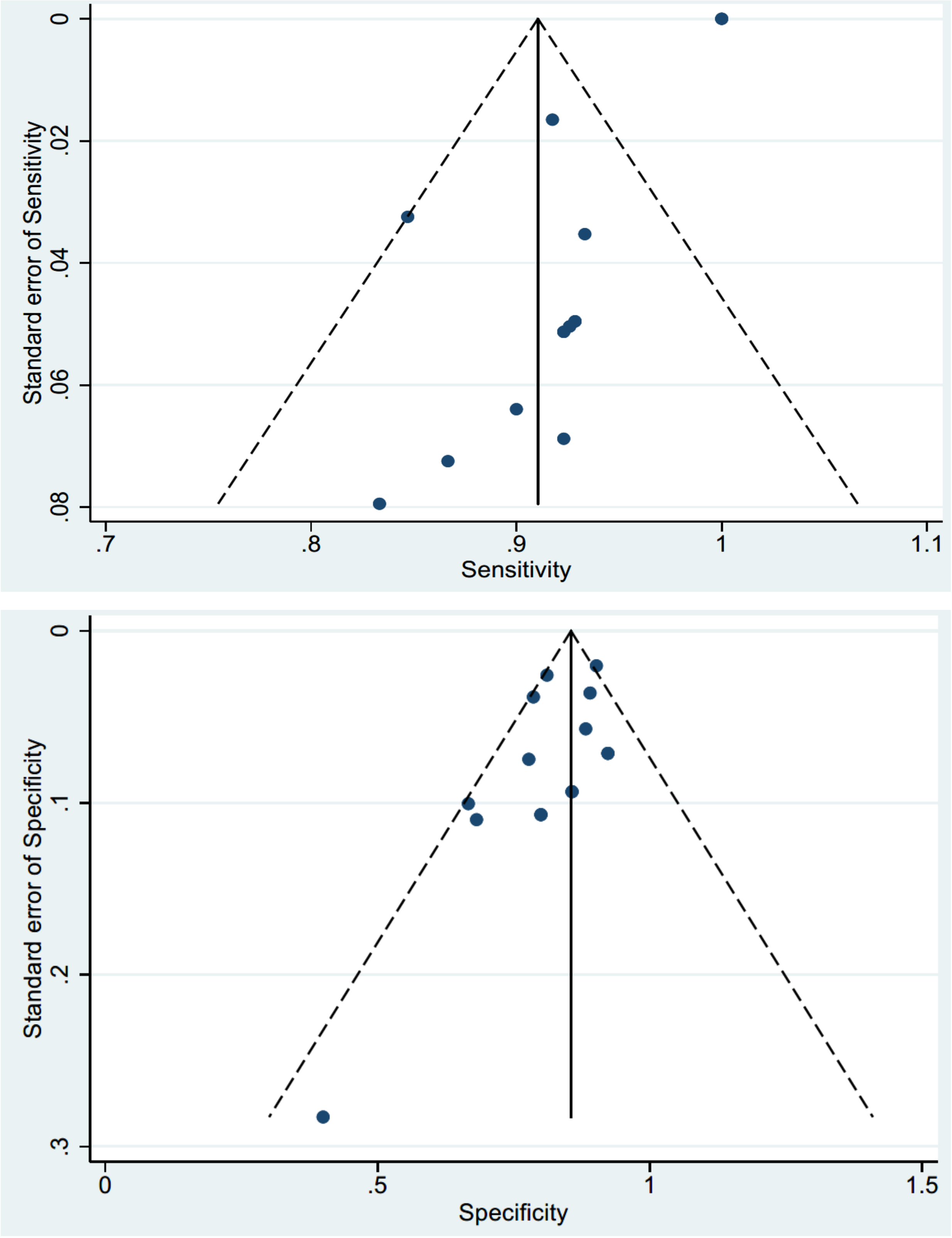

Figure 5: Funnel plots with pseudo 95% confidence limits of Ventana PD-L1 (SP142) (candidate) vs. PD-L1 IHC pharmDx 22C3 (GS) for 50% TPS Cut-off

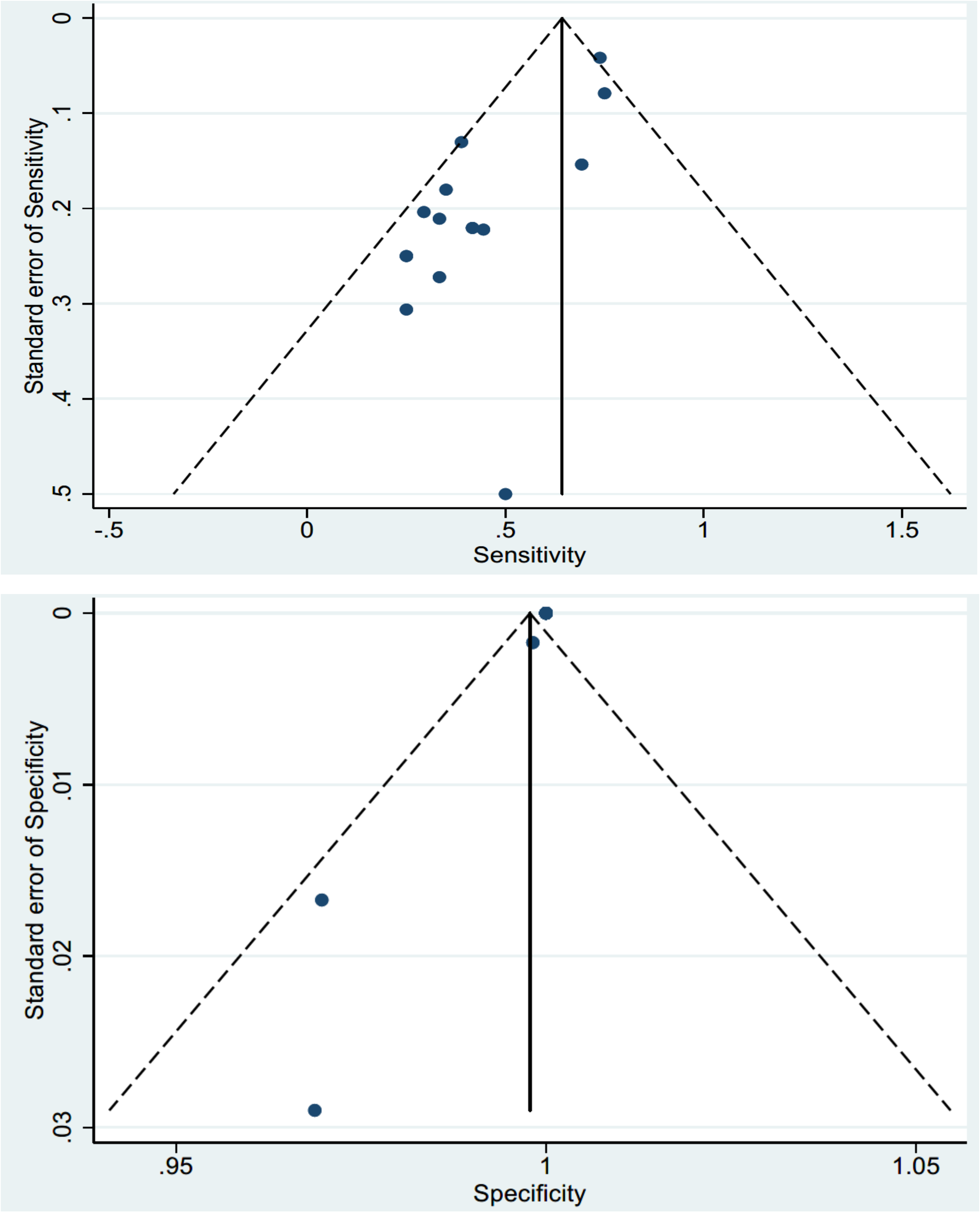

Figure 6: Funnel plots with pseudo 95% confidence limits of Ventana PD-L1 (SP142) (candidate) vs. PD-L1 IHC pharmDx 22C3 (GS) for 1% TPS Cut-off

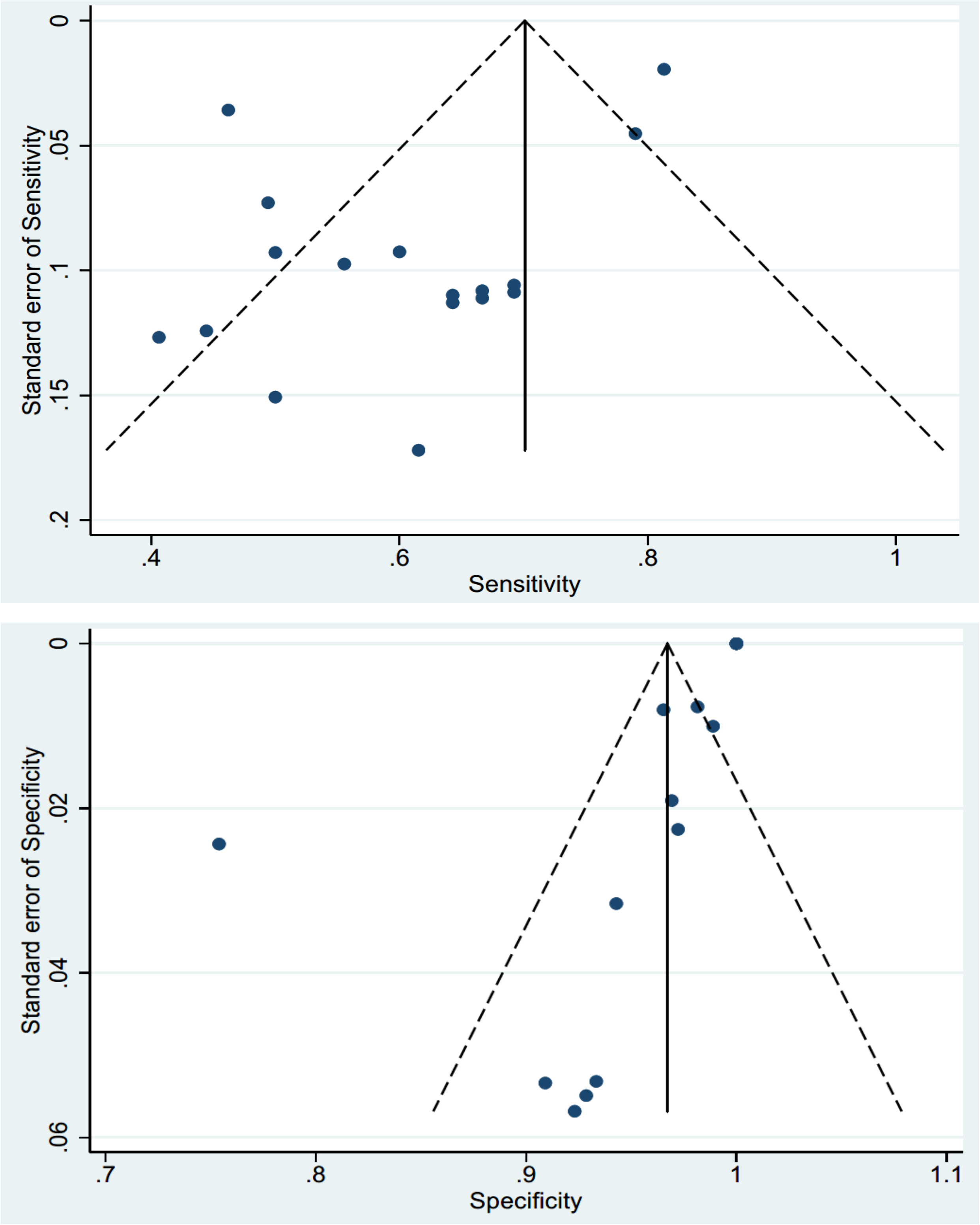

Figure 7: Funnel plots with pseudo 95% confidence limits of 22C3 LDT (candidate) vs. PD-L1 IHC pharmDx 22C3 (GS) for 1% TPS Cut-off

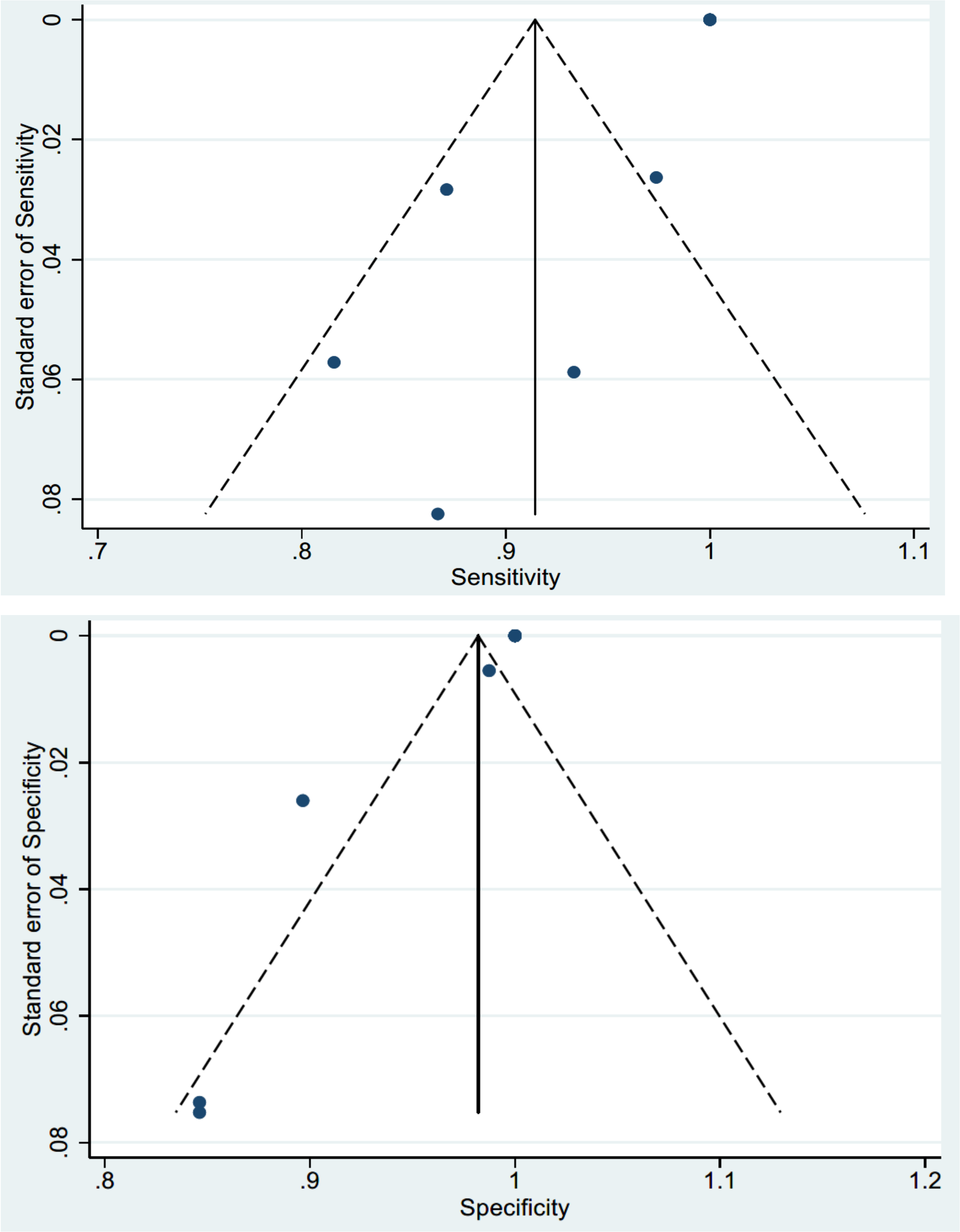

Figure 8: Funnel plots with pseudo 95% confidence limits of E1L3N LDT (candidate) vs. PD-L1 IHC pharmDx 22C3 (GS) for 50% TPS Cut-off

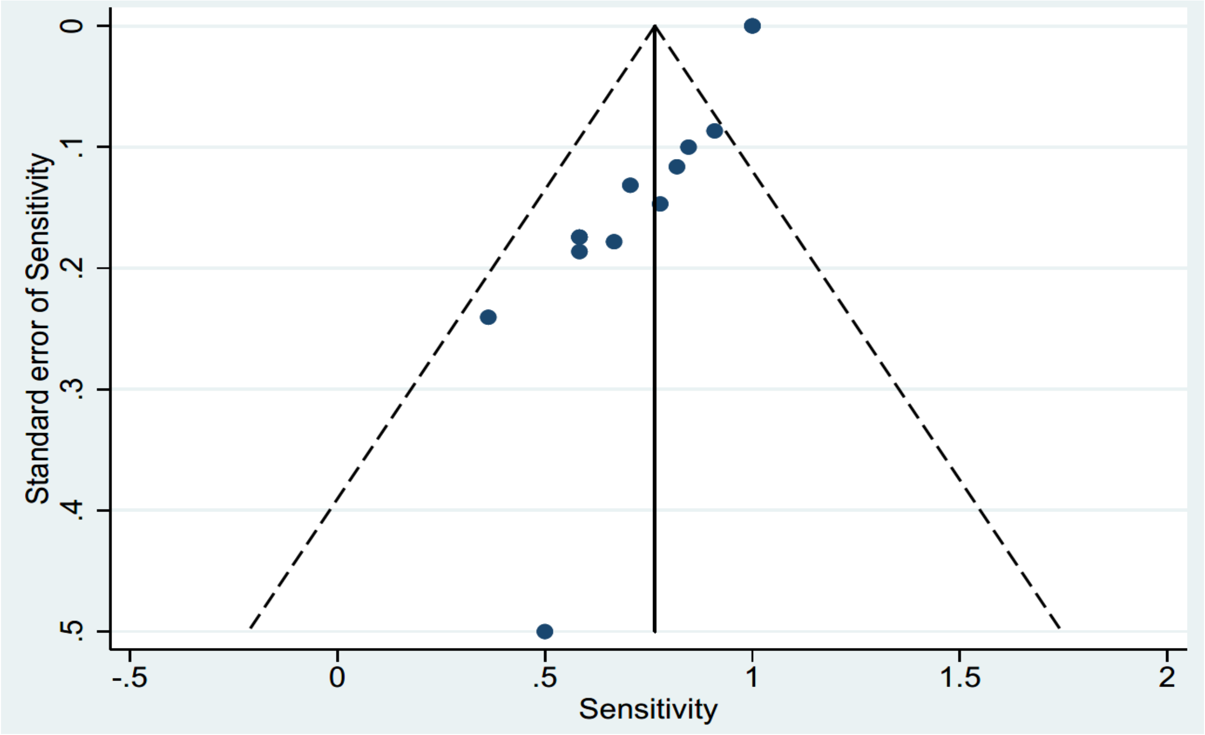

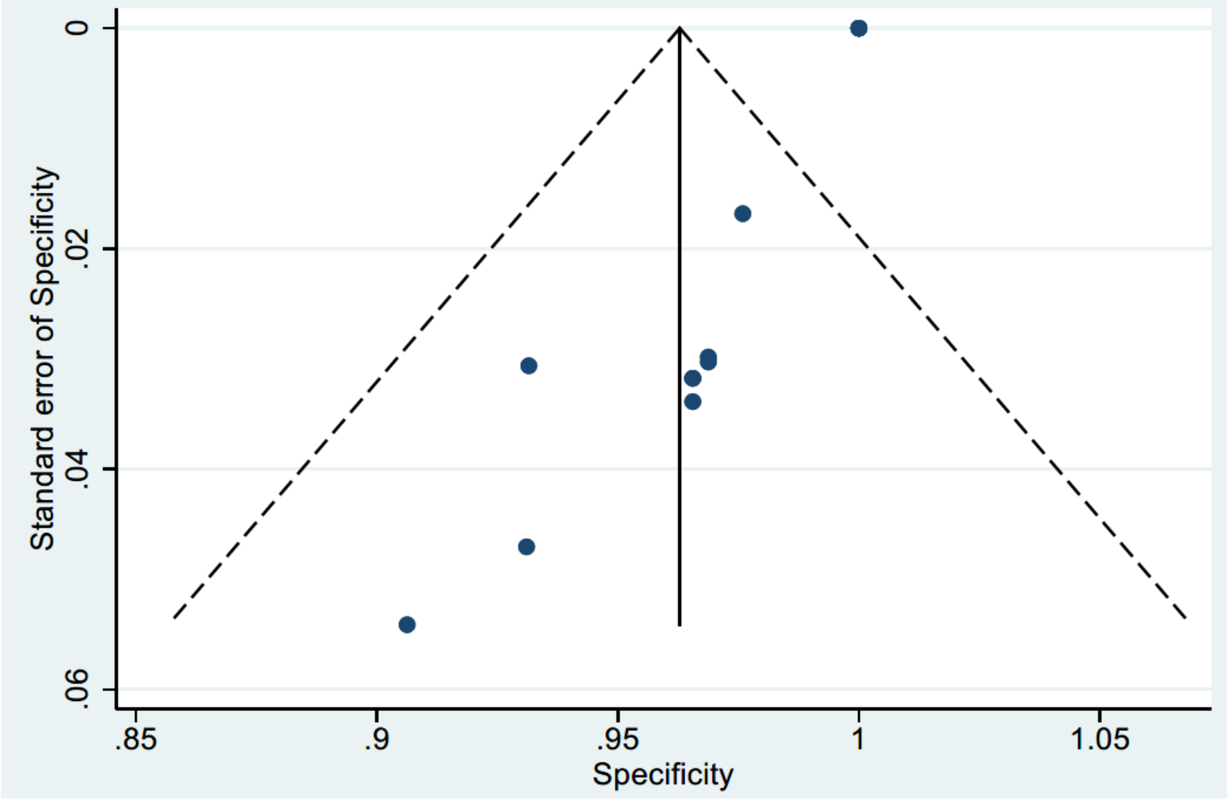

Figure 9: Funnel plots with pseudo 95% confidence limits of E1L3N LDT (candidate) vs. PD-L1 IHC pharmDx 22C3 (GS) for 1% TPS Cut-off

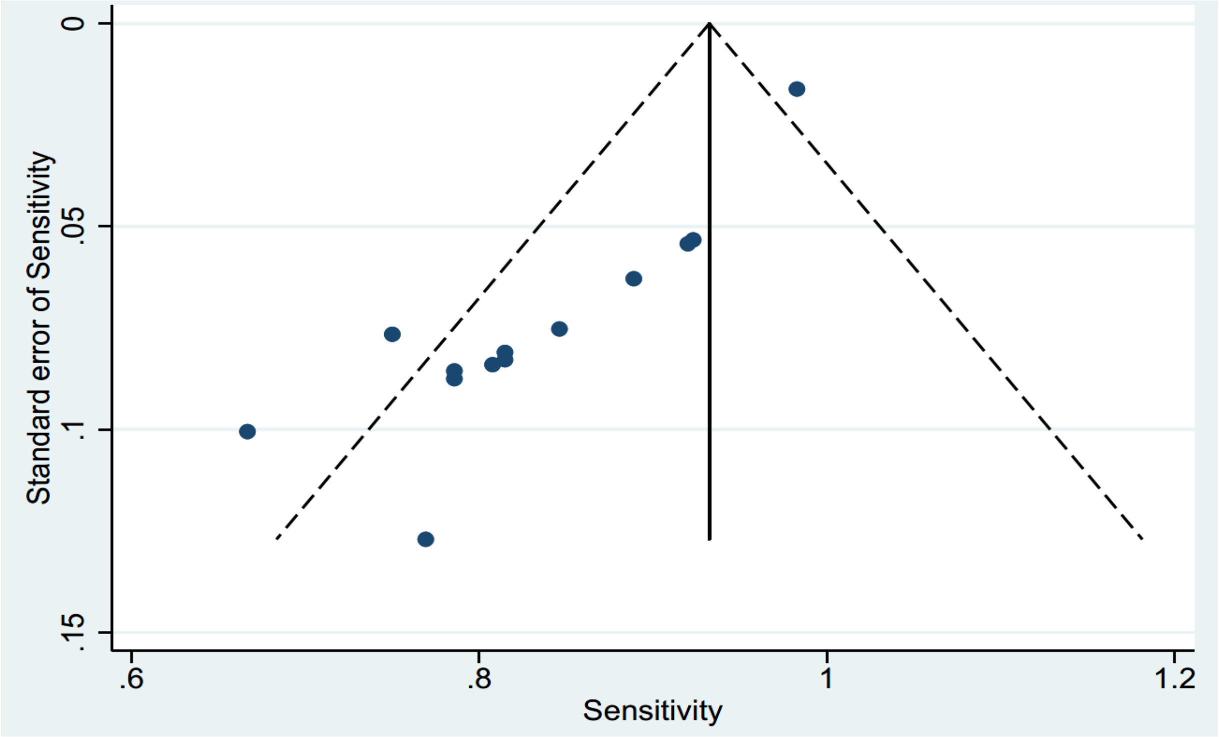

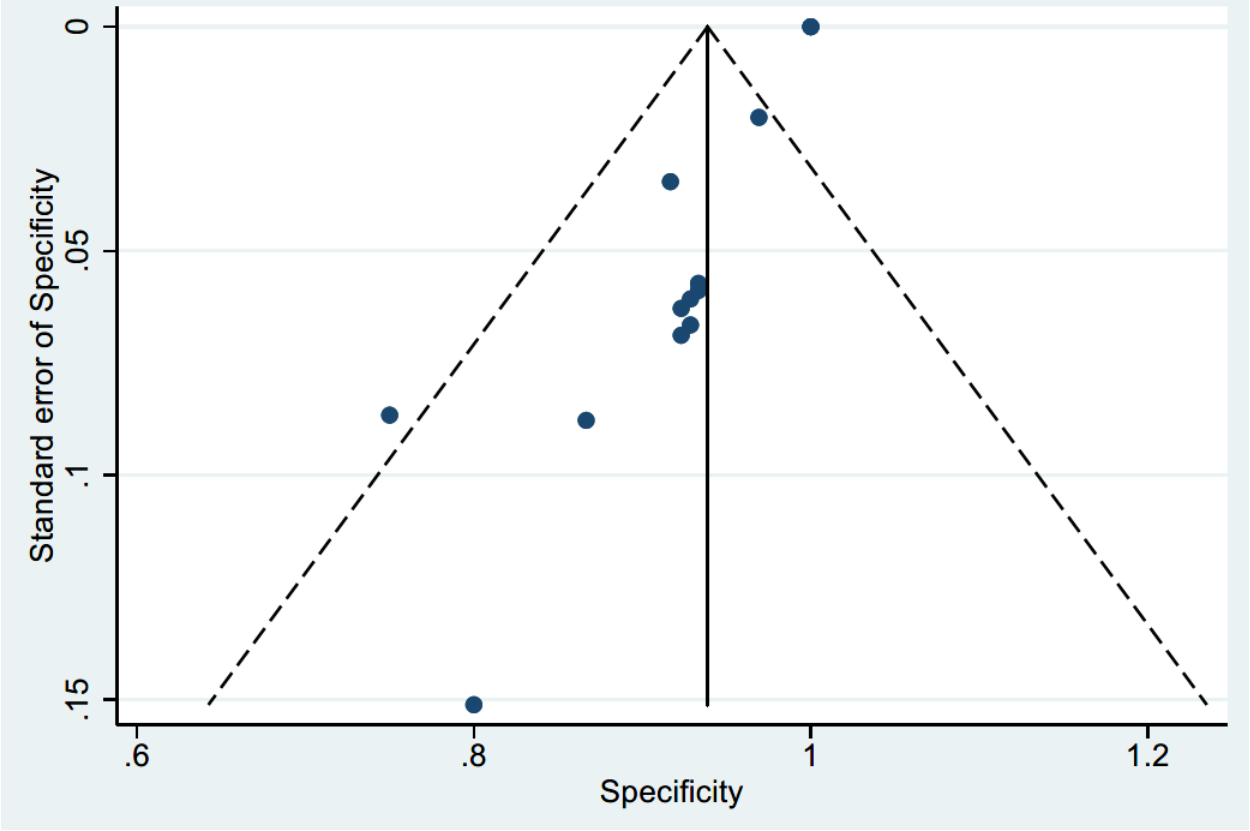

Figure 10: Funnel plots with pseudo 95% confidence limits of PD-L1 IHC pharmDx 22C3 (candidate) vs. PD-L1 IHC pharmDx 28-8 (GS) for 1% TPS Cut-off

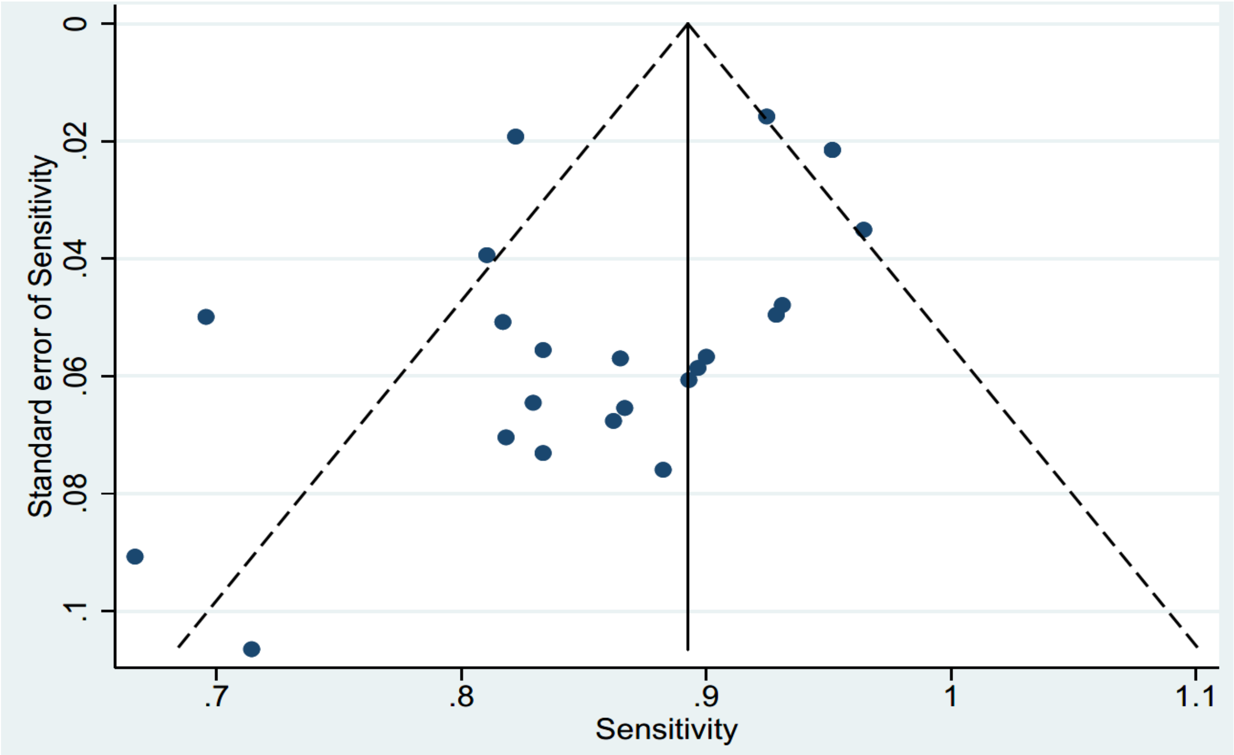

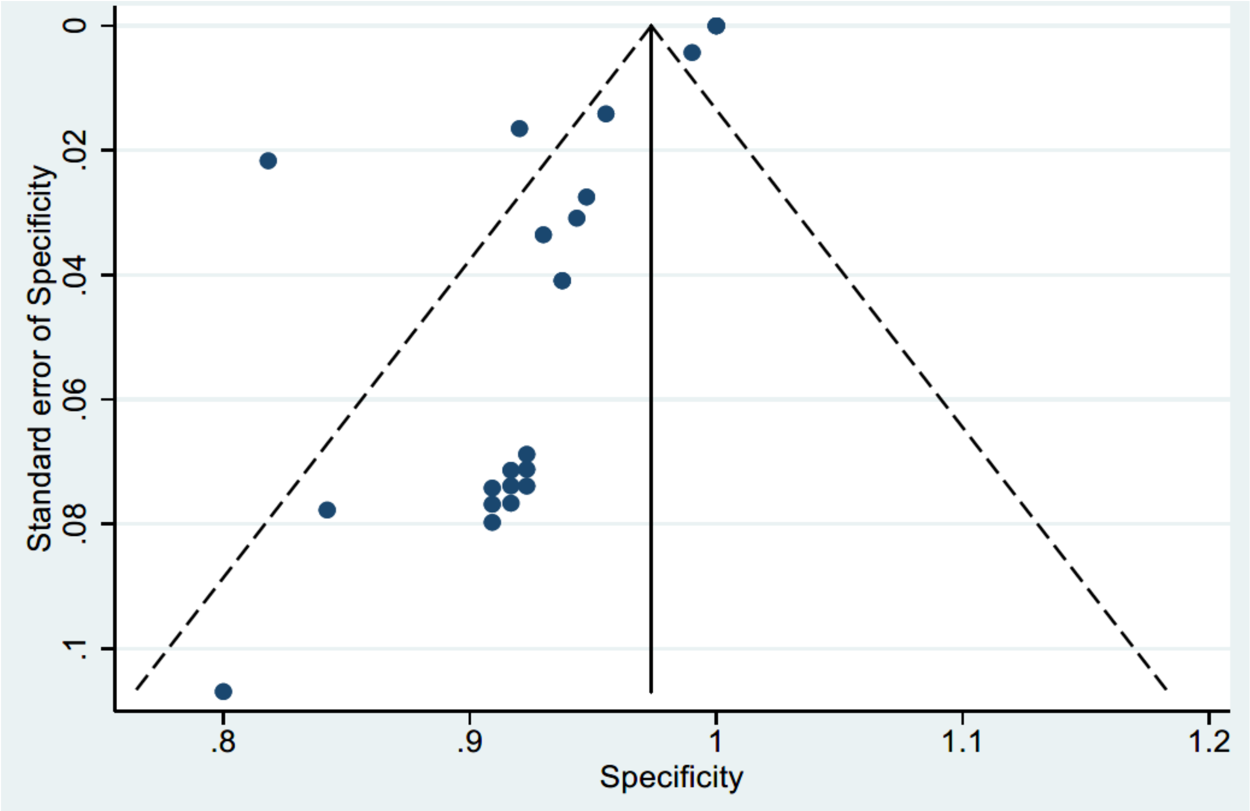

Figure 11: Funnel plots with pseudo 95% confidence limits of Ventana PD-L1 (SP263) (candidate) vs. PD-L1 IHC pharmDx 28-8 (GS) for 1% TPS Cut-off

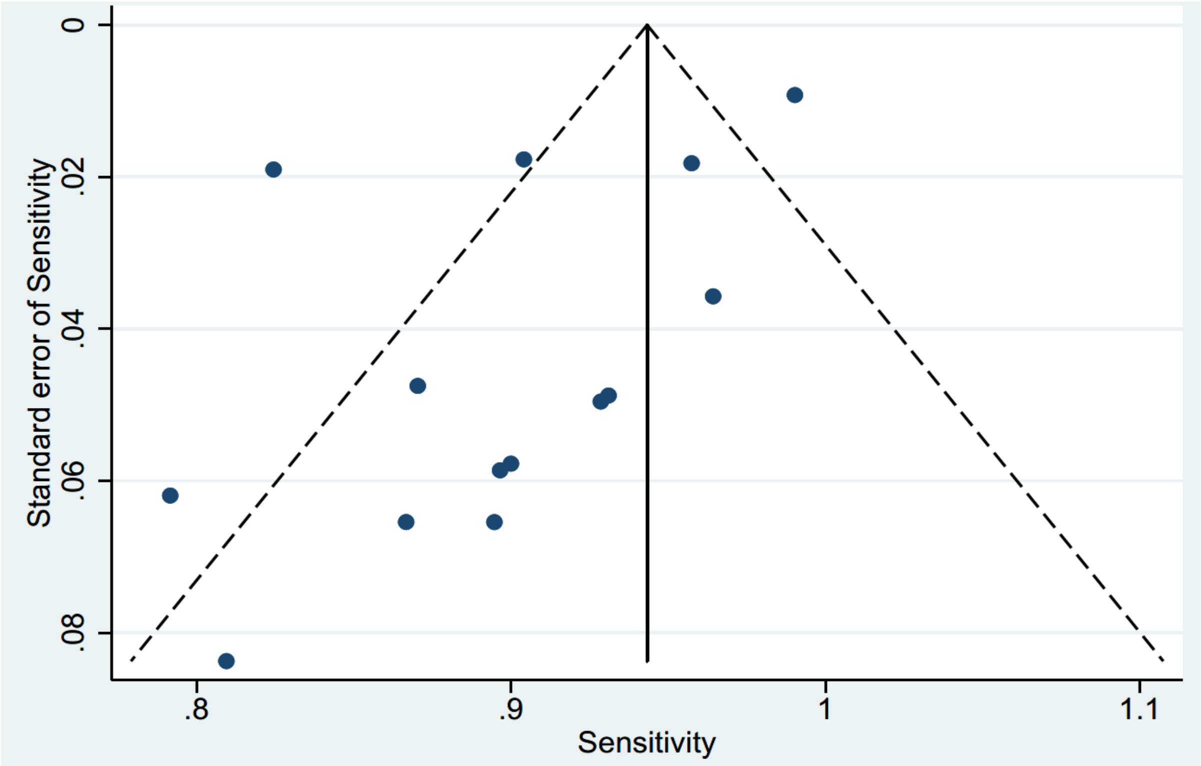

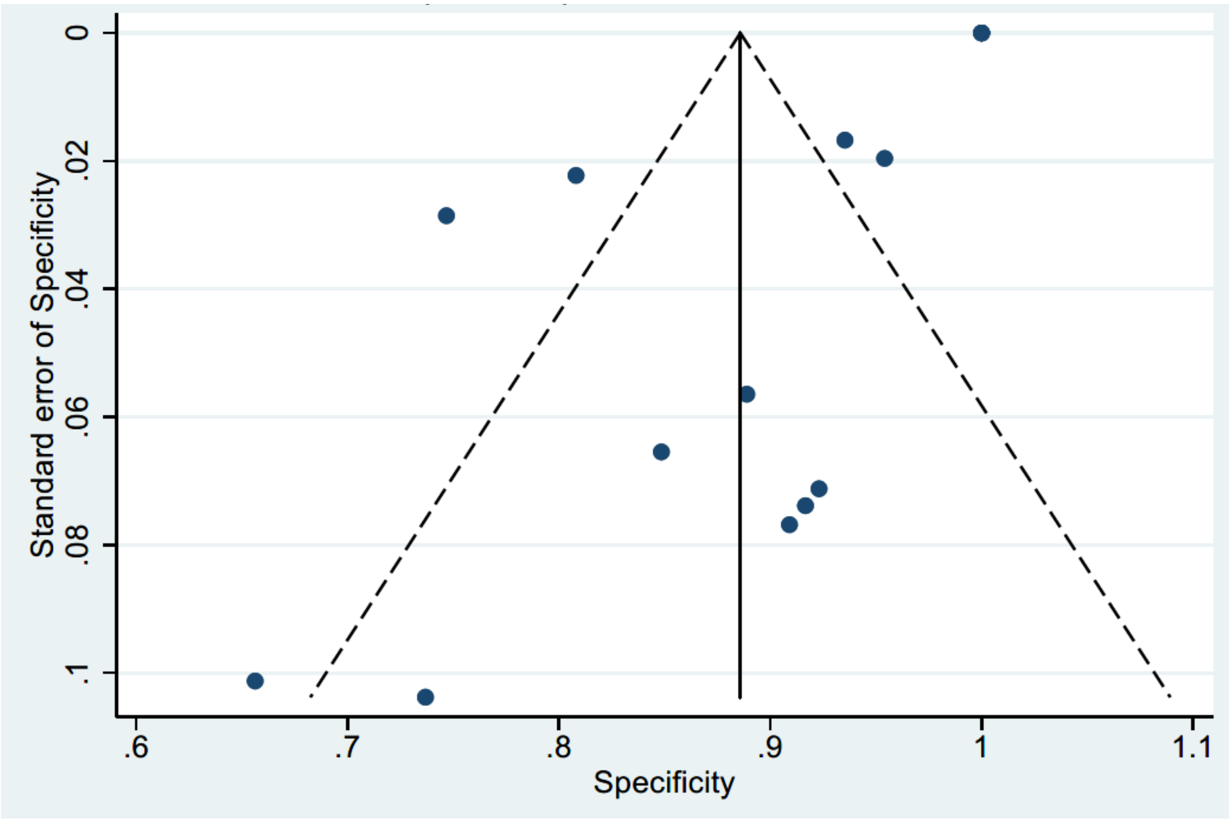

Figure 12: Funnel plots with pseudo 95% confidence limits of Ventana PD-L1 (SP142) (candidate) vs. PD-L1 IHC pharmDx 28-8 (GS) for 1% TPS Cut-off

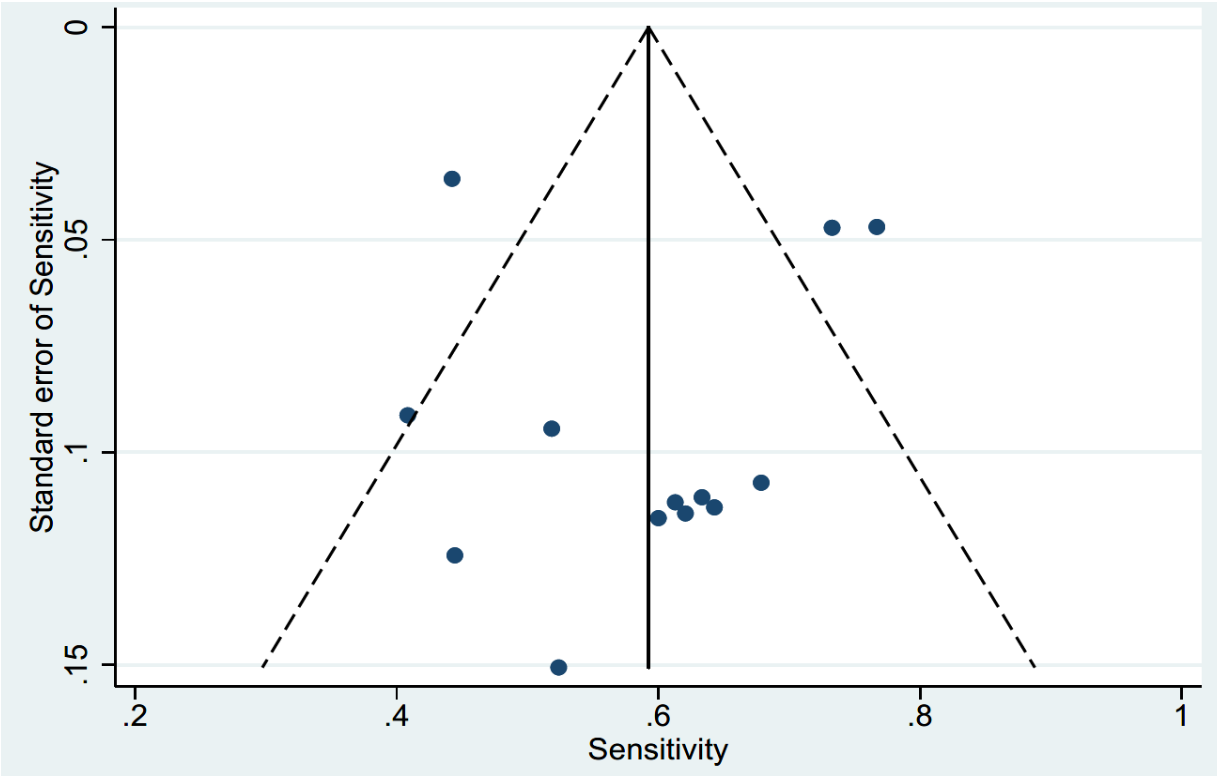

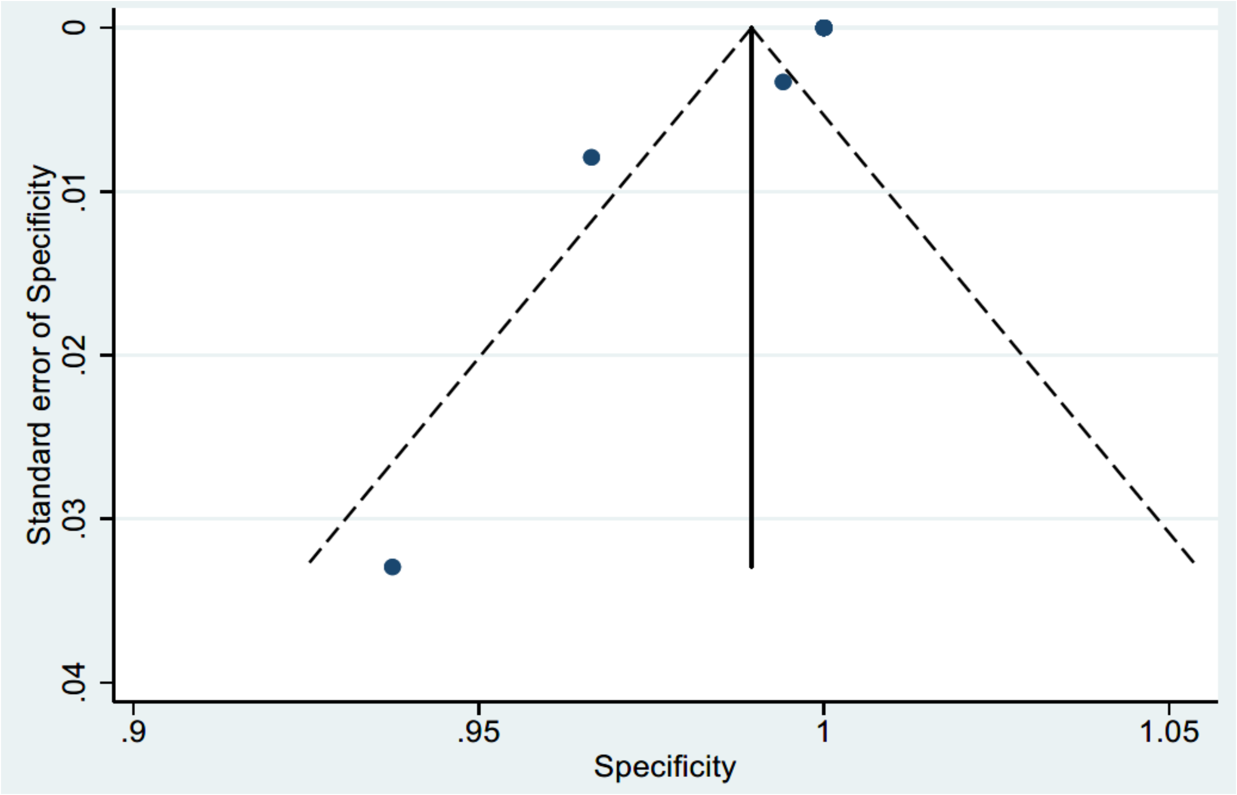

Figure 13: Funnel plots with pseudo 95% confidence limits of E1L3N LDT (candidate) vs. PD-L1 IHC pharmDx 28-8 (GS) for 1% TPS Cut-off

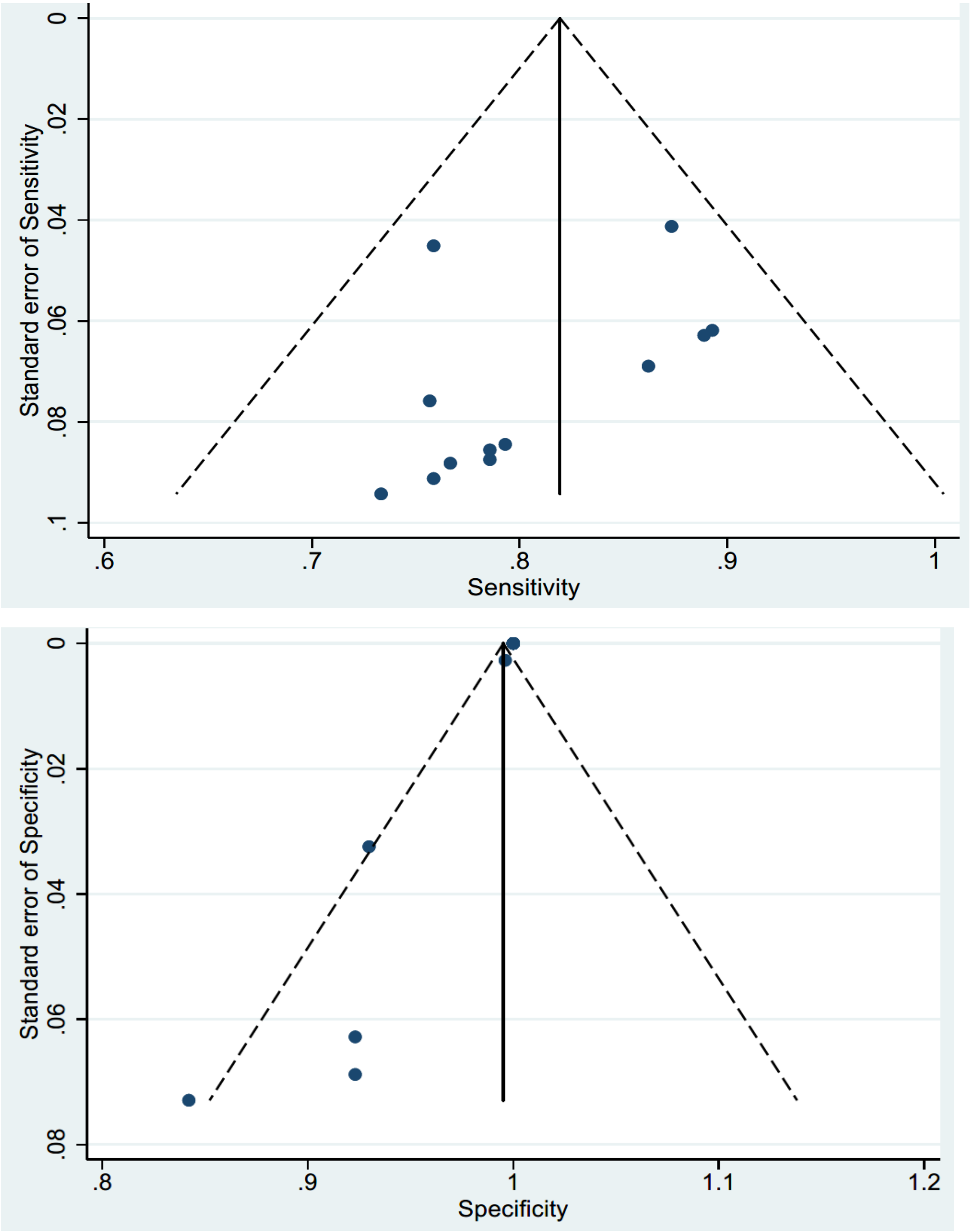

Figure 14: Funnel plots with pseudo 95% confidence limits of PD-L1 IHC pharmDx 22C3 (candidate) vs. Ventana PD-L1 (SP263) (GS) for 50% TPS Cut-off

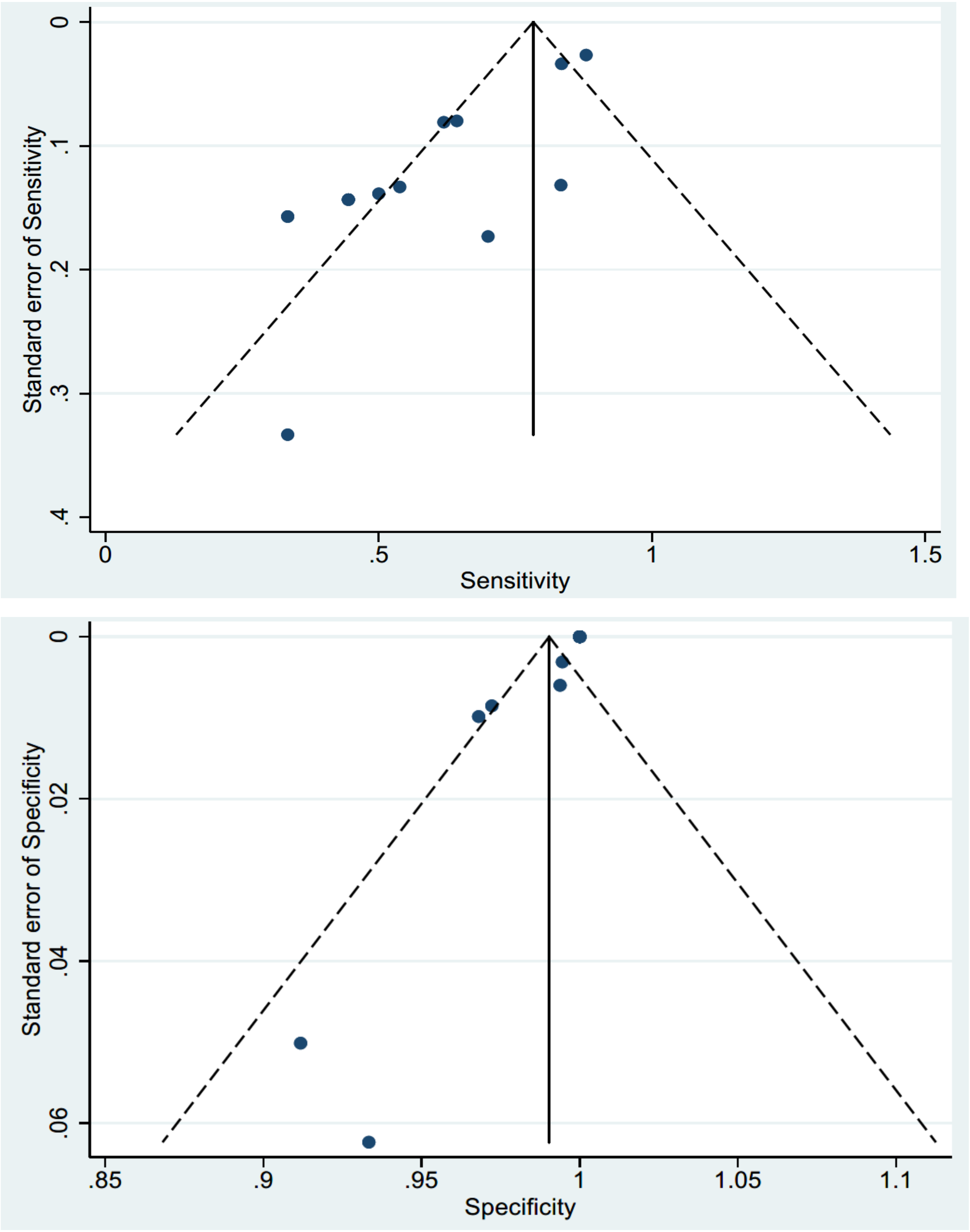

Figure 15: Funnel plots with pseudo 95% confidence limits of PD-L1 IHC pharmDx 22C3 (candidate) vs. Ventana PD-L1 (SP263) (GS) for 1% TPS Cut-off

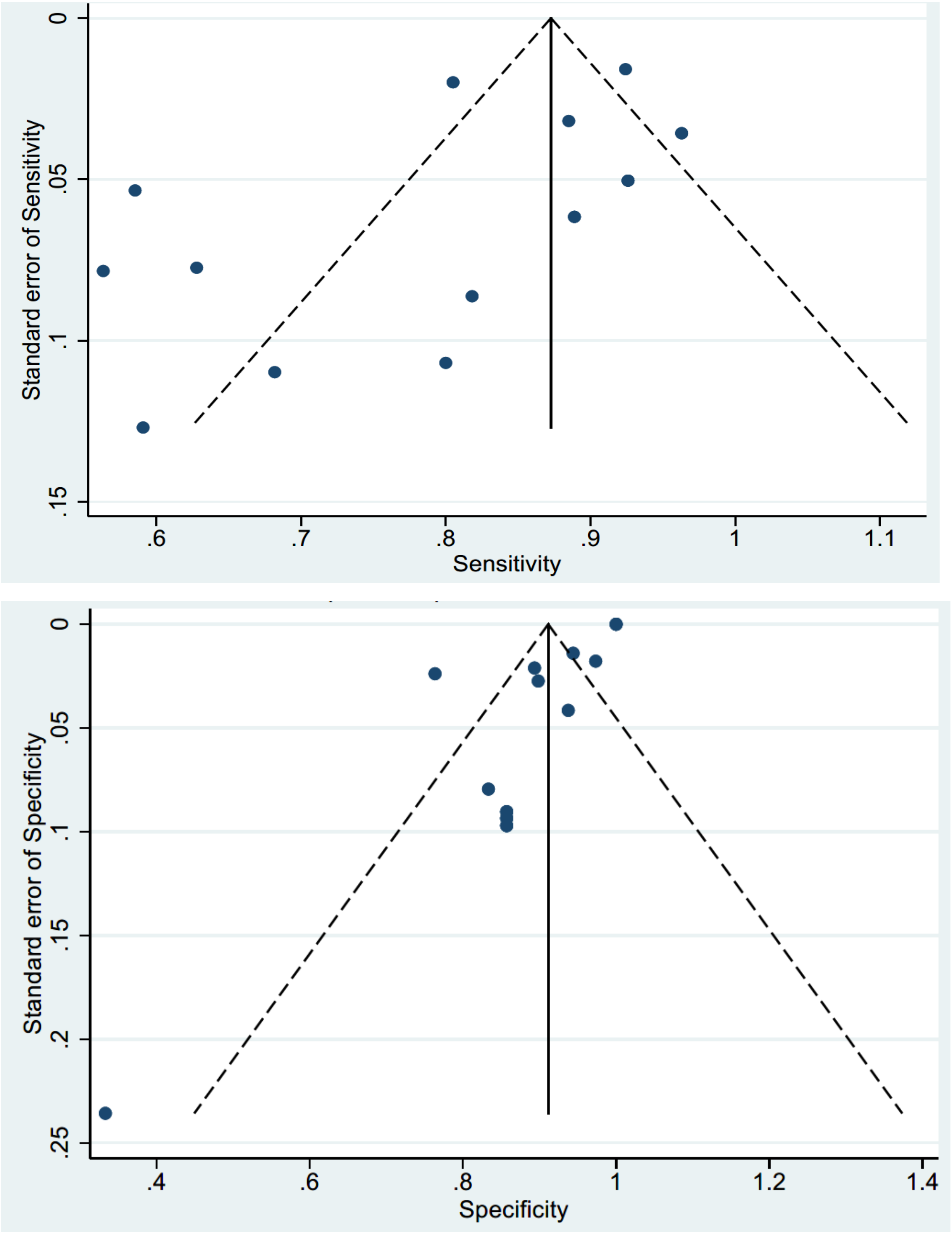

Figure 16: Funnel plots with pseudo 95% confidence limits of PD-L1 IHC pharmDx 28-8 (candidate) vs. Ventana PD-L1 (SP263) (GS) for 50% TPS Cut-off

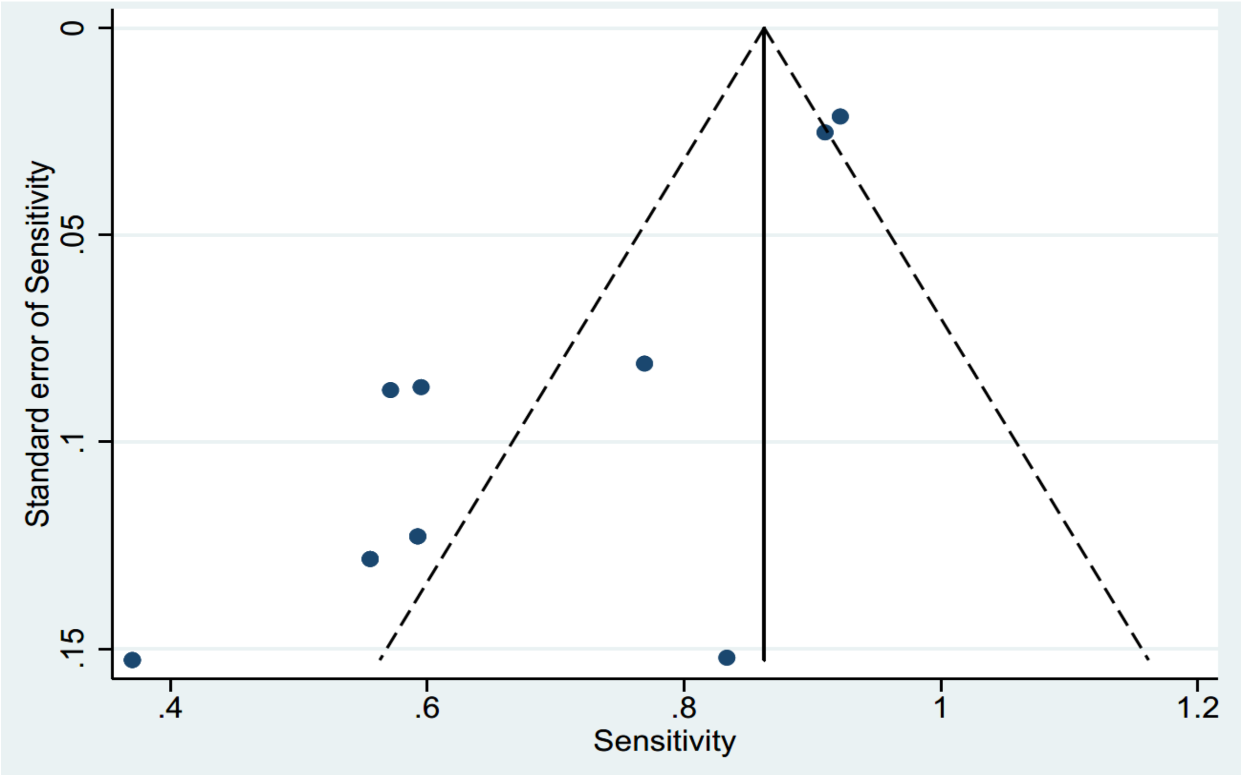

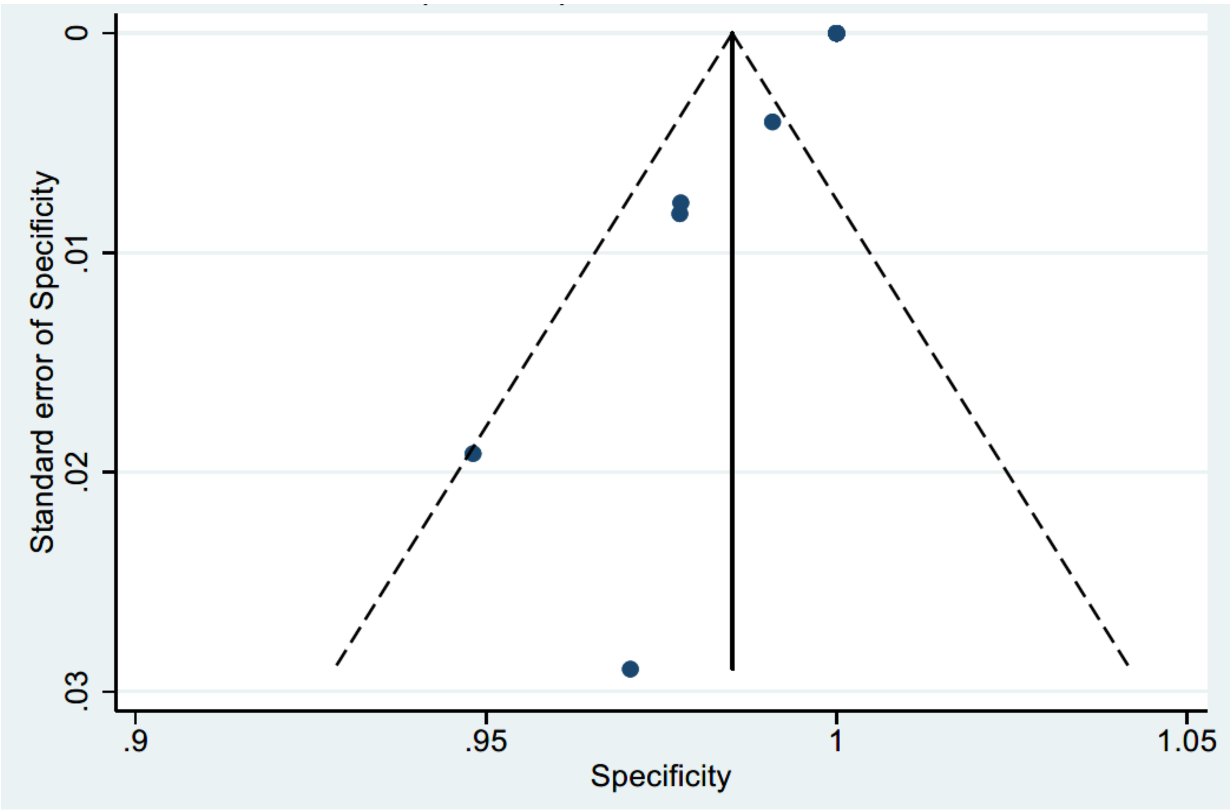

Figure 17: Funnel plots with pseudo 95% confidence limits of PD-L1 IHC pharmDx 28-8 (candidate) vs. Ventana PD-L1 (SP263) (GS) for 1% TPS Cut-off

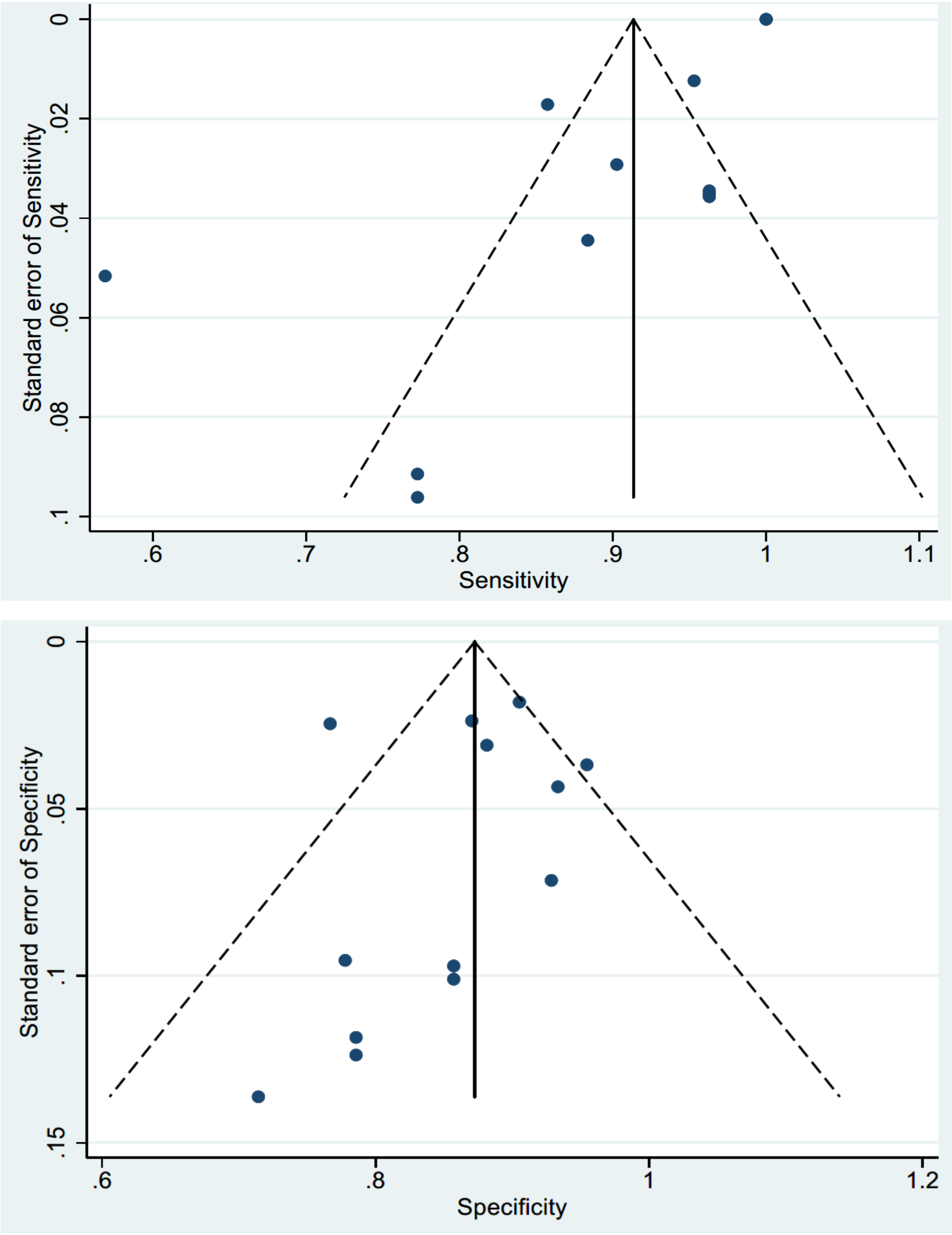

Figure 18: Funnel plots with pseudo 95% confidence limits of Ventana PD-L1 (SP142) (candidate) vs. Ventana PD-L1 (SP263) (GS) for 1% TPS Cut-off

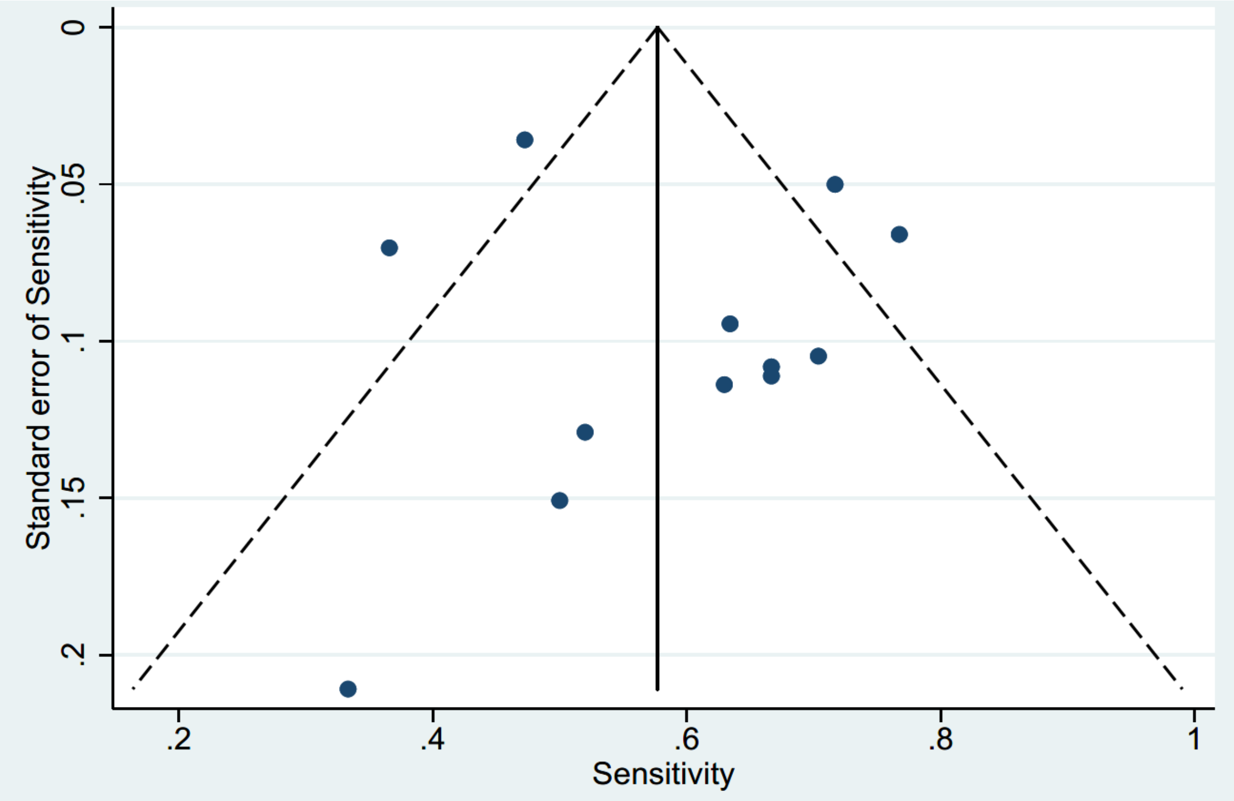

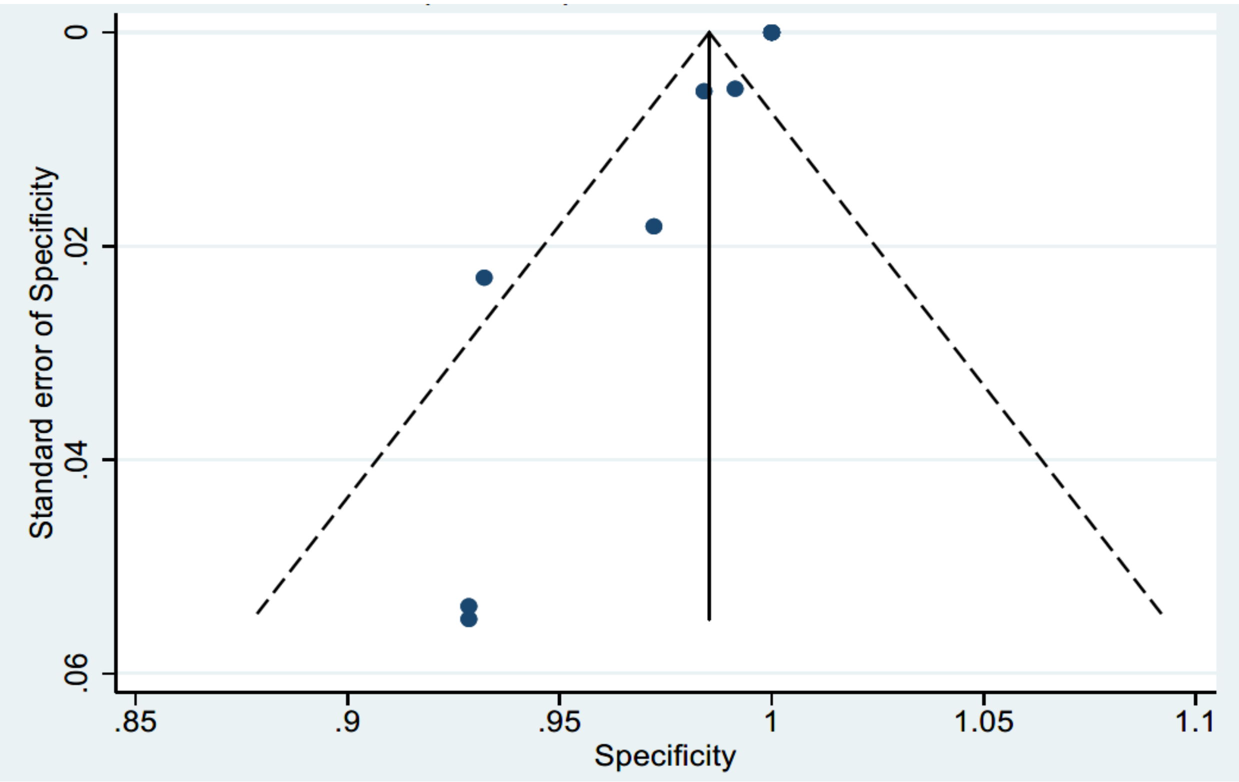

Figure 19: Funnel plots with pseudo 95% confidence limits of E1L3N LDT (candidate) vs. Ventana PD-L1 (SP263) (GS) for 50% TPS Cut-

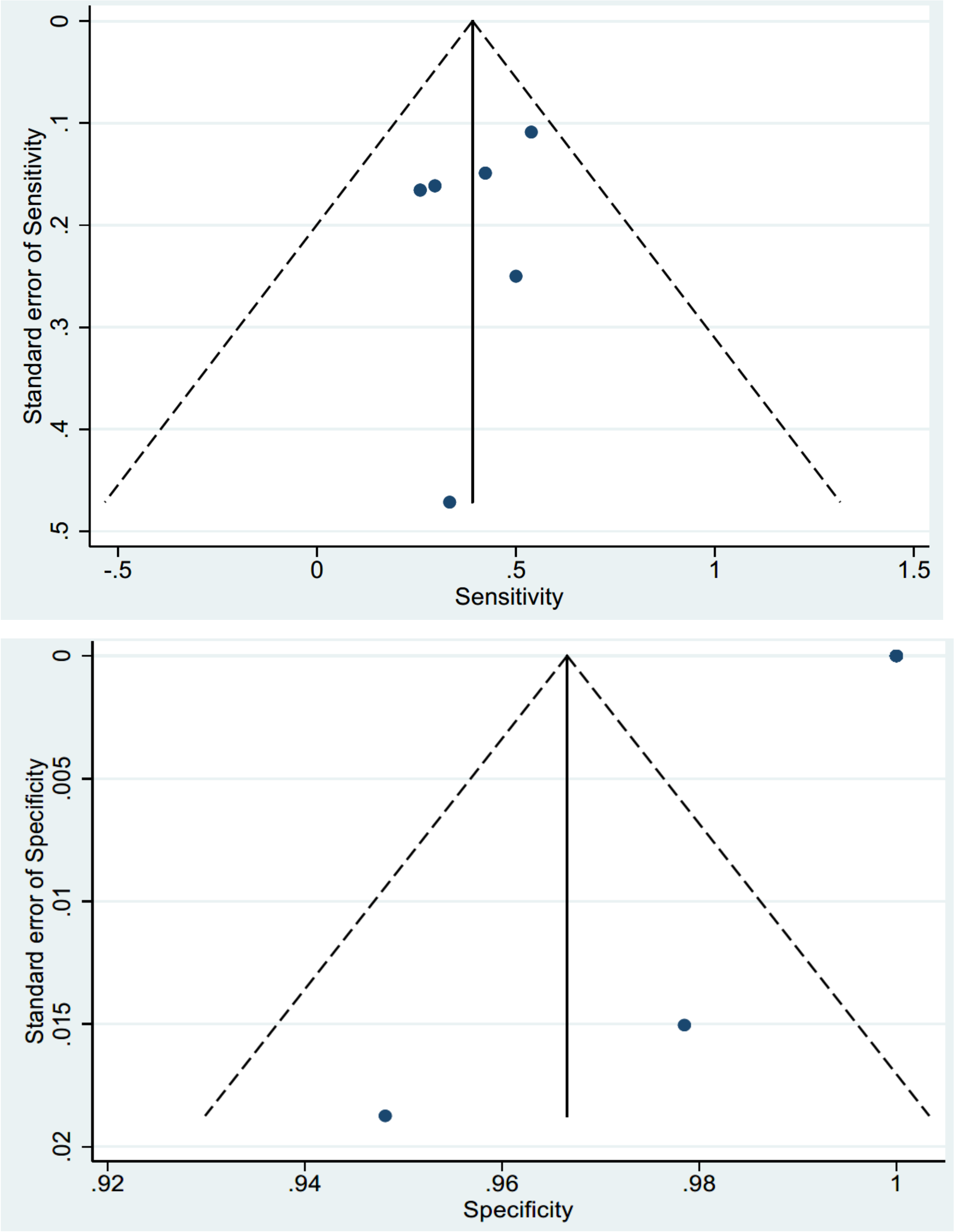

Figure 20: Funnel plots with pseudo 95% confidence limits of E1L3N LDT (candidate) vs. Ventana PD-L1 (SP263) (GS) for 1% TPS Cut-off

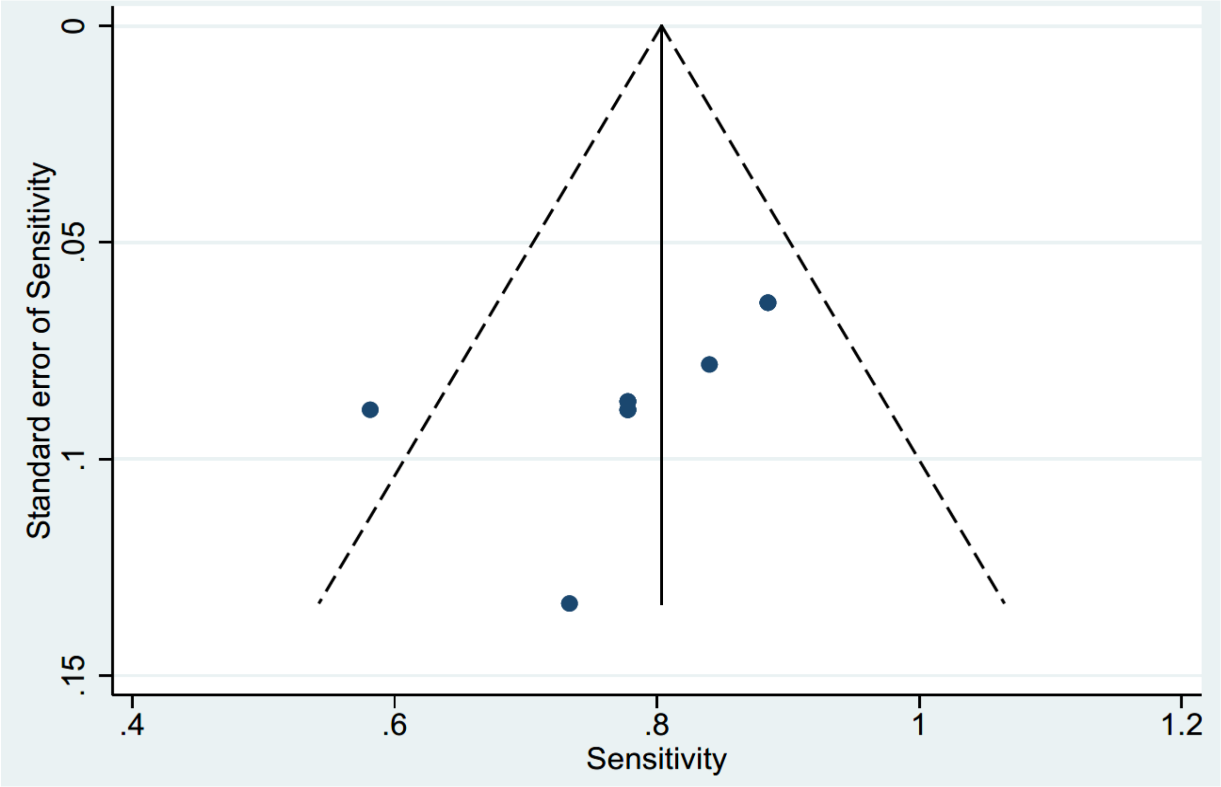

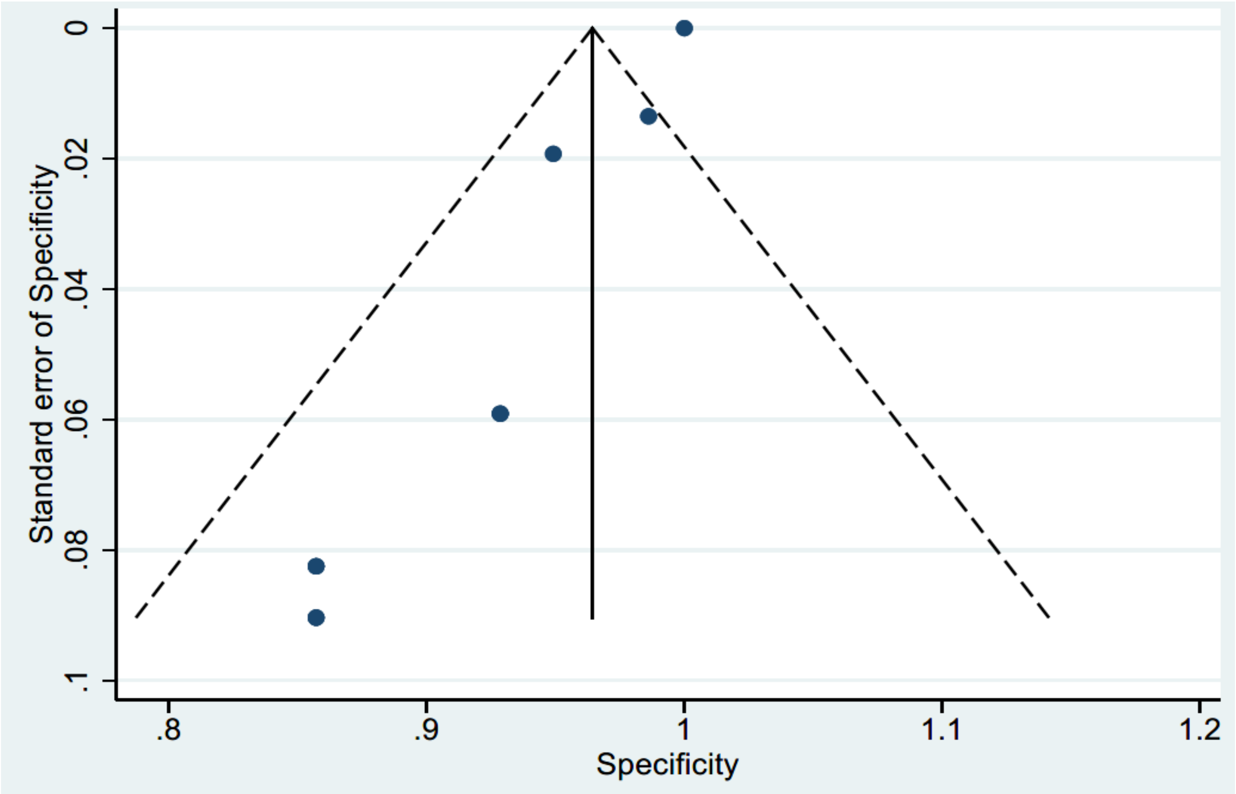

Figure 21: Funnel plots with pseudo 95% confidence limits of 28-8 LDT (candidate) vs. PD-L1 IHC pharmDx 28-8 (GS) at 1% TPS Cut-off

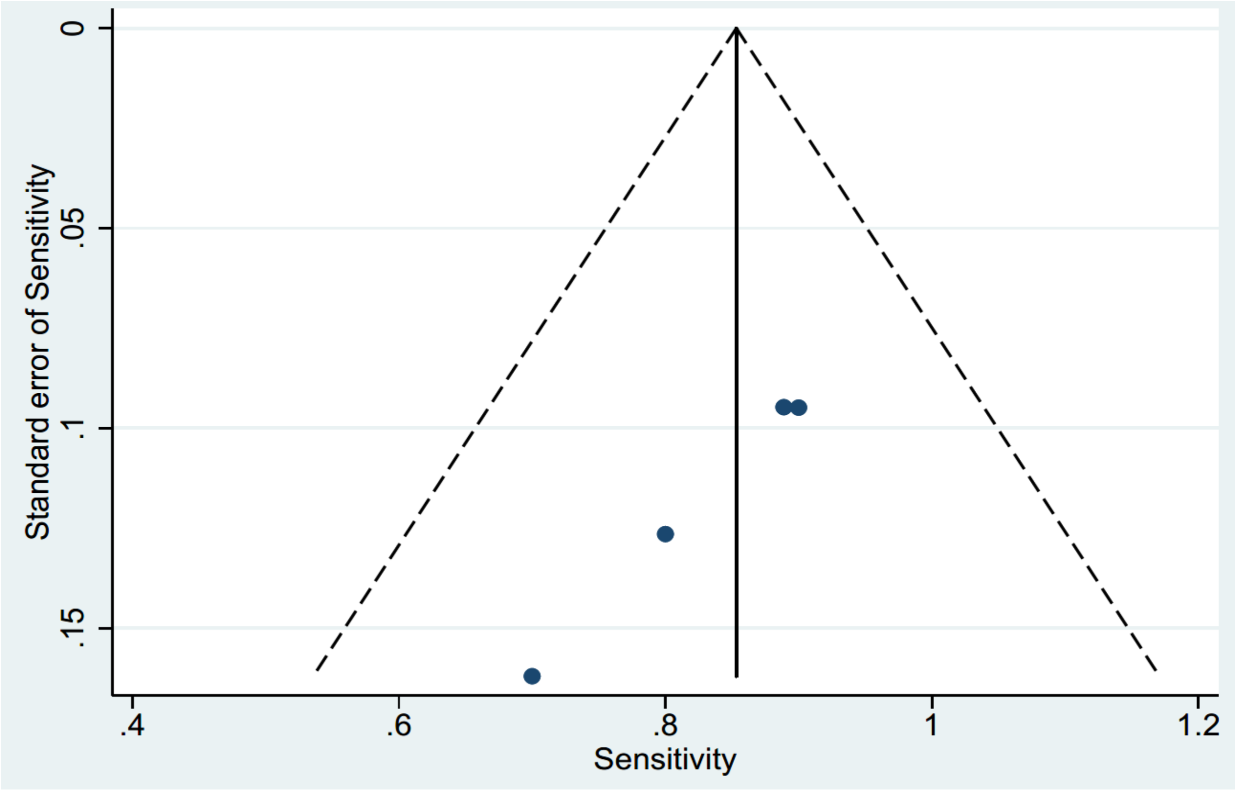

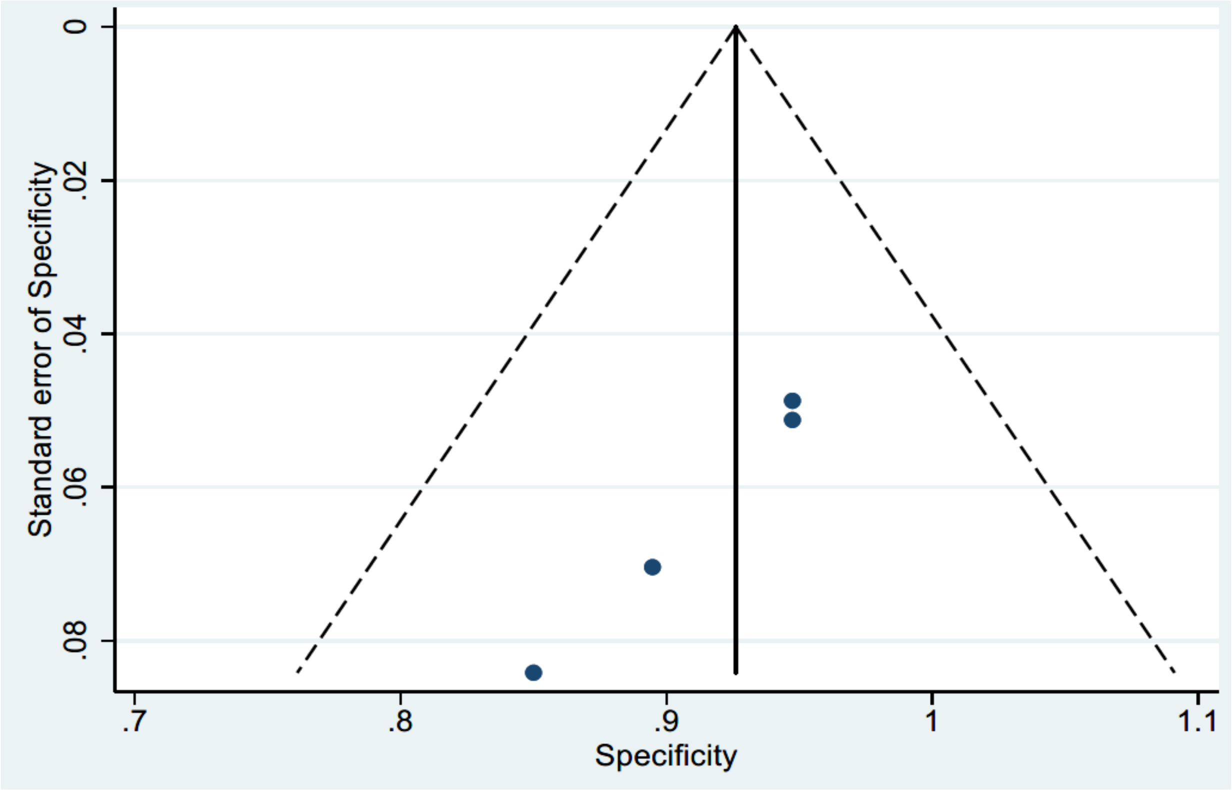

Figure 22: Funnel plots with pseudo 95% confidence limits of 28-8 LDT (candidate) vs. PD-L1 IHC pharmDx 22C3 (GS) at 50% TPS Cut-off

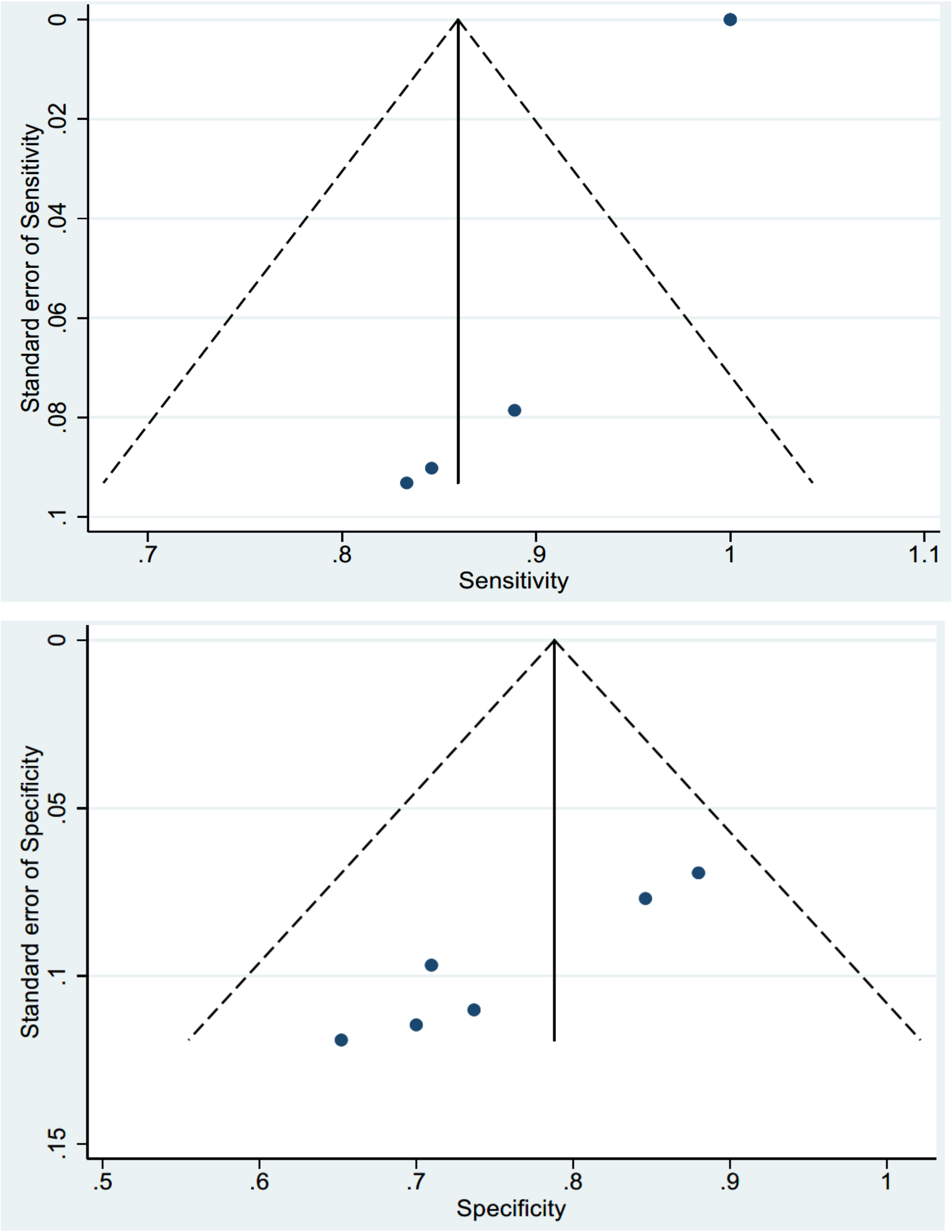

Supplement: Supplementary file 5 — Funnel Plots [file 41379_2019_327_MOESM5_ESM.pdf]
